# Leap from Diradicals to Tetraradicals by Topological Control of π-Conjugation

**DOI:** 10.1021/acs.joc.4c01375

**Published:** 2024-09-20

**Authors:** Sergi Betkhoshvili, Jordi Poater, Ibério de P. R. Moreira, Josep Maria Bofill

**Affiliations:** † Departament de Química Inorgànica i Orgànica & IQTCUB, Universitat de Barcelona Martí i Franquès 1−11, Barcelona 08028, Spain; ‡ Departament de Ciència de Materials i Química Física & IQTCUB, Universitat de Barcelona, Martí i Franquès 1−11, Barcelona 08028, Spain; § ICREA, Pg. Lluís Companys 23, Barcelona 08010, Spain

## Abstract

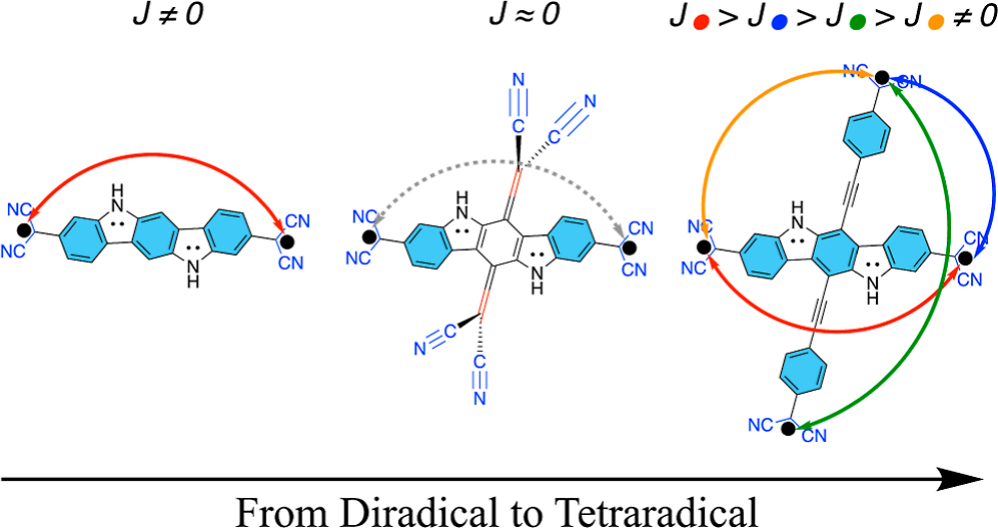

In this work, we explore the series of diradical(oid)s based on 2,2′-(5,11-dihydroindolo[3,2-*b*]carbazole-3,9-diyl)dimalononitrile (further referred to as **PH**). Hydrogen atoms in the central benzenoid (CB) ring of **PH** are substituted by the series of substituents with various lengths of π-conjugated chain and electron-donating or electron-withdrawing properties to study how they modulate the diradical character of the parent compound. The diradical character of molecules increases up to 88–89% by two groups doubly bonded to both sides of the CB ring of **PH** in *para* relative positions. This breaks the direct π-conjugation between unpaired electrons that gives rise to two radical centers and restricts the minimal polyradical identity of the compound to diradical. We show that diradicals and tetraradicals can be designed, and their polyradical character can be modulated by controlling the topology of π-conjugation as long as there is sufficient aromatic stabilization. Henceforth, the bridge between diradicals and tetraradicals is established, leading to the tetraradical(oid) molecule, which has been predicted to have narrow low-spin to high-spin energy gaps in our recent Letter.

## Introduction

**Figure 1:**
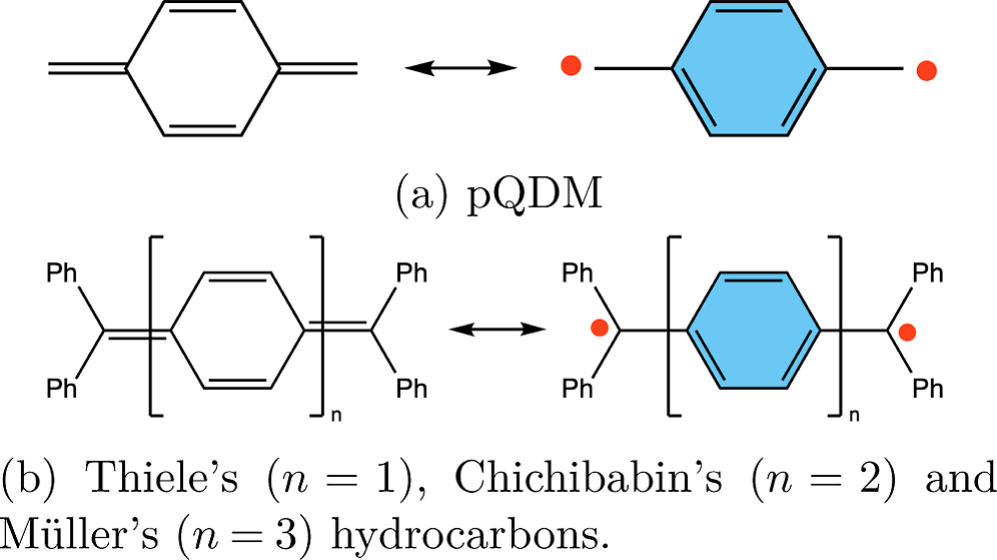
Resonance structures of (a) *para*-quinodimethane (pQDM) and (b) Thiele’s, Chichibabin’s, and Müller’s hydrocarbons.

Biradicals/diradicals are molecules that have two unpaired electrons. They have a wide range of applications, such as in organic electronics and spintronics, *n*-channel or ambipolar field effect transistors, organic magnetic materials, molecular switches, singlet fission with solar energy conversion capability, batteries, nonlinear optics, functional dyes, photodynamic therapy, and quantum technologies.^1−20^ As open-shell molecules, diradicals are expected to be both kinetically and thermodynamically unstable. In organic biradicals, a common stabilizing factor is a delocalization in the π-system and aromaticity. *para*-Quinodimethane (pQDM) is the primitive building block that exemplifies this concept (see Figure 1a). Thiele’s hydrocarbon was the first diradical(oid) that was synthesized in 1904,^21^ soon followed by Chichibabin’s hydrocarbon in 1907,^22^ and Müller’s hydrocarbon in 1941^23^ (see Figure 1b). As is apparent from Figure 1, there are quinoidal and aromatic resonance structures, the latter corresponding to a biradical.

According to the IUPAC Compendium of Chemical Terminology (“Gold Book”),^24^ the term “biradical” usually refers to the molecules where two unpaired electrons act independently or almost independently. Thus, “biradical” is best understood as two doublets in the molecule. When two unpaired electrons interact strongly enough to produce two spin states: singlet (*S* = 0) and triplet (*S* = 1), the molecule is technically referred to as “diradical”. In this work, as in the majority of the scientific literature, the terms “diradical(oid)” and “biradical(oid)” are used interchangeably.

**Figure 2:**
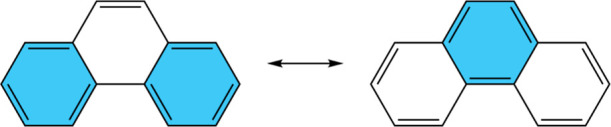
Resonance structures of phenanthrene: one on the left contributes more to the resonance hybrid according to Clar’s rule.

Aromaticity, a useful chemical concept and not an observable, is somewhat imprecisely defined. Hückel’s rule defines monocyclic molecules with a maximum number of conjugated bonds (annulenes, C_*n*_H_*n*_) with *D*_*nh*_ symmetry and 4*n* + 2 π electrons as aromatic, and with *D*_*nh*_ symmetry and 4*n* π electrons as antiaromatic (where *n* is a positive integer). Other extended rules of aromaticity apply not only to monocyclic but to polycyclic hydrocarbons and even in three dimensions.^25^ For benzenoids [polycyclic aromatic hydrocarbons (PAHs) containing only six-membered rings], a very useful model is the π-sextet rule proposed by Clar in 1972,^26^ which considers aromaticity as a local property of the benzene-like six-membered rings with π-sextets that are separated from adjacent rings by C–C single bonds. Clar’s rule can be used to understand the properties and even reactivity of compounds.^27,28^ Clar’s rule states that the resonance structure, which has the most π-sextets, will be the most important contributor to the resonance hybrid of a PAH. For example, in the case of phenanthrene shown in Figure 2, the resonance structure with two Clar’s π-sextets has a greater contribution in the “resonance hybrid”. A reader can refer to the recent comment^25^ from Prof. Solà to grasp the extensions and generalizations of aromaticity rules in much greater detail.

The design of diradical(oid)s is usually based on ideas of proaromaticity and/or antiaromaticity. If a nonaromatic π-conjugated molecule tends to become aromatic in a diradical or zwitterionic form, it can be called pro-aromatic, like pQDM, Thiele’s, Chichibabin’s, and Müller’s hydrocarbons (Figure 1). Exemplifications of increased diradical character due to enhanced aromatic stabilization are commonplace in the relevant scientific literature.^4,5,29,30^ Usually, as a polycyclic quinoidal pro-aromatic compound includes more benzenoid rings, the diradical character of the compound increases.^4,5,29,31^ The greater number of aromatic rings offsets the destabilizing factor of an open-shell character by the aromatic resonance energy.^4,5,29,31^ This strategy of diradical design has also been extended to heterocyclic aromatic moieties such as thiophenes^13,31^ and pyrroles.^13,29,31^ Furthermore, antiaromatic molecules exhibit diradical character in the series of compounds based on a set of antiaromatic rings.^4,13,31,32^ Hence, modulation of the diradical character can be a convoluted and molecule-specific effect.

As pro-aromatic molecules can be induced to become diradicals by including more pro-aromatic rings, we extend this idea to design tetraradicals. If the cumulative aromatic stabilization is sufficient, then the molecule could possess a substantial tetraradical character. Recently, our group found that by merging two diradical(oid)s with aromatic stabilization while maintaining π-conjugation throughout the resulting structure, we obtain a cross-conjugated tetraradical(oid) **PTP** (see Figure 4) with thermally accessible high-spin states.^33^

**Figure 3:**
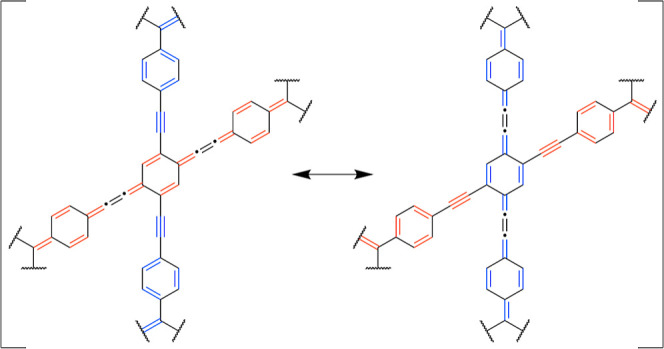
Illustration of cross-conjugation with an organic framework. π-Systems crossing each other in the CB ring are shown in red and blue.

**Figure 4:**
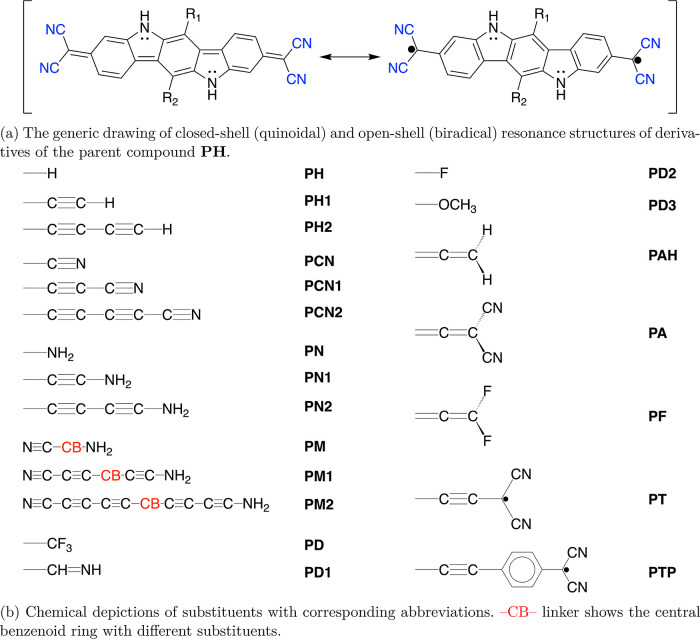
Set of molecules that have been studied in this work with their abbreviations.

Research on higher-order polyradicals started in 1964 by first synthesizing triradical^34^ and tetraradical,^35^ after which many theoretical and experimental studies followed.^6,33,36−61^ Diradicals, tetraradicals, and polyradicals are magnetic molecules/materials which can be used in quantum computing as qubits and qudits (higher-order qubits).^62^ There are many challenges to overcome in quantum computing, one of which is coherence times.^20^ A higher number of spin states opens new possibilities in quantum computing and error correction.^63^

Considering the broad current and future applications of diradicals, tetraradicals, and polyradicals, it is especially important to establish some guidelines to design and predict the properties of these species. This work focuses on increasing the diradical character of the compound by using different substituents, controlling the topology of π-conjugation, and extending these structures to tetraradicals.

The compounds studied in this work share a common parent compound, 2,2′-(5,11-dihydroindolo[3,2-*b*]carbazole-3,9-diyl)dimalononitrile (**PH**), shown in Figure 4. The compound **PH** was computed and experimentally verified^64,65^ to have a moderate diradical character. In our previous work, we computationally verified that Ovchinnikov’s rule,^66^ which predicts spin configurations in organic open-shell molecules, is accurate for these systems and henceforth established guidelines for the fully π-conjugated polyradical design as follows: (1) upon designing polyradicals by merging smaller (poly)radicals, full π-conjugation must be maintained throughout the resulting structure. (2) Within a polyradical, each diradical subunit must have aromatic stabilization from at least two or (more favorably) three benzene rings (Clar’s π-sextets). (3) Aromatic rings can be shared between different diradical subunits within a polyradical. (4) Upon designing a polyradical, one can restrict the lower bound of polyradical character by cross-conjugation.^33^ We define cross-conjugation as follows: if in some valence bond forms (VBFs, i.e., Lewis structures), two terminal sites of the molecule are continuously connected by alternating π bonds, this would forbid a continuous connection via alternating π bonds for at least one different pair of terminal sites. Since such conjugated π chains intersect or cross each other, we call such systems cross-conjugated. For illustration, we can refer to Figure 3, which includes a generic (closed-shell) cross-conjugated organic framework where the π-systems intersect/cross each other in the central benzenoid (CB) ring. In polyradicals, which have all of their unpaired electrons conjugated to the π-system of the molecule, this means that if in some VBF a given unpaired electron closes the shell with another unpaired electron by continuous/direct π-conjugation, this would forbid closing at least one shell by another pair of directly π-conjugated unpaired electrons. For example, the tetraradical(oid) **PT** shown in Figure 8 exhibits cross-conjugation since closings of the shells via direct π-conjugation for different pairs of unpaired electrons (in one branch or the other) are mutually exclusive. Henceforth, it is important to explore the species that follow some of the established guidelines and identify the factors that allowed us to bridge the gap between diradicals and tetraradicals.

**Figure 5:**
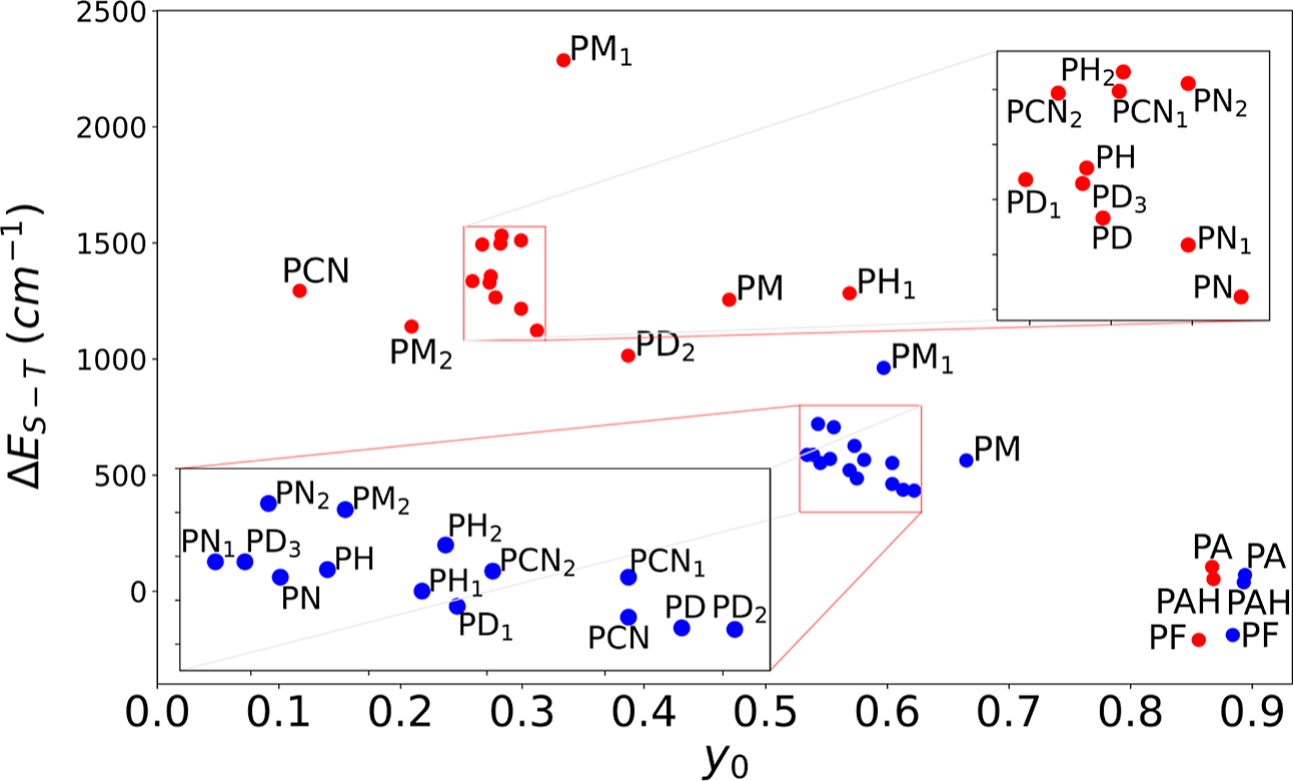
Calculated diradical characters (*y*_0_) and singlet–triplet (Δ*E*_S–T_) gaps (in cm^–1^) based on the results of CASSCF calculations.

**Figure 6:**
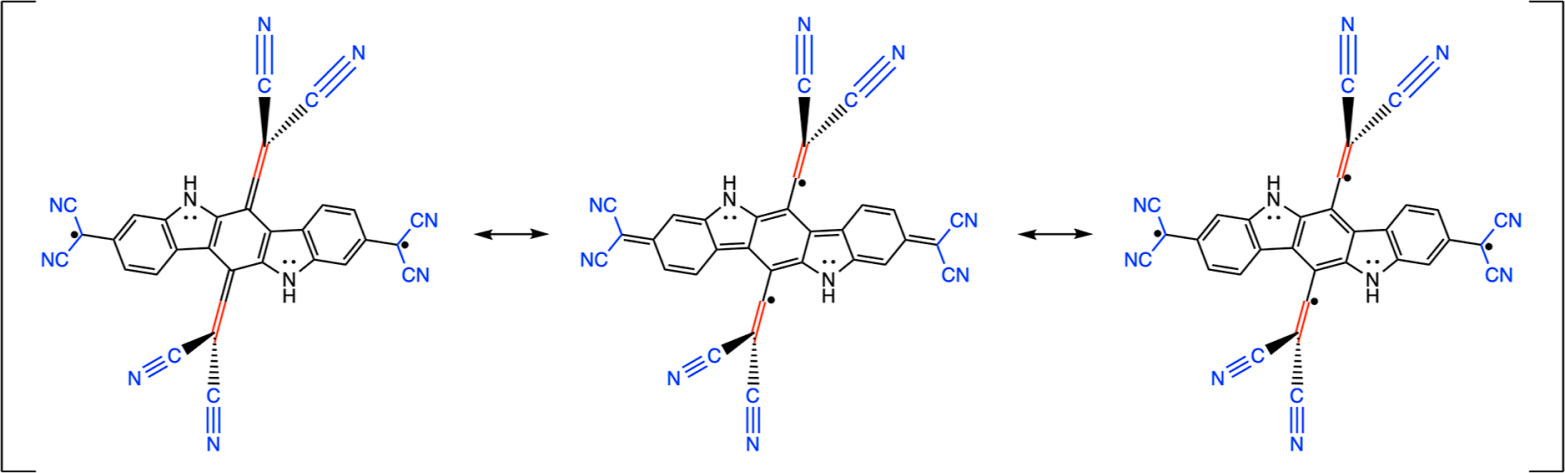
Biradical (left and middle) and tetraradical (right) resonance structures of the dicyano-allenic derivative of **PH**, denoted as **PA**. The double bond indicated in red emphasizes that its π-system is perpendicular to the π-system of the indolo[3,2-*b*]carbazole.

**Figure 7:**
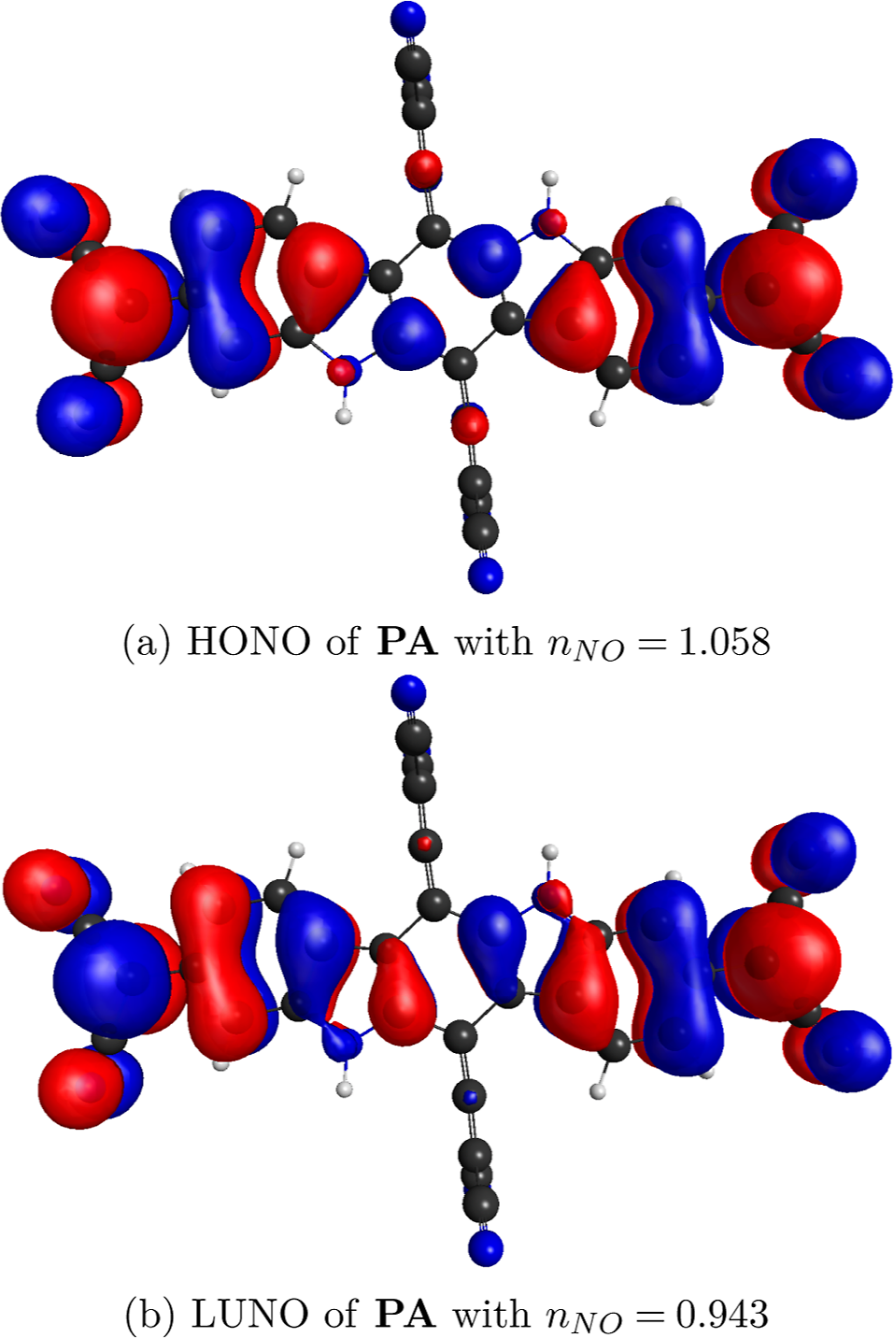
HONO (a) and LUNO (b) from CASSCF(14,14) calculation of S_0_ state of **PA**. Isosurfaces with the value of 0.015.

**Figure 8:**
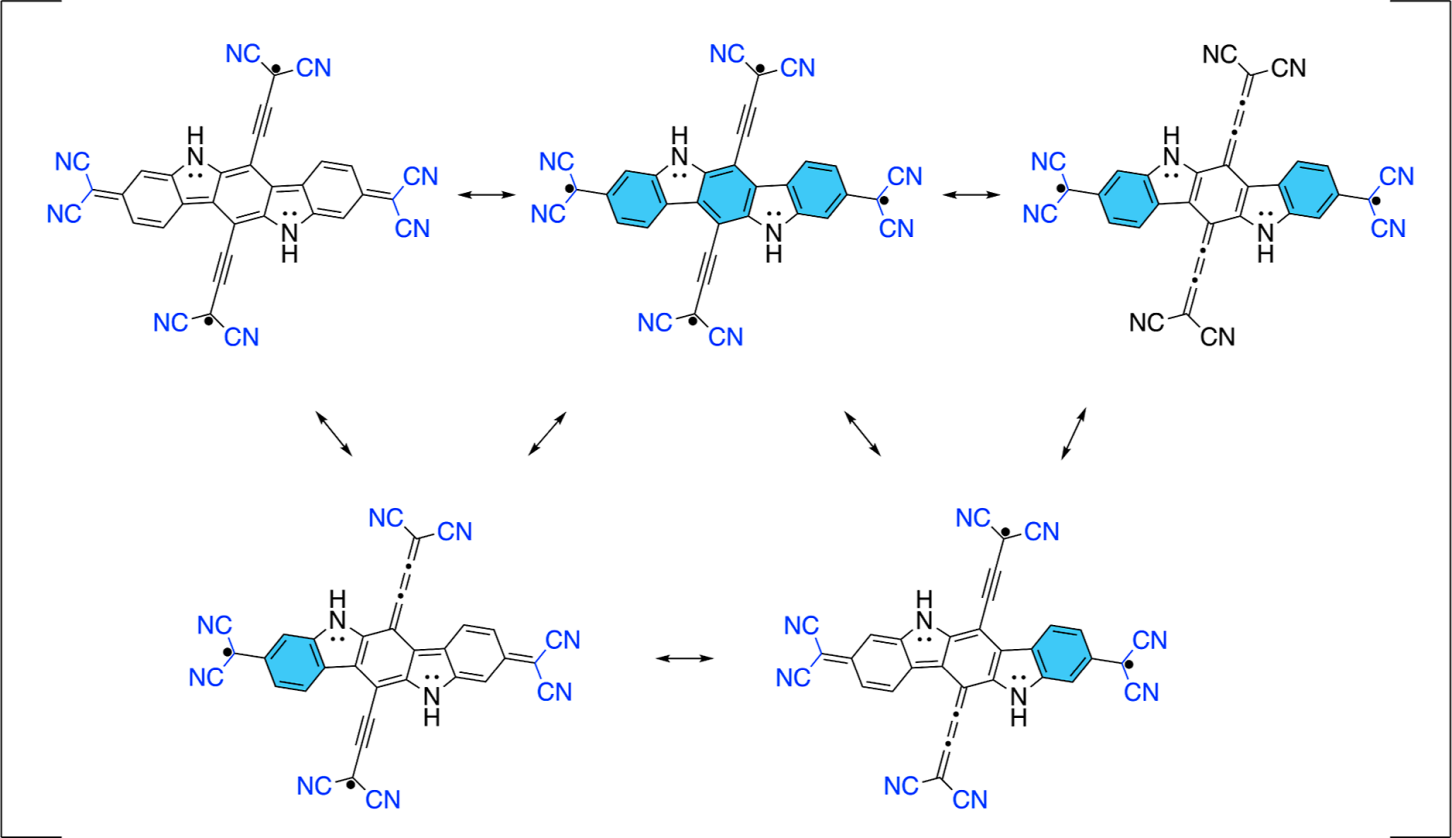
Resonance structures of **PT**.

## Methods and Computational Details

For geometry optimizations of the molecules, density functional theory (DFT) was used with the Amsterdam Density Functional (ADF) software code.^67^ Since geometry can be an important variable in determining the electronic structure [this is the reflection of the existence of potential energy surface (PES)] of molecules, in this work, identical calculations were done on both: closed-shell singlet-optimized geometry (CSG) as a result of restricted Kohn–Sham (RKS) DFT calculations, and triplet-optimized geometry as a result of unrestricted Kohn–Sham (UKS) triplet DFT [triplet geometry (TG)].^68−70^ Slater-type orbitals triple-ζ basis set (TZP)^71^ was used for all ADF/DFT calculations for all compounds. Moreover, vibrational frequencies were computed for each optimized geometry in order to verify whether or not they correspond to the local minimum on PES.^72−74^ From now on, CSG will be referred to as CSG, and triplet-optimized geometry will be referred to as TG. In some cases, it was necessary to optimize geometry for a quintet spin state, and this optimized geometry is referred to as QG. Except for benchmark calculations of different states of **PH** with different exchange–correlation functionals, generalized gradient approximation exchange–correlation functional BLYP^75,76^ was used. Notably, studying the electronic structures of biradical(oid)s that are intrinsically multiconfigurational is unreliable with DFT. Empirical proof of this claim on **PH** is shown in Table S1a in the Supporting Information.

The Hartree–Fock method with its restricted and unrestricted formalism (RHF and UHF, respectively) is a computationally cheap tool to evaluate if the molecule has an open-shell electronic structure. To achieve this, we need to compute the RHF and UHF solutions separately and compare them. If the UHF solution has lower energy than the RHF solution (it can have the same or lower energy only), it means that the closed-shell electronic wave function is unstable, and the ground-state wave function is stable with an open-shell UHF solution. For most Hartree–Fock calculations, we used Gaussian 2016 software.^77^ The basis set used for Hartree–Fock calculations was Dunning’s correlation-consistent double-ζ basis set cc-pVDZ.^78^

To study the multiconfigurational wave function of diradical(oid)s and tetraradical(oid)s presented in this work, a multireference method such as complete active space self-consistent field (CASSCF), which is also known as the full optimized reaction space, was used.^79−85^ CASSCF calculations were performed using the General Atomic and Molecular Electronic Structure System (GAMESS).^86^ In most cases, the initial guess orbitals for CASSCF calculations were UHF natural orbitals (NOs) (hence, some HF calculations were performed in GAMESS), as they are one of the best starting orbitals for CASSCF.^87−89^ For CASSCF calculations, as for Hartree–Fock calculations, the cc-pVDZ basis set was used. For diradical(oid) molecules, in most of the cases, the active space of 14 electrons in 14 orbitals CASSCF calculations, CASSCF(14,14), was sufficient based on the occupation numbers of UHF NOs.^87,89^ For tetraradical(oid) **PT**, CASSCF(4,4) and (12,12) and for tetraradical(oid) **PTP**, CASSCF(4,4), (10,10), (14,14), and (16,16) calculations were performed^33^ to describe the so-called nondynamic correlation. In order to explore the wave function of tetraradical(oid)s **PT** (Figure 4) in greater detail, CAS configuration interaction (CASCI) calculations with (4,4) and (12,12) active spaces were performed. For **PTP**, the results of CASCI(4,4) and CASCI(16,16) calculations are given in our recent Letter.^33^

To describe the local aromaticity of benzenoid rings in these compounds, the multicenter index (MCI) of aromaticity, which describes the degree of electron-sharing within rings, was calculated.^90,91^ For a characterization of “global aromaticity”, anisotropy of the induced current density (ACID) method was used.^92,93^ ACID describes the response of electron currents on a perpendicular magnetic field, which leads to the flow of electron current around the aromatic rings. See Section S2.2 for the proper procedure of approximating properties of multiconfigurational systems with the monoconfigurational methods UHF and UKS-DFT.

For calculations of diradical and tetraradical character indices, Yamaguchi’s approach was employed^94^ based on occupation numbers (*n*_NO_) of NOs, which are eigenvectors of the first-order density matrix operator.^95^ The *n*-radical character *y*_*n*_ varies from *y*_*n*_ = 0, meaning no *n*-radical character, to *y*_*n*_ = 1, meaning full *n*-radical character. The highest occupied natural orbital (HONO) is defined as the orbital that has the lowest *n*_NO_ among the NOs with *n*_NO_ ≥ 1. The lowest unoccupied natural orbital (LUNO) is defined as the orbital that has the highest *n*_NO_ among NOs with *n*_NO_ ≤ 1. In UHF, *n*_HONO–*i*_ + *n*_LUNO+*i*_ = 2.000, which is usually also manifested in CASSCF and CASCI NOs. 2(*i* + 1)-ple radical character is calculated as follows
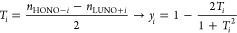


By substituting *i* = 0, we obtain *y*_0_ and 2(0 + 1)-ple radical character. Hence, *y*_0_ is a diradical character index. Analogically, by substituting *i* = 1, we obtain *y*_1_, which is a tetraradical character index.

Energy gaps between different states are calculated as follows: singlet–triplet gap Δ*E*_S–T_ = *E*_T_ – *E*_S_, triplet–quintet gap Δ*E*_T–Q_ = *E*_Q_ – *E*_T_, and singlet–quintet gap Δ*E*_S–Q_ = *E*_Q_ – *E*_S_.

For the characterization of interactions between radical centers, the Heisenberg–Dirac-van Vleck (HDvV) model Hamiltonian (96−99) has been built and corresponded to an effective Hamiltonian (100,101) to calculate exchange-coupling constants (*J*_*ij*_) between radical centers. The theoretical background and calculation details are given in Section S3.3 in the Supporting Information.

## Results and Discussion

The set of compounds derived from **PH** is shown in Figure 4. **PH** has two radical terminal moieties, which are bridged by aromatic rings of indolo[3,2-*b*]carbazole. These radical centers are directly π-conjugated so that there is a dichotomy between closed-shell quinoidal and diradical aromatic electronic configurations, as shown in Figure 4a. The substituents R_1_ and R_2_ are connected to the CB ring of **PH** in relative *para* positions from one another. The structures of most derivatives (except **PAH**, **PA**, and **PF**) can be described by two sets. The first set from **PH** to **PM2** has the structure L_1_–(≡)_*n*_–CB–(≡)_*n*_–L_2_ (and L_*i*_–(≡)_*n*_ means R_*i*_), where *n* = 0, 1, 2, and L_*i*_ are groups H, C≡N, and NH_2_. We have two cases that define the dipole moment of these molecules: if L_1_ = L_2_, we have the structure with *C*_2*h*_ symmetry and zero dipole moment, and if L_1_ ≠ L_2_, we have the structure with *C*_*S*_ symmetry and nonzero dipole moment. For the second set of derivatives, **PD**–**PD3**, **PT**, and **PTP**, the general structure is R_1_–CB–R_2_, and R_1_ = R_2_ = −CF_3_, −CH=NH, −F, −OCH_3_, , and , respectively. For **PAH**, **PA**, and **PF**, the general structure can be expressed as M_2_C=C=CB=C=CM_2_, where M = H, C≡N, and F, respectively. Through these series of substituents, which vary in length of the π-conjugated chain and electron-donating or electron-withdrawing properties, we modulate the dipole moment, length of the π-system perpendicular to the quinoidal π-chain, average electron density of the indolo[3,2-*b*]carbazole subsystem, and the topology of π-conjugation. Notably, substituents vary from strongly electron-withdrawing (CN) to strongly electron-donating (NH_2_), with several substituents that fall between these two extremes. In addition, these substituents are very common in organic compounds, and it is therefore favorable to study these realistic systems. Besides, the effect of inserting acetylene/ethynyl residues as bridging groups has not been explored in depth. Thus, this additional variance might give us enhanced insight into the mechanism of modulating the open-shell characters of the molecules. By using these substituents to obtain different derivatives of **PH** while maintaining aromatic stabilization, we created cross-conjugated tetraradical(oid)s **PT** and **PTP** (latter already described in our recent Letter^33^) by bonding two π-conjugated radical moieties to the CB ring of **PH**.

### Diradical Character for Derivatives from PH to PD3

As a preliminary measure, we performed the DFT benchmark for diradical(oid) **PH** with different exchange–correlation functionals. These calculations showed sensitive dependence of energy gaps and ground state properties on the used exchange–correlation functional (Table S1a in the Supporting Information). Since Hartree–Fock is a computationally cheap method to check if the electronic structure is open-shell, we applied this method to all the derivatives of **PH**. The results comparing differences in the energy of closed-shell solution (RHF) and most stable open-shell solutions (*E*_CSS_ – *E*_OSX_) for derivatives of **PH** (Figure 4) are given in Table S1b in the Supporting Information. These results show that all compounds presented in this work have an open-shell electronic structure from the standpoint of the Hartree–Fock formalism. Also, for most cases, the lowest-energy solution was a UHF broken-symmetry singlet (obtained by mixing the highest occupied molecular orbital and the lowest unoccupied molecular orbital in the initial guess), but for three instances, **PN**, **PN1**, and **PD3**, the lowest-energy solution was a UHF triplet.

Both singlet and triplet UHF NOs indicate the presence of unpaired electrons on carbons indicated in the diradical resonance structure of Figure 4a and on the atoms that are in successively allylic positions from it. Notably, the check of the RHF wave function showed RHF → UHF instability and then a stable UHF wave function (singlet or triplet) as a ground state. As expected from the structure, Hartree–Fock results predict **PA** to have the largest RHF → UHF instability, amounting to 125 kcal/mol more stability than a UHF singlet relative to the RHF solution.

CASSCF results exclusively show the singlet open-shell ground state for these compounds, while Hartree–Fock shows the triplet ground state for only three derivatives with electron-donating substituents. Importantly, the results show that a higher diradical character does not necessarily mean a smaller singlet–triplet gap (Δ*E*_S–T_). The notion of smaller Δ*E*_S–T_ translating into higher diradical character has been repeatedly demonstrated for Kekulé diradical(oid)s in the research and review articles of refs.,^4,5,29,32,64,102−105^ in which Δ*E*_S–T_ is a proximal parameter based on which the degree of diradical character is estimated.^29,106^

Using CASSCF results, diradical character indices (*y*_0_) and singlet–triplet gaps (Δ*E*_S–T_) have been computed. The results, which are given in Figure 5 for both closed-shell geometry (CSG) and TG, show that there is a significant change upon switching from CSG to TG. Furthermore, to gain insight into the strengths of exchange-coupling between radical centers for these compounds, we used results from CASCI calculations (mostly (14,14)) to build an effective Hamiltonian^100,101,107^ for each compound. We corresponded it to the HDvV model Hamiltonian^96−99^ in order to calculate the exchange-coupling constant between radical centers. For diradicals, the exchange-coupling constant *J*_12_ between two radical centers corresponds to Δ*E*_S–T_ from CASCI calculations if the results are obtained with a proper procedure (see Section S3.3 in the Supporting Information). For diradical(oid)s presented in this study, a scatter plot and table of exchange-coupling constants as a function of the identity of the compound are given in Figure S8 of the Supporting Information. Similarly to the lack of a strict correlation between *y*_0_ and Δ*E*_S–T_, the values of *y*_0_ and *J*_12_ do not correlate. The relation between the singlet–triplet gap and degree of diradical character has been established in Thiele-like compounds (see Figure 7 of ref (105) for illustration). In spite of equivalence to Thiele-like compounds in terms of π-conjugation,^4,5,29,32,64,102−105^ this correlation cannot be established for these indolo[3,2-*b*]carbazole-based compounds, which makes them categorically different in this aspect. The reason for this discrepancy is not entirely obvious. It might be that the two derivatives of **PH** are more different in the set of compounds we studied than Thiele-like compounds that have been characterized to have a well-established relation between Δ*E*_S–T_ and some version of *y*_0_. Indeed, in our set of substituents, the variance is not only in the electron-withdrawing or electron-donating effects of substituents, as in most studies, but also in the length of their π-conjugated chain and even dipole moment. Nevertheless, even if we focus on the series, which differ only by one acetylene residue, it is clear that they do not show any simple correlation across multiple series between diradical character and singlet–triplet gap, as shown in Figure 5.

There is a similar lack of correlation if we focus on the same number of acetylene residues and vary the substituents. The tendency of the change of diradical character does not strictly match the tendency of the change of the singlet–triplet gap, even by modifying the dipole moment. Henceforth, we might gain more insight if we focused on one of these two properties.

In order to establish trends in the modulation of the diradical character in these compounds, let us first refer to studied diradical(oid)s that do not have acetylene residues between substituents and the CB ring. The approximate ordering in the electron-withdrawing character of the substituents is as follows: CN, CF_3_, F, H, −CH=NH, −OCH_3_, NH_2_, and the diradical characters vary as follows: 0.117, 0.278, 0.387, 0.274, 0.259, 0.273, and 0.312 for closed-shell geometry, while for triplet-optimized geometry, these values are 0.604, 0.613, 0.622, 0.553, 0.575, 0.539, and 0.545, respectively. It is clear that there is no decipherable trend in the variation of diradical character as a function of electron-withdrawing or electron-donating properties of the substituents in the CB ring. Let us explore whether we can find any trend in variation of diradical character upon modifying the number of acetylene residues between substituents and the CB ring. Such series are **PH**–**PH2**, **PCN**–**PCN2**, **PN**–**PN2**, and **PM**–**PM2**. Diradical characters for closed-shell singlet geometries (and triplet geometries given in parentheses) for **PH**–**PH2** vary as follows: 0.274 (0.553), 0.569 (0.569), and 0.283(0.573). For **PCN**–**PCN2**, *y*_0_ varies as follows: 0.117 (0.604), 0.282 (0.604), and 0.267 (0.581). For **PN**–**PN2**, *y*_0_ varies as follows: 0.312 (0.545), 0.299 (0.534), and 0.299 (0.543), while for **PM**–**PM2**, *y*_0_ varies as follows: 0.470 (0.665), 0.334 (0.597), and 0.209 (0.556). Such a variance is not sufficient to clearly state the significant tendency of diradical character modulation. Moreover, the same series does not show a clear trend in variation of singlet–triplet gap upon systematically changing the substituents in the CB ring of the parent compound. Also, the variance of diradical character does not strictly correlate to the corresponding variance in singlet–triplet gaps, even within these series of very similar compounds.

Furthermore, if the molecule in this set has a nonzero dipole moment, this dipole moment does not induce any substantial charge transfer between radical centers; hence, it should not affect diradical character much more than having substituents, which make the dipole moment of the molecule zero. This is precisely what we observe in the compound set we have studied, as compounds with nonzero dipole moment (**PM1–2**) do not differ significantly from many other molecules with zero dipole moment in terms of diradical character and singlet–triplet gap. The effect of the dipole moment would be very significant if there was substantial charge transfer from one radical center to another. Since diradical means two unpaired electrons, this charge transfer would lower the diradical character of the compound.

In addition, this lack of strict correlation between *y*_0_ and Δ*E*_S–T_ might be the effect of the presence of heteroatoms (nitrogen) in the pyrrole rings between the radical centers. Also, the compounds studied in this work possess “global aromaticity” (except **PAH**, **PA**, and **PF**) around the perimeter of indolo[3,2-*b*]carbazole, while Thiele-like compounds do not. Moreover, we must keep in mind that *y*_0_ is calculated from the occupation numbers of HONO and LUNO, only two orbitals, while Δ*E*_S–T_ depends on the full electronic structure of the molecule in each state. For example, a molecule can have a triplet state with lower energy than an open-shell singlet state such as diradical **PF**, but HONO and LUNO of the S_0_ state do not have their occupation numbers exactly 1.000. Hence, **PF** has a lower diradical character than 1 according to index *y*_0_ from Yamaguchi.^94^

However, since a triplet is fully diradical by electronic configuration, if the ground state of the molecule with two possible unpaired electrons is a triplet, then the diradical character of this molecule must be 1, by definition. Nevertheless, since we are using a two-orbital measure of diradical character (*y*_0_) from state S_0_, it is not guaranteed that it will be in complete correspondence to the singlet–triplet gap, which depends on the full electronic structure of the molecule.

It is noteworthy that differences in CSG and TG geometries between **PH** derivatives are not dramatic and are usually ≤0.01 for corresponding bonds. However, certain bonds do pronounce the difference between closed-shell quinoidal and diradical aromatic resonance structures depicted in Figure 4a more significantly (comparisons are given in Figures S9 and S4 of the Supporting Information).

The bond-length differences between CSG and TG follow the trend: if a bond is double in a quinoidal resonance structure (for example, between dicyanomethylene carbon and the indolo[3,2-*b*]carbazole moiety), CSG gives its length to be about 0.01 Å shorter. However, if a bond is single in the quinoidal resonance structure, CSG gives its length to be about 0.01 Å longer compared to TG, which corresponds to the diradical resonance structure, in which that bond would appear to have a higher bond order. Even though these differences are quite small, their cumulative effect is quite significant, as demonstrated by changes in diradical character and singlet–triplet gap in Figure 5. Since CSG only allows closed-shell quinoidal VBF and TG only allows diradical VBF, these geometries define two extremes. The results using CSG are biased toward closed-shell structure and lower diradical character, while results from TG are biased toward higher diradical character.

### Critical Increase of the Diradical Character in PAH, PA, and PF

In order to increase the diradical character of this indolo[3,2-*b*]carbazole system, compounds with allenic substituents such as **PAH** and **PF** shown in Figure 4 and **PA** shown in Figure 6 were designed. In other diradical(oid) compounds in this work, two important resonance structures are closed-shell quinoidal and diradical aromatic (the latter being singlet or triplet). The idea is to “lock” the compound in a diradical resonance structure by means of breaking direct π-conjugation by connecting doubly bonded substituents to the CB ring. With these substituents, the π-conjugation between unpaired electrons is no longer direct; therefore, they cannot close this shell unless the π-bonds between the substituent and carbons of the CB ring are broken and two unpaired electrons are transferred to other groups (see Figure 6). Therefore, through this cross-conjugation between π-conjugated horizontal quinoidal chain and vertical chain with allenic substituents, we create the molecule of which minimal polyradical character is 2, that is, it is at least a diradical. Thus, these compounds would be expected to have very high diradical character, as verified from CASSCF calculations shown in Figure 5. We can make a reasonable judgment that thed biradical resonance structure on the left in Figure 6 is a more important contributor to the resonance hybrid than the biradical resonance structure in the middle due to the greater number of Clar’s π-sextets and the possibility of the delocalization of unpaired electrons into electron-withdrawing π-conjugated cyano groups, which is precluded in the middle resonance structure.

We can also predict that the contribution of a tetraradical resonance structure (on the right in Figure 6) in a resonance hybrid should be small, as it only has one more Clar’s π-sextets compared to the resonance structure on the left, and it has two more unpaired electrons, which cannot delocalize into the adjacent cyano groups. This is also verified by the results from CASSCF(14,14) calculations. First, a quintet spin state is much higher in energy than singlet and triplet states for CSG and TG, while even for CS geometry, singlet–triplet gaps are very small: 0.15 kcal/mol (52 cm^–1^), 0.30 kcal/mol (105 cm^–1^), and −0.60 kcal/mol (−210 cm^–1^) for **PAH**, **PA**, and **PF**, respectively. Second, as we refer to CASSCF frontier NOs (HONO and LUNO) from the singlet ground state in Figure 7, it is apparent that the resonance structure on the left is the most important contributor to the resonance hybrid as the predicted density distribution of the unpaired electrons by this structure matches that of HONO and LUNO. Hence, breaking the direct π-conjugation of the quinoidal structure and securing a diradical resonance structure increased the diradical character dramatically.

Even though the allenic derivatives of **PH**, **PAH**, **PA,** and **PF** could have been speculated to have some tetraradical character, CASSCF calculations showed them to be negligible. In addition, it is clear that terminal groups on these allenic substituents do not affect the diradical character significantly since **PAH**, **PA**, and **PF** have very high and very similar diradical characters. This means that the principal factor in increasing the diradical character in these compounds is the topology of the π-conjugation that restricts the lower bound of polyradical identity to diradical. It is exactly this idea that allows a leap from diradicals to tetraradicals.

### Leap from Diradicals to Tetraradicals

**Figure 9:**
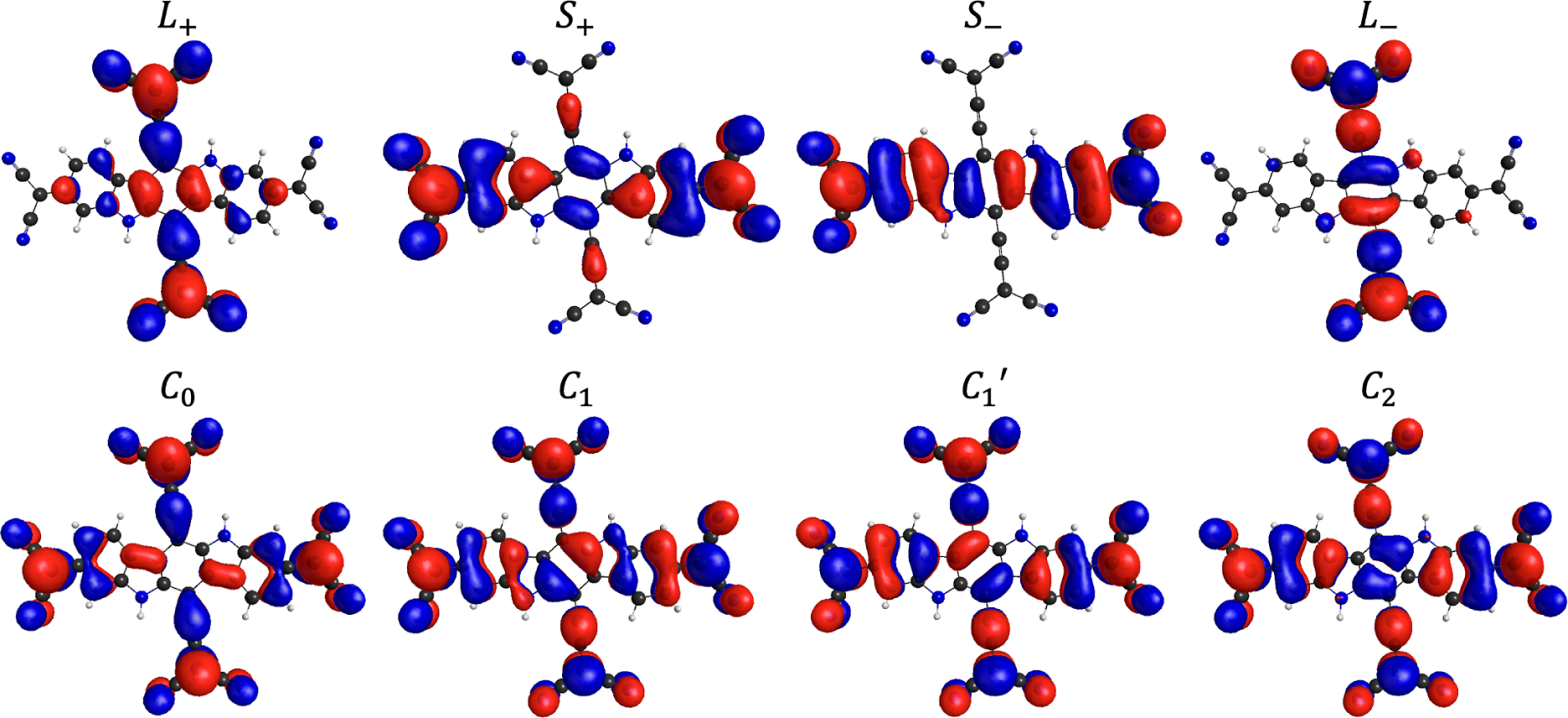
CASSCF frontier NOs of **PT** with symbolic assignations. Isosurfaces with the value of 0.015.

The molecule with only two more carbon atoms than **PA** (Figure 4) and fully planar *C*_2*h*_ geometry manifests a large diradical character and a substantial tetraradical character in its ground state. Resonance structures of this molecule (Figure 8) stress that **PT** must have an open-shell character in all cases since a closed-shell resonance structure/VBF cannot possibly be drawn for this molecule due to the cross-conjugated topology of its π-system. Thus, it must be at least a diradical (and could be a tetraradical). Frontier CASSCF NOs for **PT** are given in Figure 9, and corresponding CASSCF results are given in Table 1 for a quintet-optimized geometry. Symbolic assignment of the presented frontier NOs is based on the diradical components in our recent Letter^33^ (for orbitals L_±_ and S_±_) and analogy to cyclobutadiene (C) frontier orbitals with corresponding number of nodes for each orbital C_0–2_. For orbitals L_±_ and S_±_, the letter denotes which subunit bears the orbital mostly, and the sign shows whether the two radical centers have the same phase of the wave function or not. For orbitals C_0–2_, the letter emphasizes analogy to the frontier orbitals of the cyclobutadiene, and the index shows the number of nodes and corresponding energy ordering from lower energy of orbital with zero nodes to higher energy of orbital with two nodes. Since we are using the monoconfigurational method DFT to optimize the geometry of **PT**, the indicated spin multiplicity will define which VBFs are allowed, and this will translate into variance in optimized geometries. When we optimize geometry for a quintet state of **PT**, the resulting structure will resemble the VBF of the tetraradical (Figure 8) only, which means bias toward tetraradical states and bias against diradical states. However, when we optimize geometry for a triplet state, we allow all diradical and tetraradical VBFs to be mixed in the resulting structure, which means bias against tetraradical states and bias toward diradical states because there are much more diradical VBFs than tetraradical VBFs for **PT**.

**Table 1:** CASSCF(12,12) Results^b^ of **PT** with Energy Gaps from the Ground State (G. S.) in cm^–1^^a^^,^^c^

state	symmetry	HONO – 1		HONO		LUNO		LUNO + 1		Δ*E* from G. S.
S_0_	*A*_*g*_	L_+_	1.497	S_+_	1.122	S_–_	0.872	L_–_	0.511	0.00
T_0_	*B*_*u*_	L_+_	1.540	S_–_	1.000	S_+_	0.996	L_–_	0.465	431.97
T_1_	*B*_*u*_	S_+_	1.154	L_+_	1.033	L_–_	0.957	S_–_	0.858	1702.61
S_1_	*A*_*g*_	C_0_	1.140	C_1_	1.001	C_1_^′^	0.963	C_2_	0.897	1940.07
T_2_	*A*_*g*_	C_0_	1.106	C_1_	1.054	C_1_^′^	0.943	C_2_	0.898	1952.71
Q_0_	*A*_*g*_	C_0_	1.005	L_–_	1.000	C_2_	0.999	S_–_	0.997	2177.38

a Columns HONO, LUNO, etc. show CASSCF NOs identities from Figure 9 and their occupation numbers.

b See Tables S2 and S3 in the Supporting Information for detailed CASSCF(4,4) and CASSCF(12,12) data, respectively.

c **L** and **S** are diradical components from our recent Letter,^33^ while C is the analogy to cyclobutadiene frontier orbitals.

These geometries define two extremes for all spin states in the low-energy spectrum of **PT**.

A thorough geometrical characterization of **PT** with optimized geometries of different spin states is given in Figure S10 in the Supporting Information. A quintet-optimized geometry has slightly longer bonds, where they are expected from resonance structures. Comparison of CASSCF and CASCI results for triplet-optimized and quintet-optimized geometries are given in Tables S9–S16 in the Supporting Information. For a triplet-optimized geometry, CASSCF(12,12) results indicate diradical character *y*_0_ = 0.80, tetraradical character *y*_1_ = 0.016, the energy gap between T_0_ and T_1_ states  4738 cm^–1^, and the spectral range of six low-energy spin states to be 5669 cm^–1^. For a quintet-optimized geometry, CASSCF(12,12) results show diradical character *y*_0_ = 0.75, tetraradical character *y*_1_ = 0.21,  1271 cm^–1^, and spectral range of six low-energy spin states to be 2177 cm^–1^. Hence, **PT** can be claimed to be a tetraradicaloid with a small tetraradical character (between 0.016 and 0.21). Also, based on HONO and LUNO of the S_0_ state, unpaired electrons are mostly localized on the methylene carbons (and on the atoms in successively allylic positions from them) of dicyanomethylene substituents at the ends of terminal benzenoid rings of the indolo[3,2-*b*]carbazole backbone. Thus, it is evident from the visual appearance and occupation numbers of NOs of the S_0_ state that it mostly resembles the resonance structure on the rightmost position of Figure 8. The better stabilization of tetraradical electronic structure in **PT** than in **PAH**, **PA**, and **PF** is provided by the possibility of each of the additional two unpaired electrons to delocalize into the two terminal cyano groups, which cannot happen in the case of any such allenic derivative because the π-system of the terminal groups is essentially orthogonal to the π-system, where two additional unpaired electrons lie. In total, the greater electron delocalization in the case of planar **PT** than allenic molecules with more restricted delocalization enhances the tetraradical character of **PT**.

**Figure 10:**
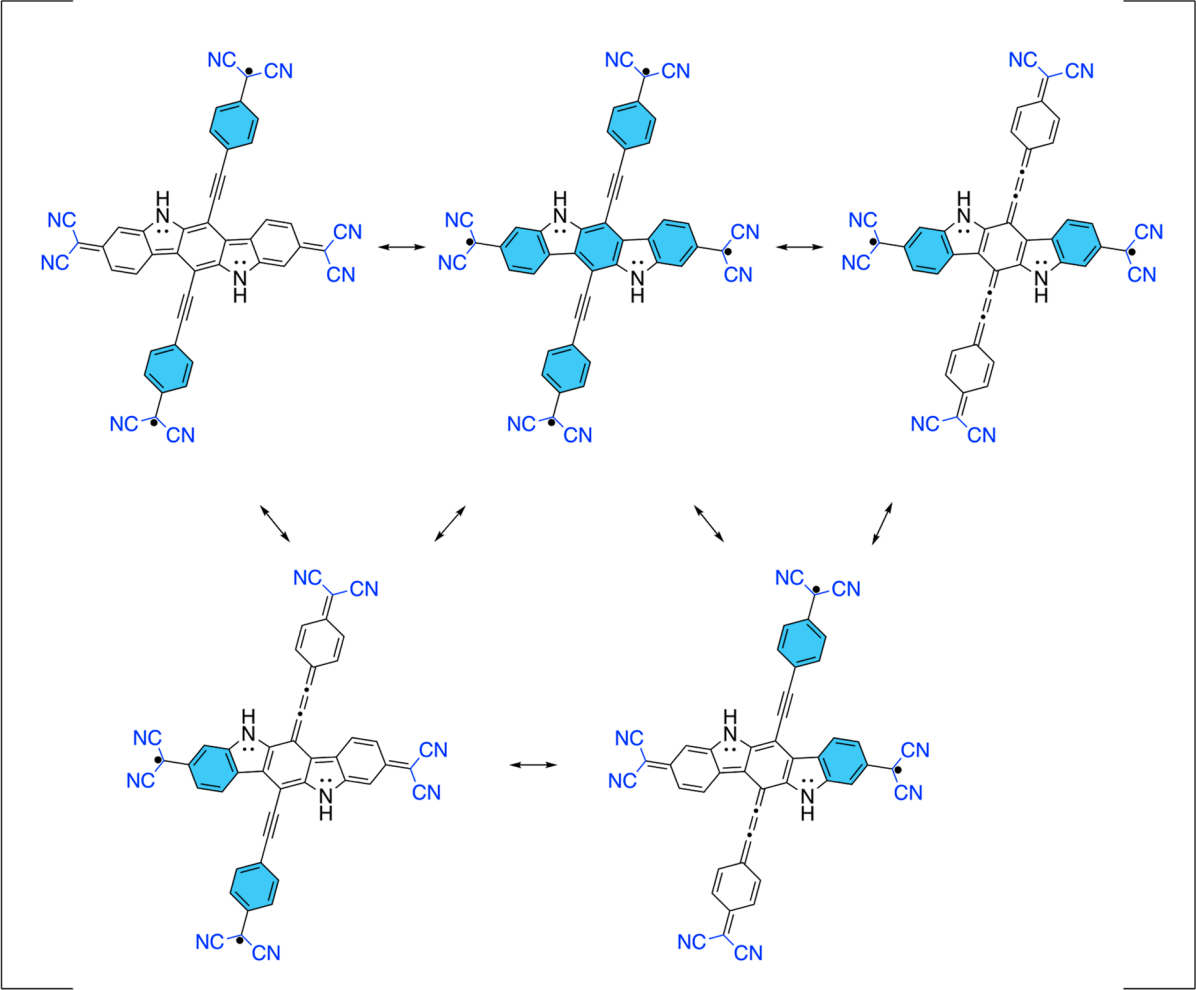
Resonance structures of **PTP**.

By insertion of benzene rings between dicyanomethylene and ethynyl residues of both 3-dicyanomethylene-ethynyl substituents, we obtained **PTP** (Figure 10). For all three geometries, CASSCF results are qualitatively the same, differing by small amounts mostly in energetics and very small amounts in CASSCF NOs occupancies. A comparison between CSG, TG, and QG optimized geometries is shown in Figure S11 of the Supporting Information. Frontier CASSCF NOs for **PTP** are given in Figure 3, and corresponding CASSCF results are given in Table 1 in our recent Letter,^33^ which characterized the electronic structure of the tetraradical(oid) **PTP** (in previous work, referred to as **LS**) and verified its tetraradical(oid) electronic structure. S_0_ state shows almost single occupation numbers of HONO – 1 and LUNO + 1, which means this molecule pronounces a high tetraradical character. Indeed, diradical (*y*_0_) and tetraradical (*y*_1_) indices are 0.91 and 0.58, respectively.^33^ It is noteworthy that energy gaps between different spin states are diminished dramatically for **PTP** compared to **PT** and studied diradical(oid)s. All spin states are within 603 cm^–1^ (1.72 kcal/mol),^33^ making them thermally accessible.

Two additional benzenoid rings contribute aromatic resonance energy to the stabilization of the tetraradical(oid). The tetraradical VBF of **PTP** has three more Clar’s π-sextets compared to diradical VBFs, as shown in Figure 10.

It is also noteworthy that for the molecule related to this, but without additional benzenoid rings inserted between dicyanomethylene and ethynyl residues, **PT** has some tetraradical character (*y*_1_ = 0.016–0.21) but significantly less than **PTP**. It must be emphasized that **PTP** satisfies every requirement in the guidelines given at the end of the introduction, while **PT** does not satisfy requirement (2) since one of its diradical subunits has aromatic stabilization from only one Clar’s π-sextet.

**Figure 11:**
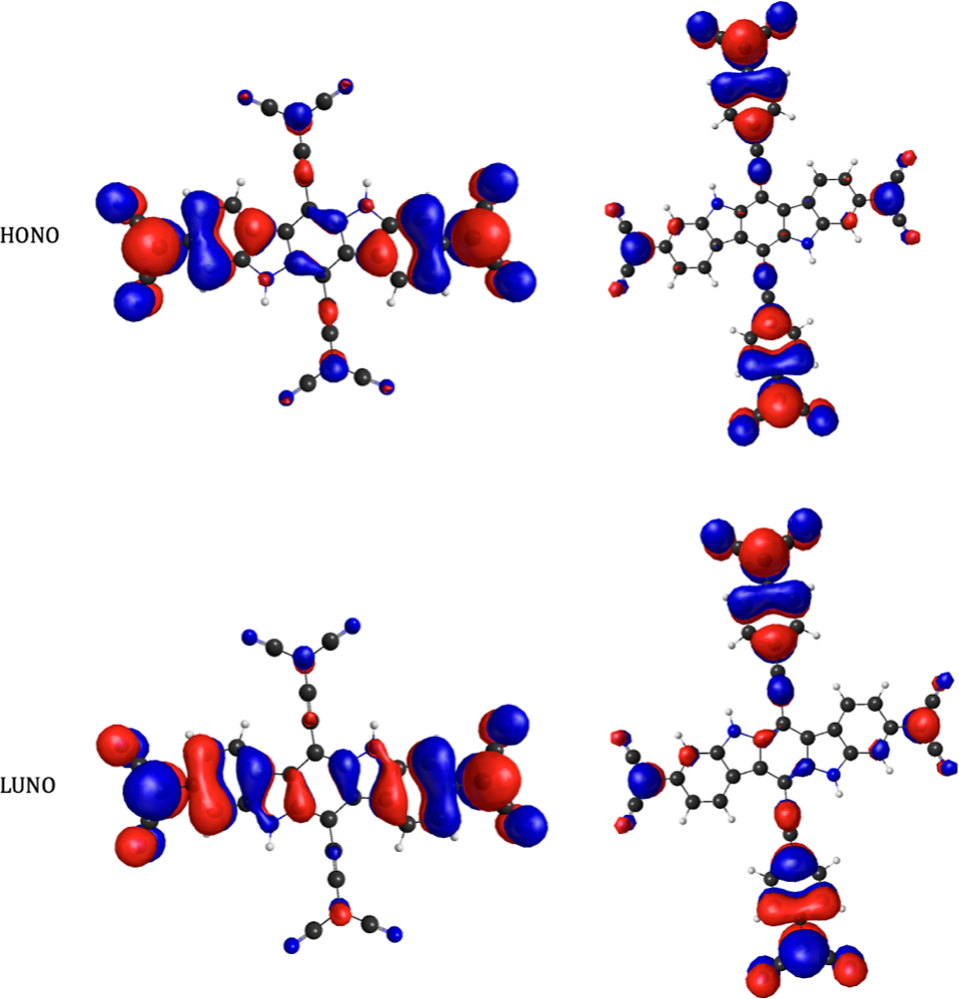
CASSCF frontier (HONO and LUNO) NOs of **PT** and **PTP**. Isosurfaces with a value of 0.015.

If we consider a “paradigm” of diradicals, we find that the insertion of two benzene rings between ethynyl and dicyanomethylene residues of the 2-dicyanomethylene-ethynyl substituent of **PT** shifts the position of the highest spin density due to unpaired electrons in other locations (other carbons and groups) and the other (perpendicular) direction. For illustration, it is sufficient to show the CASSCF frontier (HONO and LUNO) NOs of S_0_ states of **PT** and **PTP** side by side, as depicted in Figure 11.

### Rationalization of the Stability of Open-Shell Derivatives of PH

**Figure 12:**
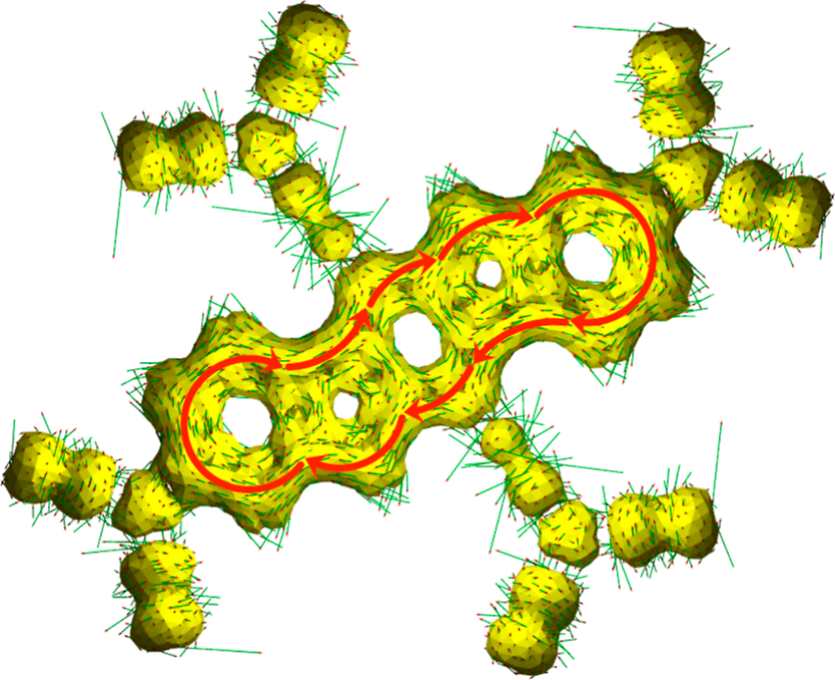
ACID plot for a singlet state of **PT** calculated with the B3LYP^108−111^ functional and the cc-pVDZ basis set. Isosurface with the value of 0.030.

To explain the reason for the relative quintet state stability of **PT** and **PTP**, we consider the role of aromaticity as a stabilizing factor. For **PT**, a tetraradical resonance structure allows three Clar’s π-sextets, while diradical resonance structures allow only two Clar’s π-sextets or even none of the Clar’s π-sextets. Notably, one more Clar’s π-sextets for tetraradical resonance structures as opposed to diradical resonance structures is not usually enough to explain a significant tetraradical character^30^ (analogically to pQDM shown in Figure 1a). It is therefore important to explore if additional aromatic stabilization can be gained by “global aromaticity” (see ref (25)). ACID is a method that can give us some insight into the role of “global aromaticity”, as it describes the response of electron currents on a perpendicular magnetic field, leading to a flow of electron density around the aromatic rings. Since electron delocalization is one of the main features of aromaticity, results from ACID can tell us whether the electron flow is continuous throughout the molecule (“global aromaticity”) or is more restricted to the subsystems of the molecule (local aromaticity). These results allow the analysis of the role of aromaticity in the electronic structures of different spin states. For the selected derivatives of **PH**, ACID plots for all relevant spin states are given in Figure S12 in the Supporting Information and they unequivocally indicate delocalization around the indolo[3,2-*b*] backbone of these compounds (except **PAH**, **PA**, and **PF**), making them globally aromatic. Figure 12 shows an ACID plot for a singlet state of **PT** using a quintet-optimized geometry. It is apparent that the continuous electron flow around the perimeter of indolo[3,2-*b*]carbazole is established. ACID plots for triplet (Figure S12n) and quintet (Figure S12o) states are given in the Supporting Information, and they are almost indistinguishable from the singlet state and one another in the aspect of electron delocalization.

Assuming that the “global aromaticity” of this indolo[3,2-*b*]carbazole system is a greater stabilizing factor than the local aromaticity of separated Clar’s π-sextets, based on these data, a significant delocalization of electrons relatively stabilizes otherwise unstable **PT** tetraradical(oid) structure in both triplet-optimized or quintet-optimized geometries. The concept of “global aromaticity” must be invoked to explain the relative stabilization of tetraradical states of **PT** because the argument of one more Clar’s π-sextet is usually insufficient, as the examples of pQDM in Figure 1a, Thiele’s hydrocarbon, and many other compounds show.^30^ To justify the stability of the tetraradical(oid) electronic structure of **PTP**, invoking the idea of Clar’s π-sextets is much more convincing and sufficient than for **PT**. Putting aside “global aromaticity”, which **PTP** also possesses for every relevant spin state,^33^ by shifting from the diradical to the tetraradical resonance structure (Figure 10), **PTP** gains three additional π-sextets and stabilizes its tetraradical (singlet, triplet, and quintet) states. This is also corroborated by the calculations of the MCI of aromaticity given in Table S8 and Figure S14 of the Supporting Information, which indicate that the benzenoid rings in the presented compounds are aromatic. We must note that the quintet spin state allows only one VBF in the electronic structure, which is a tetraradical structure that has three more Clar’s π-sextets than diradical structures. In the case of singlet and triplet spin states, the electronic configuration is a mixture of all of the diradical and tetraradical structures (VBFs). Some benzenoid rings in these diradical structures are in quinoidal form, and they can also prevent “global aromaticity”. This mixing of configurations lowers the average aromaticity of the rings and “global aromaticity” in singlet and triplet spin states. This is also corroborated by MCI results for both **PT** and **PTP**, as the MCI of aromaticity for six-membered rings in the quintet state is higher than in singlet and triplet states, as shown in Table S8 and Figure S14 in the Supporting Information. Thus, it is expected that the quintet spin state will be stabilized more by the presence of local and “global aromaticity” than the singlet and triplet states.

## Conclusions

In this work, we studied a series of diradical(oid)s based on a common parent compound 2,2’-(5,11-dihydroindolo[3,2-*b*]carbazole-3,9-diyl)dimalononitrile (**PH**). By connecting the different substituents to its CB ring, the diradical character was significantly modulated. From these results and rationalizations, we can clearly state that to control the diradical (and generally, polyradical) character, one must control the topology of the π-conjugation. It has been established clearly on the examples of allenic, dicyano-allenic, and difluoro-allenic derivatives, **PAH**, **PA**, and **PF**, that by breaking the direct π-conjugation between unpaired electrons that give rise to radical centers and effectively “locking” the system in its diradical VBF so that it cannot close the shell, the diradical character increases dramatically. Moreover, it is clear that relatively minor differences in geometry sometimes translate into significant differences in the diradical character and singlet–triplet gap.

It is important to stress that for the diradicals based on indolo[3,2-*b*]carbazole backbone, singlet–triplet gap and diradical character do not strictly correlate. Some of them have larger singlet–triplet gaps than others but still have a greater diradical character. Hence, it has been established that despite being Kekulé diradicals and possessing direct π-conjugation (which allows close-shell quinoidal and diradical aromatic VBFs), the connection between the singlet–triplet gap and diradical character for derivatives of 2,2’-(5,11-dihydroindolo[3,2-*b*]carbazole-3,9-diyl)dimalononitrile does not follow the relation already established for Thiele-like compounds^105^ and the seemingly similar sets of compounds in terms of the nature of π-conjugation.^4,5,29,32,64,102−105^

**Figure 13:**
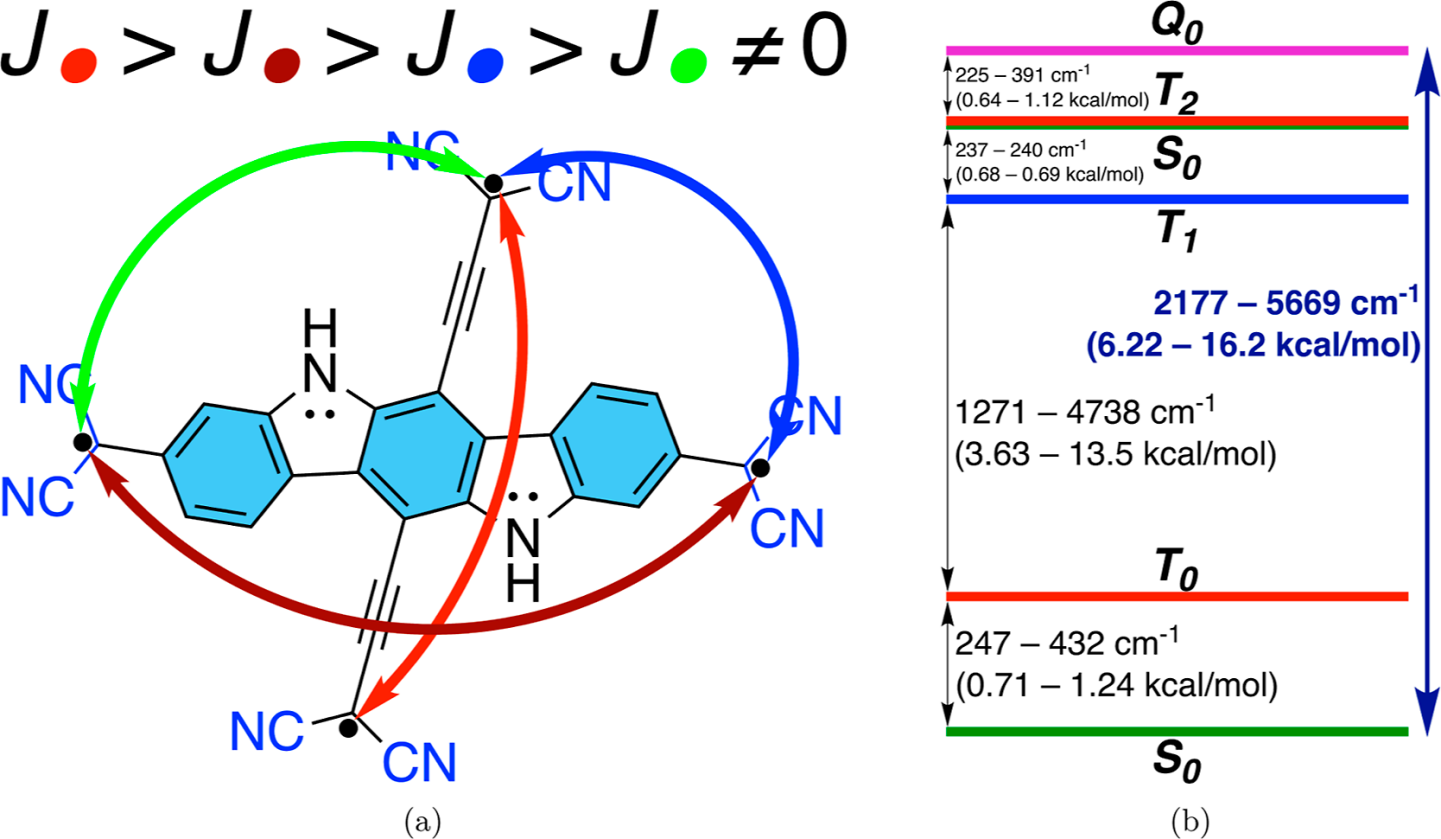
(a) Scheme of exchange-coupling constants and their strengths and (b) energy spectrum of low-lying spin states of **PT**.

From the idea of designing allenic derivatives **PAH**, **PA**, and **PF**, the tetraradical(oid) **PT** was designed by realizing that it has a persistent open-shell character and is at least a persistent diradical due to cross-conjugated structure (i.e., mutual exclusion in closing the shell via direct π-conjugation for different pairs of unpaired electrons). The unexpected result could have been that **PT** possesses a substantial tetraradical character (especially when we use quintet-optimized geometry), but it appears that “global aromaticity” stabilization and capacity of delocalization of unpaired electrons into adjacent cyano groups contrary to **PAH**, **PA**, and **PF** could at least partially explain the reason behind this. A scheme of the exchange-coupling constants between radical centers with their relative strengths and energy spectra of low-lying spin states is given in Figure 13. The ordering of the strengths of coupling can be explained by the presence or absence of direct π-conjugation and the through-bond distance between two radical centers. The radical centers that are conjugated directly (i.e., in some VBFs there is a continuous chain of alternating π bonds) have stronger coupling than those that do not (see Figure 8). Among those directly π-conjugated pairs, the shorter through-bond distance between radical centers usually means stronger coupling, which is indeed manifested for **PT** and **PTP**. We must keep in mind that this scheme in Figure 13a describes only the tetraradical contribution (up to 21%) to the more convoluted electronic structure of **PT**. By inserting two phenyl rings in 2-dicyanomethylene-ethynyl substituents, the tetraradical character was increased dramatically in **PTP**, with all of the spin states in the low-energy spectrum within 603 cm^–1^ (1.72 kcal/mol). Consequently, we provided a start of a pathway from diradicals to higher-order polyradicals and showed factors that contribute to the modulation of diradical/tetraradical characters, singlet–triplet/singlet-quintet gaps, and spectral range of the low-lying spin states. This work bridges the gap between diradicals, which are explored extensively, to tetraradicals and higher-order polyradicals, which are at the frontiers of molecular magnetic systems. The idea of restricting lower-bound polyradical character to at least diradical by means of cross-conjugation and increasing open-shell stabilization by aromaticity of the bridging groups can be extended to design higher-order polyradicals and is being further explored intensively by our group.

## Data Availability

The data underlying this study are available in the published article and its Supporting Information.

## Supporting Information Available

General view of the diradical(oid) and tetraradical(oid) derivatives, computational details of geometry optimization, methodology of the open-shell character test, computational details of CASSCF and CASCI, details of the build of model and effective Hamiltonian, calculation of exchange-coupling constants, results of DFT benchmark with different exchange–correlation functional for different states of **PH**, results of restricted and unrestricted Hartree–Fock calculations for diradical(oid) derivatives of **PH**, results of CASSCF and CASCI calculations for triplet- and quintet-optimized DFT geometries of **PT**, geometry comparisons between CSG and TG (and QG) of **PH**, **PT** and **PTP**, results from ACID and MCI calculations, summary of the electronic structure of **PT**, and optimized geometries for every relevant state of all the compounds presented in this study (PDF)
