# Supplementary material for: Leap from Diradicals to Tetraradicals by Topological Control of π-Conjugation
Source: J Org Chem. 2024 Sep 20;89(19):14006–20. doi: 10.1021/acs.joc.4c01375 (PMC11460765; doi:10.1021/acs.joc.4c01375)
Supplement: Supplementary file 1 — jo4c01375_si_001.pdf [file jo4c01375_si_001.pdf]

Supporting Information

A Leap from Diradicals to Tetraradicals by Topological Control of π-Conjugation

Sergi Betkhoshvili,[†] Jordi Poater,[*,†,¶] Ibério de P. R. Moreira,[*,‡] and Josep Maria Bofill[*,†]

† *Departament de Química Inorgànica i Orgànica and Institut de Química Tèorica i Computacional (IQTCUB), Universitat de Barcelona, Martí i Franquès 1–11, 08028 Barcelona, Spain.* E-mail: jmbofill@ub.edu

‡ *Departament de Ciència de Materials i Química Física and Institut de Química Tèorica i Computacional (IQTCUB), Universitat de Barcelona, Martí i Franquès 1–11, 08028 Barcelona, Spain.* E-mail: i.moreira@ub.edu

¶ *ICREA, Pg. Lluís Companys 23, 08010 Barcelona, Spain.* E-mail: jordi.poater@ub.edu

Contents

S1. General View of the Investigated Diradical(oid)s and Tetraradical(oid)s **S1**

S2. Computational Details **S3**
- S2.1. Geometry Optimizations . . . S3
- S2.2. Detailed Description of *ab-initio* Calculations . . . S3

S3. Data Obtained in the Calculations **S5**
- S3.1. DFT Benchmark and Hartree-Fock Results . . . S5
- S3.2. CASSCF and CASCI Results for Diradicals and Tetraradical(oid) PT . . . S6
- S3.3. Calculations of Exchange-Coupling Constants Between Radical Centers . . . S9

S4. Geometry Comparison for Diradical(oid)s and Tetraradical(oid)s **S11**

S5. Study of *local* and *global* Aromaticity of Diradical(oid)s and Tetraradical(oid)s **S13**

S6. Summary of the Electronic Structure of Tetraradical(oid) PT **S18**

S7. Comparison of CASSCF and CASCI Results for Triplet- and Quintet-optimized Geometries of PT **S18**

S8. Optimized Geometries **S20**

S1. General View of the Investigated Diradical(oid)s and Tetraradical(oid)s

The compounds studied in this work share a common parent compound 2,2′-(5,11-dihydroindolo[3,2-*b*]-3,9-diyl)-dimalononitrile (**PH**) shown in Figure S2. This compound was computed and experimentally verified[1,2] to have a moderate diradical character. It possesses "*global aromaticity*" in its indolo[3,2-*b*]carbazole backbone and (counted separately) it has three Clar's aromatic π-sextets.[3] All the derivatives studied within this work (except **PAH**, **PA** and **PF**) share these properties with the parent compound **PH**. Upon substituting *para* hydrogen atoms of the central benzenoid ring in **PH**, the lateral conjugation is juxtaposed and complementary to the conjugation between substituents in relative *para* positions in the perpendicular direction. The substituents, through which different derivatives of **PH** are designed, vary in the length of the π-conjugated chain, electron-withdrawing and electron-donating properties. These derivatives also vary in their dipole moment depending on whether the two

substituents in the central benzenoid ring are the same or not. This co-existence of conjugation in perpendicular directions distorts the electron density in the central benzenoid ring of derivatives of **PH**, making the central benzenoid ring less aromatic than terminal benzenoid rings, as demonstrated in Figure S14.

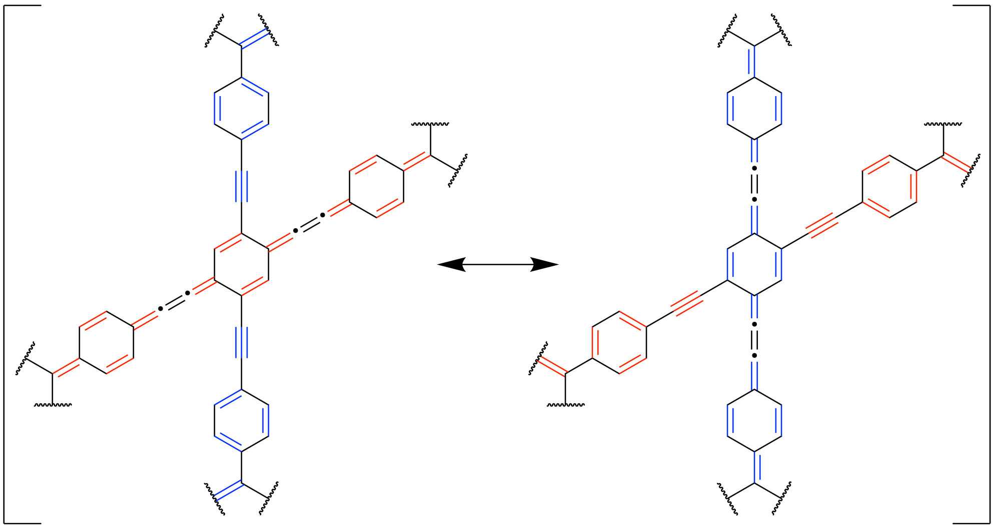

Figure S1. Illustration of cross-conjugation with an organic framework. π-systems crossing each other in the central benzenoid ring are shown in red and blue.

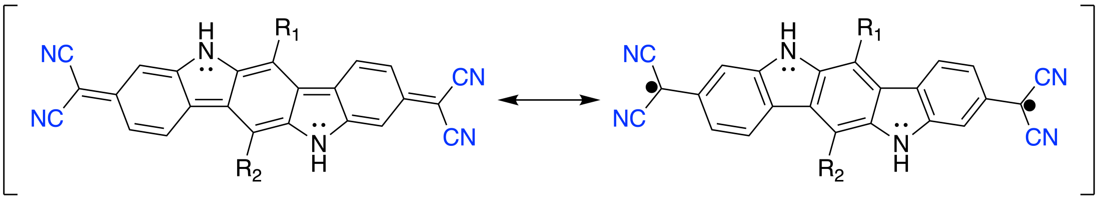

(a) The generic drawing of closed-shell (quinoidal) and open-shell (biradical) resonance structures of derivatives of the parent compound **PH**.

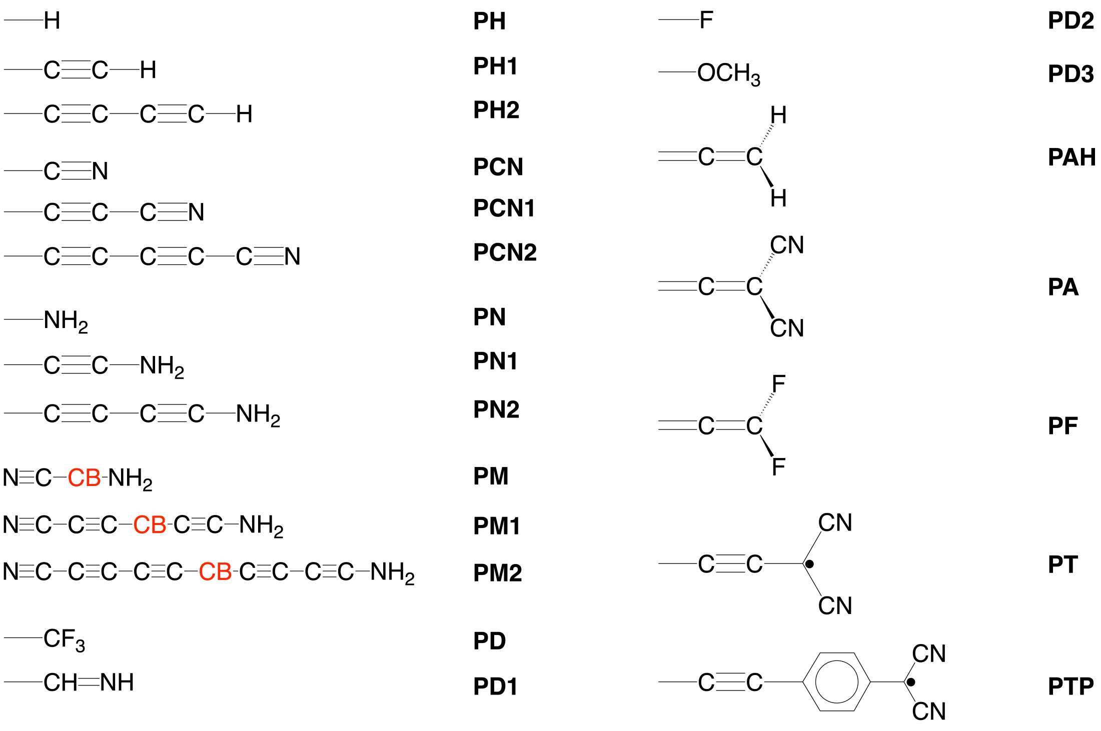

(b) Chemical depictions of substituents and corresponding abbreviations.

Figure S2. A set of molecules that have been studied in this work with their abbreviations.

In compounds **PAH**, **PA** and **PF**, the direct linear π-conjugation is broken by allenic substituents doubly bonded to the central benzenoid ring of the indolo[3,2-*b*]carbazole moiety. This prohibits the closed-shell structure of these compounds by topological control of π-conjugation. Moreover, in **PAH**, **PA** and **PF**, the central benzenoid ring is no longer aromatic, as opposed to all the other diradical(oid) and tetraradical(oid) derivatives of **PH**. The restriction of the lower bound of polyradical character to 2 has been extended to design cross-conjugated tetraradical(oid)s **PT** and **PTP** (latter already described in our recent Letter[4]), which cannot assume a closed-shell electronic structure: they are *at least* diradicals and possess significant tetraradical(oid) character. We define *cross-conjugation* as follows: if in some valence bond forms (VBFs, i.e. Lewis structures) two terminal sites of the molecule are continuously connected by alternating π bonds, this would forbid a continuous connection via alternating π bonds for *at least* one different pair of terminal sites. Since such conjugated π chains intersect or *cross* each other, we call such systems cross-conjugated. For illustration, we can refer to Figure S1, which includes a generic (closed-shell) cross-conjugated organic framework, where the π-systems intersect/cross each other in the central benzenoid ring.

S2. Computational Details

S2.1. Geometry Optimizations

For geometry optimizations of the molecules, Density Functional Theory (DFT) was used with Amsterdam Density Functional (ADF) software code[5] within Amsterdam Modeling Suite (AMS) release of 2023 (ADF 2023.104 version). The self-consistent field (SCF) convergence criteria: the norm of the commutator between Fock and Density matrices ([F,P]) $\leq 1.0 \cdot 10^{-5}$ and a maximum element of the [F,P] $\leq 1.0 \cdot 10^{-6}$ (defaults). Numerical quality for Becke's integration grid:[6,7] "VeryGood", i.e. Lebedev angular grid order in the range [13, 41] and the number of radial points in the range [64, 72]. Numerical quality for density fitting approximation: "VeryGood", meaning a number of interpolation points in the range [120,160] and a maximum number of L-expansion in the range [9,10]. Since geometry can be an important variable in determining the electronic structure (this is the reflection of the existence of Potential Energy Surface (PES)) of molecules, in this work, identical calculations were done on both: closed-shell singlet (CSS) optimized geometry as a result of restricted Kohn-Sham (RKS) DFT calculations and triplet-optimized geometry as a result of unrestricted Kohn-Sham (UKS) triplet DFT.[8–10] Slater type orbitals triple-ζ basis set (TZP)[11] was used for all ADF/DFT calculations throughout this work. Furthermore, vibrational frequencies were computed for each optimized geometry in order to verify whether or not they correspond to the local minimum on PES.[12–14] From now on, the closed-shell singlet-optimized geometry will be referred to as CSG and triplet-optimized geometry will be referred to as TG. In some cases, it was necessary to optimize geometry for a quintet spin state, which will be referred to as QG. Except for benchmark calculations of different states of **PH** with different exchange-correlation functionals, for all DFT calculations (geometry optimizations, Hessian calculations, etc.) Generalized Gradient Approximation (GGA) exchange-correlation functional BLYP[15,16] was used. It must be noted explicitly that studying the electronic structures of biradical(oid)s, which are intrinsically multiconfigurational, is unreliable with DFT. Empirical proof of this claim on the parent compound **PH** was demonstrated by the benchmark with different exchange-correlation functionals. It is evident that energy ordering for closed-shell singlet, open-shell singlet and triplet depends very sensitively on the exchange-correlation functional used for DFT calculations. Nevertheless, this dependence is important only for energies of optimized structures, while geometries themselves obtained by optimization for corresponding spin states with different functionals are very similar. Still, as discussed in the manuscript, these minor differences might sometimes translate into the significant modulation of singlet-triplet gaps, spectral ranges of low-lying spin states and diradical/tetraradical character indices (see definitions in the next subsection).

S2.2. Detailed Description of *ab-initio* Calculations

Hartree-Fock method with its restricted and unrestricted formalism (RHF and UHF, respectively) is a computationally cheap tool to evaluate whether or not the molecule has an open-shell electronic structure. To achieve this, we need to compute RHF and UHF solutions separately and compare them. If the UHF solution has lower energy than the RHF solution, it means that the closed-shell electronic wave function is unstable and the ground-state wave function is stable with an open-shell, UHF solution. For most Hartree-Fock calculations, Gaussian 2016 software was used.[17] The basis set used for all calculations except geometry optimizations was Dunning's correlation-consistent double-ζ basis set cc-pVDZ.[18] To study the multiconfigurational wave function of diradical(oid)s and tetraradical(oid)s presented in this work, a multireference method such as Complete Active Space Self-Consistent Field (CASSCF), which is also known as Full Optimized Reaction Space (FORS), was used.[19–25] CASSCF calculations were performed using the General Atomic and Molecular Electronic Structure System (GAMESS).[26] In most cases, the initial guess orbitals for CASSCF calculations were UHF natural or-

bitals (hence, some HF calculations were performed in GAMESS) as they are one of the best starting orbitals for CASSCF.[27–29] For CASSCF calculations, like for Hartree-Fock calculations, cc-pVDZ basis set was used. Natural orbitals (NOs) are eigenvectors of the first-order density matrix operator, and their corresponding eigenvalues are natural orbital occupation numbers (n_{NO}).[30] For diradical/diradicaloid molecules, in most cases, 14 electrons in 14 orbitals CASSCF calculations, CASSCF(14,14), were enough based on the occupation numbers of UHF natural orbitals.[27,29] For some instances, restricted open-shell Hartree-Fock (ROHF) optimized orbitals (these calculations were done in GAMESS as well) were used as initial guess for CASSCF calculations to troubleshoot convergence problems for different spin states. For tetraradical(oid)s, CASSCF(12,12) and CASSCF(16,16) calculations were performed to allow capturing of so-called nondynamic correlation.

To explore the wave function of these open-shell systems in greater detail, CAS Configuration Interaction (CASCI) calculations were performed with (4,4) and (12,12) active spaces for tetraradical(oid) **PT**, and with (14,14) or (16,16) active spaces for diradical(oid)s. We conducted CASCI calculations with the following procedure: (1) We perform a UHF calculation for the state of the highest multiplicity for a compound (triplet for diradical(oid)s and quintet for tetraradical(oid)s). (2) Starting from the set of UHF natural orbitals as an initial guess, we perform CASSCF(12,12), (14,14) or (16,16) calculation (depending on the compound) of the state of the highest multiplicity and localize only the singly occupied molecular orbitals (SOMOs), which are either two or four, depending on whether we are dealing with a diradical(oid) or a tetraradical(oid), respectively. (3) We use converged CASSCF orbitals, which are localized in the minimal subspace of SOMOs, and then initiate CASCI calculation with determinants of $S_z = 0$ subspace to obtain all the relevant spin states. Since we are using the localized guess, the coefficients of contributing determinants of the wave function translate into the distribution of the electron density of SOMOs. Such localized orbitals for tetraradical(oid) **PT** are given in Figure S3.

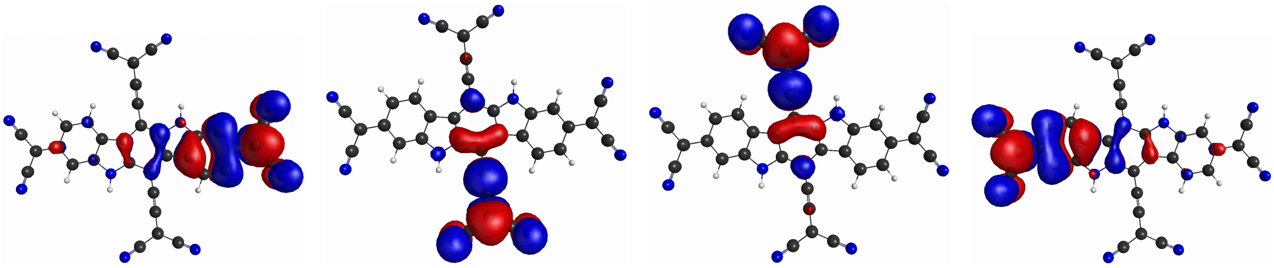

(a) SOMO 1 **(b)** SOMO 2 **(c)** SOMO 3 **(d)** SOMO 4

Figure S3. Localized singly occupied molecular orbitals (SOMOs) of **PT** from the converged solution of CASSCF(12,12) quintet obtained from starting with converged UHF quintet NOs guess.

To demonstrate the tetraradical(oid) electronic structure for one of the compounds with monoconfigurational methods, we can calculate the UHF broken-symmetry singlet (to obtain tetraradical(oid) electronic structure solution) and examine UHF natural orbitals and their occupation numbers. In order to achieve this, we take converged CASSCF(12,12) quintet orbitals, with localized (4,4) minimal subspace of four SOMOs that define tetraradical(oid) electronic structure. We reorder sets of α and β orbitals in a way to obtain the spin arrangement of electrons in four orbitals so that they correspond to the determinant $|\alpha\beta\alpha\beta\rangle$ by spin configuration. With this reordered guess, we initiate a UHF singlet calculation. Frontier UHF natural orbitals of the converged solution of UHF singlet calculation are given in Figure S4.

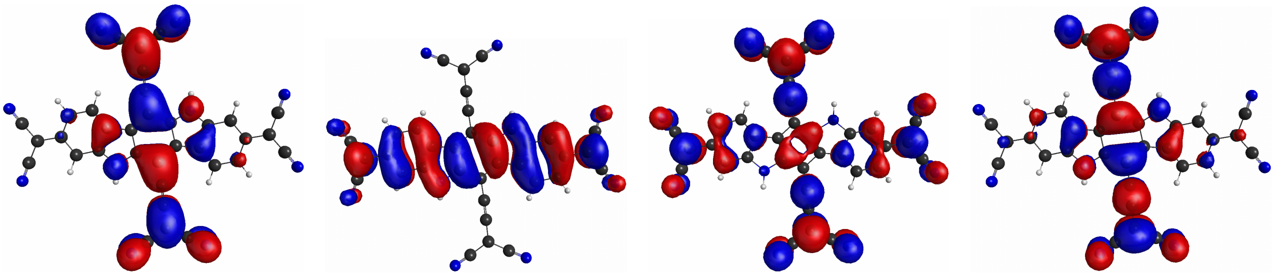

(a) $n_{HONO-1} = 1.458$ **(b)** $n_{HONO} = 1.000$ **(c)** $n_{LUNO} = 1.000$ **(d)** $n_{LUNO+1} = 0.542$

Figure S4. Frontier UHF natural orbitals and occupation numbers of broken-symmetry UHF singlet calculation for **PT** from the converged solution of CASSCF(12,12) quintet with localized SOMOs.

Furthermore, we used CASCI results for the characterization of interactions between radical centers. We built the Heisenberg-Dirac-van Vleck model Hamiltonian $\hat{H}_{HDvV}$[31–34] for diradical(oid)s and tetraradical(oid)s and compared them with corresponding effective Hamiltonians $\hat{H}_{eff}$[35,36] to calculate exchange-coupling constants

(J_{ij}) between radical centers. A more detailed explanation of the theoretical background, its application and the calculated results are given in Section S3.3.

For a description of "*global aromaticity*", the Anisotropy of the Induced Current Density (ACID) method was used.[37,38] ACID describes the response of electron currents on a perpendicular magnetic field, which leads to the flow of electron current around the aromatic rings. Hence, using ACID results, we can gauge the degree of electron delocalization, which is one of the most important features of aromaticity. The converged spin states for which ACID calculations were performed were obtained with Gaussian 16 program,[17] using unrestricted Kohn-Sham (UKS) DFT with B3LYP[39–42] exchange-correlation functional and cc-pVDZ basis set. For triplet states of diradical(oid)s, obtaining the qualitatively correct solution from a UKS calculation has been trivial. For open-shell singlet states of diradical(oid)s, in order to obtain a qualitatively correct solution, we initiate the calculation by mixing the highest occupied molecular orbital (HOMO) and the lowest unoccupied molecular orbital (LUMO), by keyword-option pair in Gaussian 16: "guess=mix". If the occupation numbers of frontier natural orbitals resulted in values close to 1, that is, we almost had two singly occupied natural orbitals (SONOs), we verify that the converged solution was qualitatively correspondent to diradical(oid) electronic structure. If by mixing HOMO and LUMO, still the closed-shell solution was obtained, we used the following procedure: (1) Obtain UKS triplet converged canonical orbitals for an initial guess. (2) Mix HOMO and LUMO of the triplet UKS initial guess to obtain a broken-symmetry singlet solution of UKS, which is verified to have qualitatively correct structure for diradical(oid)s. For tetraradical(oid)s, obtaining the qualitatively correct quintet state is trivial. For a singlet tetraradical(oid) state, firstly, we obtain the converged UKS canonical orbitals from quintet calculations, then mix HOMO and LUMO of UKS quintet guess, and initiate the calculation to obtain broken-symmetry singlet solution for the tetraradical(oid) with nearly four SONOs as shown in Figure S4. For a triplet tetraradical(oid) state, we take canonical UKS orbitals from broken-symmetry singlet solution for the tetraradical(oid), mix again HOMO and LUMO, and initiate the calculation, thus obtaining nearly four SONOs for the triplet state. When using a monoconfigurational method such as DFT for multiconfigurational systems for the determination of molecular properties, we must always make sure that the monoconfigurational method reproduces qualitative electronic structure from the multiconfigurational calculation properly. The verification that solutions contain nearly SONOs is one of the best markers for the correct qualitative behavior of the applied monoconfigurational method. With Hartree-Fock or DFT, we can capture states of different multiplicities, but usually not several states of the same multiplicity. Multiconfigurational methods such as CASSCF and CASCI allow this because these states merely correspond to the higher eigenvector roots of the CASSCF and CASCI Hamiltonian matrices. For the characterization of *local* aromaticity of the benzenoid rings in diradical(oid)s and tetraradical(oid)s, we use multicenter index (MCI) of aromaticity.[43,44] These values describe the degree of electron-sharing between atoms in the rings as a measure of aromaticity.

For calculations of diradical and tetraradical character indices, Yamaguchi's approach was used.[45] The n-radical character y_n varies from $y_n = 0$ meaning no n-radical character to $y_n = 1$ meaning full n-radical character. The highest occupied natural orbital (HONO) is defined as the orbital that has the lowest occupation number n_{NO} among natural orbitals (NOs) with $n_{NO} \geq 1$. The lowest unoccupied natural orbital (LUNO) is defined as the orbital that has the highest n_{NO} among NOs with $n_{NO} \leq 1$. In UHF, $n_{HONO-i} + n_{LUNO+i} = 2.000$, which is usually also manifested in CASSCF and CASCI natural orbitals. $2(i+1)$-ple radical character is calculated as follows:

$$T_i = \frac{n_{HONO-i} - n_{LUNO+i}}{2} \rightarrow y_i = 1 - \frac{2T_i}{1 + {T_i}^2}$$

By substituting $i = 0$, we obtain y_0, and $2(0+1)$-ple radical character. Hence, y_0 is a diradical character index. Analogically, by substituting $i = 1$, we obtain y_1, which is a tetraradical character index.

Energy gaps between different states are calculated as follows: singlet-triplet gap $\Delta E_{S-T} = E_T - E_S$, triplet-quintet gap $\Delta E_{T-Q} = E_Q - E_T$, and singlet-quintet gap $\Delta E_{S-Q} = E_Q - E_S$.

S3. Data Obtained in the Calculations

S3.1. DFT Benchmark and Hartree-Fock Results

A benchmark of calculation of energies of spin states with different exchange-correlation functional for the parent compound **PH** is given in Table S1a. It is noteworthy that the energy difference between triplet and closed-shell singlet ($E_T - E_{CSS}$) states varies greatly in the range of $[-12.86, 4.45]$ kcal/mol. Indeed, a change of sign of ($E_T - E_{CSS}$) directly indicates that DFT would predict different relative ordering of triplet and closed-shell singlet states. Notably, the sign of the singlet-triplet gap ($\Delta E_{S-T} = E_T - E_{OSS}$) is consistent across all exchange-correlation functionals used for the benchmark, but there is still a significant variance, which would translate into immense variability in properties. Hence, different functional would have predicted qualitatively different ground-state electronic structures and properties of **PH**, also exemplified by the great variance of $E_T - E_{OSS}$ and

$E_{OSS} - E_{CSS}$. Therefore, the results of this DFT benchmark should serve as a “cautionary tale” when researching diradicals/diradicaloids, because the observed diradical character, singlet-triplet gap, and other relevant properties might vary greatly as a function of used exchange-correlation functional. Hartree-Fock calculation results for the series of studied diradical(oid)s are given in Table S1b. These results show that from the standpoint of Hartree-Fock formalism, every compound that is presented in this work has an open-shell electronic structure. This is well-established by the differences in the energy of RHF and UHF (singlet or triplet) solutions, with **PA** showing the largest difference between closed-shell and open-shell solutions, amounting to 125 kcal/mol greater stability of open-shell than closed-shell electronic structure.

Table S1. Results from monoconfigurational methods: (a) DFT benchmark with different exchange-correlation functionals, (b) Restricted Hartree-Fock (RHF) and lowest-energy unrestricted Hartree-Fock (UHF) results across diradical(oid) derivatives of **PH**.

(a) DFT benchmark results for **PH**. Energy differences (in kcal/mol) between triplet (E_T), closed-shell singlet (E_{CSS}), and open-shell singlet (E_{OSS}) states.

Functional	$E_T - E_{CSS}$	$E_T - E_{OSS}$	$E_{OSS} - E_{CSS}$
**OLYP**	3.97	4.12	-0.14
**PBE**	4.09	4.21	-0.12
**PW91**	4.00	4.15	-0.15
**OPBE**	3.37	3.73	-0.36
**revPBE-D3**	3.76	3.98	-0.22
**BLYP-D3**	4.45	4.50	-0.04
**MS1-D3**	-0.45	3.10	-3.55
**PBE0**	-5.67	2.12	-7.79
**OPBE0**	-6.54	2.02	-8.56
**O3LYP**	2.20	3.53	-1.33
**X3LYP**	-3.47	2.27	-5.74
**B3LYP**	-2.86	2.32	-5.18
**mPW1PW**	-5.98	2.10	-8.08
**mPW1K**	-12.86	2.16	-15.02
**M06**	-4.87	2.16	-7.03
**M06-D3**	-4.86	2.16	-7.02
**M06-2X**	-7.98	1.70	-9.68
**TPSSH**	-1.12	2.53	-3.65

(b) Energy differences (in kcal/mol) between RHF and lowest-energy UHF solutions for CSG. Triplet ground state: OST GS. Open-shell singlet ground state: OSS GS.

Compound	$E_{CSS} - E_{OS}$	Comment
**PH**	72.55	
**PH1**	78.02	
**PH2**	90.37	
**PCN**	79.08	
**PCN1**	91.53	
**PCN2**	111.56	
**PN**	54.92	OST GS
**PN1**	55.68	OST GS
**PN2**	48.19	OSS GS
**PM**	78.43	
**PM1**	84.30	
**PM2**	99.10	
**PD**	75.90	
**PD1**	78.53	
**PD2**	62.82	
**PD3**	55.26	OST GS
**PAH**	110.06	
**PA**	124.61	
**PF**	115.08	

S3.2. CASSCF and CASCI Results for Diradicals and Tetraradical(oid) PT

For diradical(oid) molecules presented in this study the diradical character and singlet-triplet gap for each geometry (closed-shell singlet geometry as CSG and triplet geometry as TG) is given as a figure and accompanying table in Figure S5.

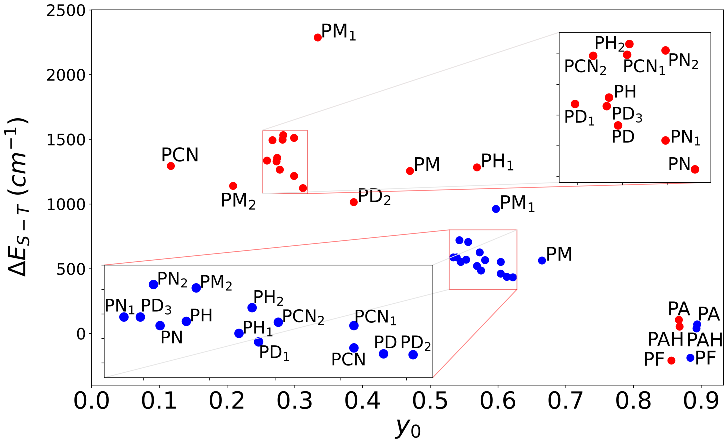

(a)

Compound	CSG			TG		
	y_0	ΔE_{S-T} (cm^{-1})	ΔE_{S-T} (kcal/mol)	y_0	ΔE_{S-T} (cm^{-1})	ΔE_{S-T} (kcal/mol)
**PH**	0.274	1357.05	3.88	0.553	570.10	1.63
**PH1**	0.569	1283.60	3.67	0.569	521.14	1.49
**PH2**	0.283	1531.93	4.38	0.573	626.06	1.79
**PCN**	0.117	1294.09	3.70	0.604	461.68	1.32
**PCN1**	0.282	1496.95	4.28	0.604	552.61	1.58
**PCN2**	0.267	1493.45	4.27	0.581	566.60	1.62
**PN**	0.312	1122.71	3.21	0.545	552.61	1.58
**PN1**	0.299	1217.15	3.48	0.534	587.59	1.68
**PN2**	0.299	1510.94	4.32	0.543	720.50	2.06
**PM**	0.470	1255.62	3.59	0.665	563.11	1.61
**PM1**	0.334	2287.40	6.54	0.597	961.83	2.75
**PM2**	0.209	1140.20	3.26	0.556	706.51	2.02
**PD**	0.278	1266.11	3.62	0.613	437.19	1.25
**PD1**	0.259	1336.06	3.82	0.575	486.16	1.39
**PD2**	0.387	1014.29	2.90	0.622	433.70	1.24
**PD3**	0.273	1329.07	3.80	0.539	587.59	1.68
**PAH**	0.868	52.46	0.15	0.893	38.47	0.11
**PA**	0.867	104.93	0.30	0.894	69.95	0.20
**PF**	0.856	-209.85	-0.60	0.884	-188.87	-0.54

(b)

Figure S5. Calculated diradical characters (y_0) and singlet-triplet (ΔE_{S-T}) gaps presented as (a) figure (in cm^{-1}) and as (b) table (in units of cm^{-1} and kcal/mol), based on the results of CASSCF calculations.

For tetraradical(oid) **PT**, for which the resonance structures are given in Figure S6, UHF and ROHF results do not predict the energy ordering that is indicated by CASSCF(12,12) calculations. It is especially noteworthy that energy gaps between states of different multiplicity are diminished significantly for CASSCF results compared to

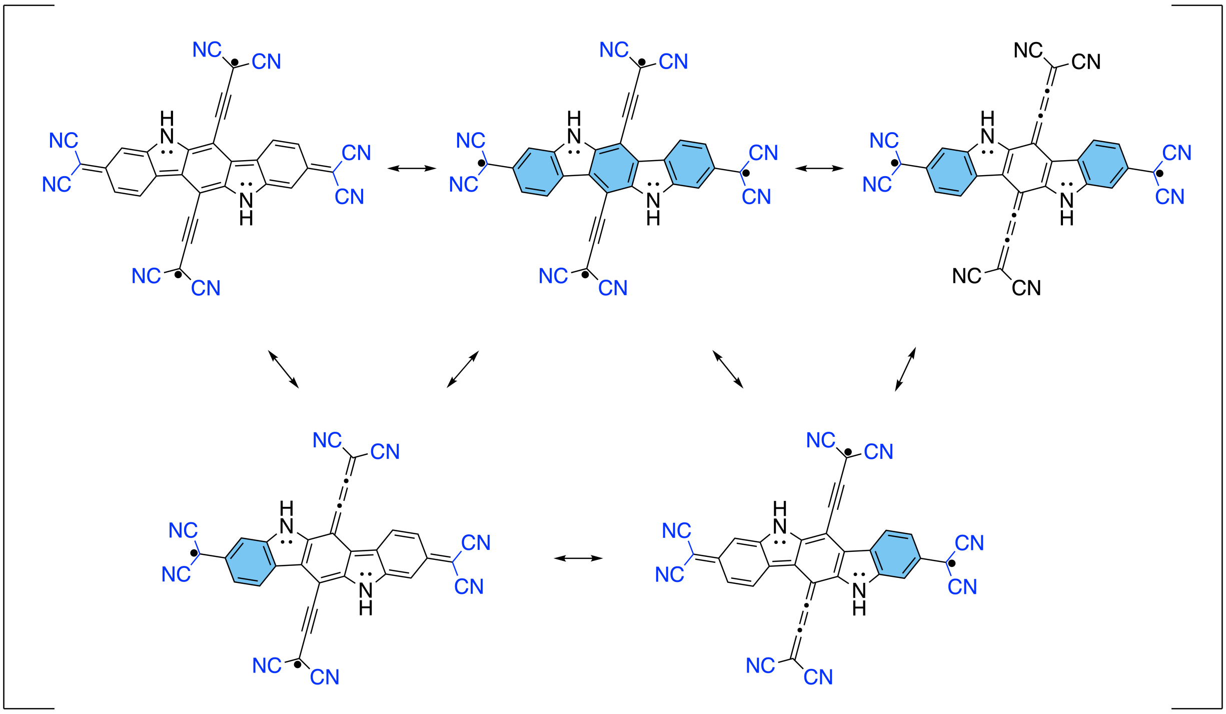

Figure S6. Resonance structures of **PT**.

UHF and ROHF results. Nevertheless, these discrepancies can be expected. UHF method is known to mix several pure states into a formally monoconfigurational wave function. These pure states can be obtained by CASSCF and CASCI methods, which show 6 low-lying states, some of which can be mixed together in a UHF wave function. This consideration explains the observed discrepancy. In order to describe the wave functions/electronic structures of these multiconfigurational systems (at least) qualitatively accurately, we use the multireference methods such as CASSCF and CASCI. Even if the quintet is not a ground state, it is very important from the chemical point of view that it is at least energetically close to the states of other multiplicities.

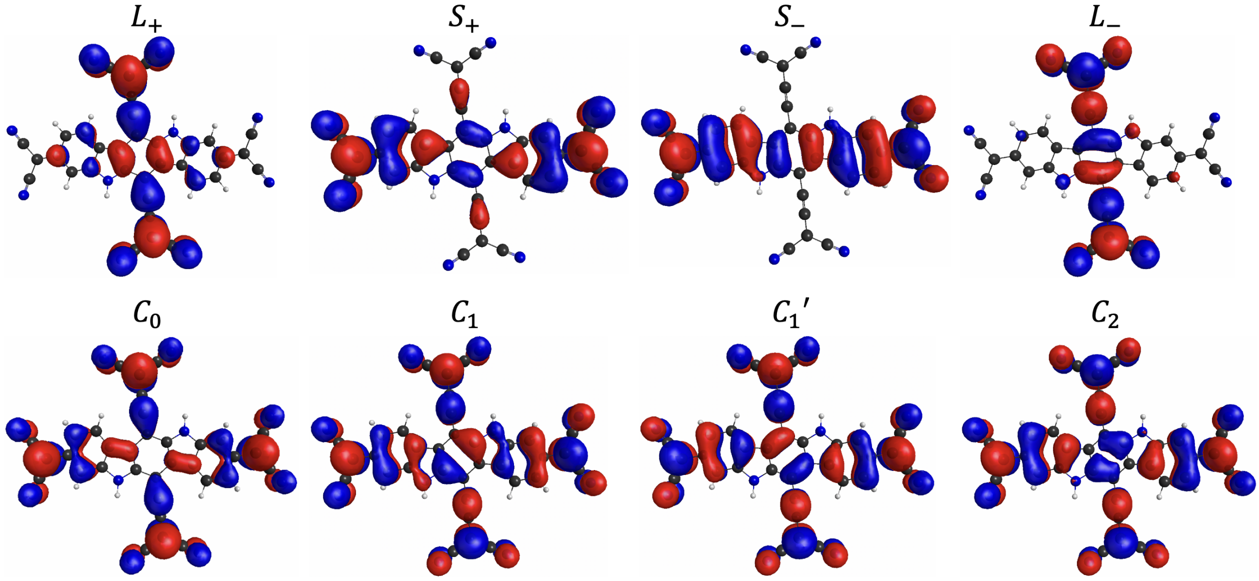

Figure S7. CASSCF natural orbitals of **PT** with symbolic assignations. Isosurfaces with the value of 0.015.

Results of CASSCF(4,4) are given in Table S2 and CASSCF(12,12) results are given in Table S3 (for DFT quintet-optimized geometry), with orbital assignations in Figure S7. As we see the range of energies for low-lying states range is 2177 cm^{-1}, and the first excited state with the multiplicity of triplet is within 432 cm^{-1} (1.24 kcal/mol) of the open-shell singlet ground state, which means it is thermally accessible and thus, **PT** should show a signal in electron paramagnetic resonance (EPR) spectroscopy experiment. Since the energy gaps are

Table S2. CASSCF(4,4) states of **PT** determined by state-specific calculations for DFT quintet-optimized geometry. For all the calculations, UHF quintet NOs were used as initial guess. Under HONO, LUNO, etc. three columns represent the symmetry of the orbital, its symbolic representation according to Figure S7 and NO occupation number.

State	Symmetry	Energy (a.u.)	CASSCF NOs symmetry, identity and occupation number.												ΔE from G. S. (cm^{-1})
			HONO − 1			HONO			LUNO			LUNO + 1			
S_0	A_g	-1836.5108422277	A_u	L_+	1.487	A_u	S_+	1.110	B_g	S_-	0.886	B_g	L_-	0.518	0.00
T_0	B_u	-1836.5098812400	A_u	L_+	1.502	B_g	S_-	1.002	A_u	S_+	0.995	B_g	L_-	0.501	210.91
T_1	B_u	-1836.5039231977	A_u	S_+	1.142	A_u	L_+	1.004	B_g	L_-	0.987	B_g	S_-	0.868	1518.55
S_1	A_g	-1836.5035796490	A_u	C_0	1.126	B_g	C_1	1.010	B_g	$C_1^{'}$	0.967	A_u	C_2	0.898	1593.95
T_2	A_g	-1836.5034380788	A_u	C_0	1.091	B_g	C_1	1.048	B_g	$C_1^{'}$	0.952	A_u	C_2	0.909	1625.02
Q_0	A_g	-1836.5027117298	A_u	C_0	1.000	B_g	C_1	1.000	A_u	C_2	1.000	B_g	$C_1^{'}$	1.000	1784.44

Table S3. CASSCF(12,12) states of **PT** determined by state-specific calculations for DFT quintet-optimized geometry. For all the calculations, UHF quintet NOs were used as initial guess. Under HONO, LUNO, etc. three columns represent the symmetry of the orbital, its symbolic representation according to Figure S7 and NO occupation number.

State	Symmetry	Energy (a.u.)	CASSCF NOs symmetry, identity and occupation number												ΔE from G. S. (cm^{-1})
			HONO − 1			HONO			LUNO			LUNO + 1			
S_0	A_g	-1836.6003248840	A_u	L_+	1.497	A_u	S_+	1.122	B_g	S_-	0.872	B_g	L_-	0.511	0.00
T_0	B_u	-1836.5983566998	A_u	L_+	1.540	B_g	S_-	1.000	A_u	S_+	0.996	B_g	L_-	0.465	431.97
T_1	B_u	-1836.5925672155	A_u	S_+	1.154	A_u	L_+	1.033	B_g	L_-	0.957	B_g	S_-	0.858	1702.61
S_1	A_g	-1836.5914852556	A_u	C_0	1.140	B_g	C_1	1.001	B_g	$C_1^{'}$	0.963	A_u	C_2	0.897	1940.07
T_2	A_g	-1836.5914276798	A_u	C_0	1.106	B_g	C_1	1.054	B_g	$C_1^{'}$	0.943	A_u	C_2	0.898	1952.71
Q_0	A_g	-1836.5904040182	A_u	C_0	1.005	B_g	L_-	1.000	A_u	$C_1^{'}$	0.999	B_g	S_-	0.997	2177.38

Table S4. CASCI(4,4) for **PT** from CASSCF(12,12) quintet converged orbitals as initial guess for DFT quintet-optimized geometry. Under HONO, LUNO, etc. three columns represent the symmetry of the orbital, its symbolic representation according to Figure S7 and n_{NO}.

State	Symmetry	Energy (a.u.)	CASCI NOs occupancy, identity and symmetry												ΔE from G. S. (cm^{-1})
			HONO − 1			HONO			LUNO			LUNO + 1			
S_0	A	-1836.4954786613	A	C_0	1.285	A	C_2	1.110	A	C_1	0.893	A	$C_1^{'}$	0.722	0.00
T_0	A	-1836.4936832384	A	L_+	1.238	A	S_+	0.999	A	S_-	0.997	A	L_-	0.767	394.05
T_1	A	-1836.4911158413	A	S_+	1.166	A	L_+	1.021	A	L_-	0.969	A	S_-	0.843	957.53
T_2	A	-1836.4900247041	A	C_0	1.110	A	C_1	1.054	A	$C_1^{'}$	0.946	A	C_2	0.890	1197.01
S_1	A	-1836.4899838313	A	C_0	1.146	A	C_1	1.012	A	$C_1^{'}$	0.956	A	C_2	0.886	1205.98
Q_0	A	-1836.4892522623	A	D_0 [a]	1.000	A	D_1	1.000	A	D_2	1.000	A	D_3	1.000	1366.54

[a] D_0, D_1, D_2, D_3 means **distorted** orbitals with the same overall electron density as from C_0, C_1, $C_1^{'}$, C_2 orbitals.

Table S5. CASCI(12,12) for **PT** from CASSCF(12,12) quintet converged orbitals as initial guess for DFT quintet-optimized geometry. Under HONO, LUNO, etc. three columns represent the symmetry of the orbital, its symbolic representation according to Figure S7 and n_{NO}.

State	Symmetry	Energy (a.u.)	CASCI NOs occupancy, identity and symmetry.												ΔE from G. S. (cm^{-1})
			HONO − 1			HONO			LUNO			LUNO + 1			
S_0	A	-1836.5964215286	A	C_0	1.263	A	C_2	1.077	A	C_1	0.918	A	$C_1^{'}$	0.744	0.00
T_0	A	-1836.5948052330	A	L_+	1.236	A	S_+	0.999	A	S_-	0.997	A	L_-	0.768	354.74
T_1	A	-1836.5921396709	A	S_+	1.128	A	L_+	1.013	A	L_-	0.975	A	S_-	0.885	939.76
S_1	A	-1836.5912545906	A	C_0	1.122	A	C_1	1.012	A	$C_1^{'}$	0.958	A	C_2	0.908	1134.01
T_2	A	-1836.5912370713	A	C_0	1.094	A	C_1	1.046	A	$C_1^{'}$	0.951	A	C_2	0.910	1137.86
Q_0	A	-1836.5904039925	A	C_0	1.005	A	L_-	1.000	A	C_2	0.999	A	S_-	0.995	1320.70

still significant, it means there are quite strong couplings between unpaired electrons. According to occupation numbers of natural orbitals of the S_0 state of **PT** from CASSCF(12,12) calculations with DFT quintet-optimized geometry, the diradical character is $y_0 = 0.75$ and the tetraradical character is $y_1 = 0.21$. For further exploration of the wave functions of the different spin states of **PT** and calculation of exchange-coupling constants (see Section S3.3), we can refer to the CASCI(4,4) given in Table S4 and CASCI(12,12) given in Table S5, with determinant contributions into the wave functions for CASCI(4,4) given in Table S6 and for CASCI(12,12) given in Table S7. As we see from CASCI(4,4) and CASCI(12,12) determinant contributions into the wave function of the states, we can notice that overall sum of weights of coefficients varies little across different spin states, and represents more than 97 % of the wave function for all states in case of CASCI(4,4) and more than 79 % for all states in case of CASCI(12,12).

Table S6. CASCI(4,4) determinant contributions for 6 close-lying tetraradical(oid) spin states of **PT** (for DFT quintet-optimized geometry), which are obtained for $S_z = 0$ basis from CASSCF(12,12) converged quintet localized (in (4,4) SOMOs subspace) initial guess orbitals.

	S_0	T_0	T_1	T_2	S_1	Q_0
ΔE from ground state (cm^{-1})	0.00	394.05	957.53	1197.01	1205.98	1366.54
$\vert\alpha\alpha\beta\beta\rangle$	0.3958810999	0.2795608806	-0.6447773391	0.000000000	-0.4130501947	0.4082482905
$\vert\beta\beta\alpha\alpha\rangle$	0.3958810999	-0.2795608806	0.6447773391	0.000000000	-0.4130501947	0.4082482905
$\vert\alpha\beta\alpha\beta\rangle$	0.5521546897	0.6415961579	0.2806164626	0.000000000	0.1393648361	-0.4082482905
$\vert\beta\alpha\beta\alpha\rangle$	0.5521546897	-0.6415961579	-0.2806164626	0.000000000	0.1393648361	-0.4082482905
$\vert\alpha\beta\beta\alpha\rangle$	0.1562735898	0.000000000	0.000000000	0.7044101983	0.5524150308	0.4082482905
$\vert\beta\alpha\alpha\beta\rangle$	0.1562735898	0.000000000	0.000000000	-0.7044101983	0.5524150308	0.4082482905
Sum of coefficients $\sum_{I\in CAS} c_I^2$	0.9720	0.9796	0.9890	0.9924	0.9904	1.0000

Table S7. CASCI(12,12) determinant contributions for 6 close-lying tetraradical(oid) spin states of **PT** (for DFT quintet-optimized geometry), which are obtained for $S_z = 0$ basis from CASSCF(12,12) converged quintet localized (in (4,4) SOMOs subspace) initial guess orbitals.

	S_0	T_0	T_1	S_1	T_2	Q_0
ΔE from ground state (cm^{-1})	0.00	354.74	939.76	1134.01	1137.86	1320.70
$\vert 2222\alpha\alpha\beta\beta 0000\rangle$	0.3581901120	0.2762816378	-0.5707362873	-0.3722456574	0.000000000	0.3677921938
$\vert 2222\beta\beta\alpha\alpha 0000\rangle$	0.3581901120	-0.2762816377	0.5707362874	-0.3722456573	0.000000000	0.3677921939
$\vert 2222\alpha\beta\alpha\beta 0000\rangle$	0.4993670583	0.5676260098	0.2792249443	0.1261748951	0.000000000	-0.3677928554
$\vert 2222\beta\alpha\beta\alpha 0000\rangle$	0.4993670584	-0.5676260098	-0.2792249444	0.1261748950	0.000000000	-0.3677928554
$\vert 2222\alpha\beta\beta\alpha 0000\rangle$	0.1411769283	0.000000000	0.000000000	0.4984175845	0.6352876335	0.3677945700
$\vert 2222\beta\alpha\alpha\beta 0000\rangle$	0.1411769283	0.000000000	0.000000000	0.4984175737	-0.6352876251	0.3677945699
Sum of coefficients $\sum_{I\in CAS} c_I^2$	0.7952	0.7971	0.8074	0.8058	0.8072	0.8116

S3.3. Calculations of Exchange-Coupling Constants Between Radical Centers

Heisenberg-Dirac-van Vleck Hamiltonian[31–34] is a model spin Hamiltonian for two-dimensional systems. This model considers open-shell systems as a particle-per-site model of spin centers. See the full description of the theoretical details of this method in the Supporting Information of our recent Letter.[4] This model Hamiltonian has the form given in Eq. S1:[34]

$$\hat{H}_{HDvV} = -\sum_{i<j} J_{ij}\hat{\boldsymbol{S}}_i \cdot \hat{\boldsymbol{S}}_j \tag{S1}$$

We can expand Heisenberg-Dirad-van Vleck Hamiltonian for our two-particle systems as given in Eq. S2:

$$\hat{H}_{HDvV} = -J_{12}\hat{\boldsymbol{S}}_1 \cdot \hat{\boldsymbol{S}}_2 \tag{S2}$$

and for our four-particle systems as given in Eq. S3:

$$\hat{H}_{HDvV} = -J_{12}\hat{\boldsymbol{S}}_1 \cdot \hat{\boldsymbol{S}}_2 - J_{13}\hat{\boldsymbol{S}}_1 \cdot \hat{\boldsymbol{S}}_3 - J_{14}\hat{\boldsymbol{S}}_1 \cdot \hat{\boldsymbol{S}}_4 - J_{23}\hat{\boldsymbol{S}}_2 \cdot \hat{\boldsymbol{S}}_3 - J_{24}\hat{\boldsymbol{S}}_2 \cdot \hat{\boldsymbol{S}}_4 - J_{34}\hat{\boldsymbol{S}}_3 \cdot \hat{\boldsymbol{S}}_4 \tag{S3}$$

By applying these $\hat{H}_{HDvV}$ operators, we obtain our model Hamiltonian matrix for a two-particle system as given in Eq. S4. This model has 2 spin centers and our basis is accordingly 2 determinants from $S_z = 0$ subspace with two particles: $|\alpha(1)\beta(2)\rangle$ and $|\beta(1)\alpha(2)\rangle$.

$$\hat{H}_{HDvV} = \begin{pmatrix} \frac{J_{12}}{4} & -\frac{J_{12}}{2} \\ -\frac{J_{12}}{2} & \frac{J_{12}}{4} \end{pmatrix} \tag{S4}$$

Diagonalization of this Hamiltonian provides the following eigenvalues (Eq. S5) and eigenvectors (Eq. S6):

$$E_0 = -\frac{J_{12}}{4} \qquad E_1 = \frac{3J_{12}}{4} \tag{S5}$$

$$^1\psi_0 = \frac{1}{\sqrt{2}}|\alpha\beta\rangle + \frac{1}{\sqrt{2}}|\beta\alpha\rangle \qquad ^3\psi_1 = \frac{1}{\sqrt{2}}|\alpha\beta\rangle - \frac{1}{\sqrt{2}}|\beta\alpha\rangle \tag{S6}$$

We can calculate the exchange-coupling constant by taking the energy difference between singlet and triplet states, which is a singlet-triplet gap $\Delta E_{S-T} = E_T - E_S = \frac{3J_{12}}{4} - (-\frac{J_{12}}{4}) = J_{12}$. Values of exchange-coupling constants between two radical centers in diradical(oid)s presented in this work is given in Figure S8. Comparing these exchange-coupling constant values to diradical character indices given in Figure S5, we see that similarly to y_0 and ΔE_{S-T}, values of y_0 and J_{S-T} do not correlate.

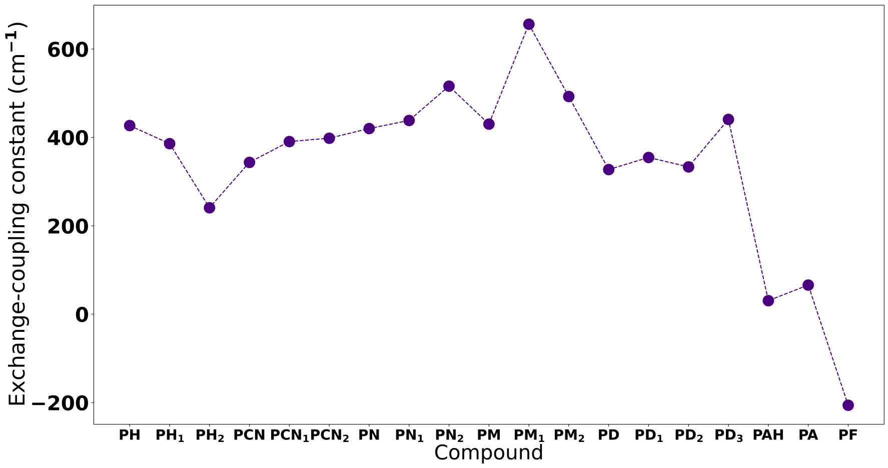

(a)

Compound	y_0	ΔE_{S-T} (cm^{-1})	ΔE_{S-T} (kcal/mol)
**PH**	0.553	426.72	1.22
**PH1**	0.569	385.83	1.10
**PH2**	0.573	240.81	0.69
**PCN**	0.604	343.48	0.98
**PCN1**	0.604	390.57	1.12
**PCN2**	0.581	398.14	1.14
**PN**	0.545	419.97	1.20
**PN1**	0.534	438.34	1.25
**PN2**	0.543	515.89	1.48
**PM**	0.665	430.02	1.23
**PM1**	0.597	656.11	1.88
**PM2**	0.556	492.53	1.41
**PD**	0.613	327.20	0.94
**PD1**	0.575	354.63	1.01
**PD2**	0.622	333.23	0.95
**PD3**	0.539	440.73	1.26
**PAH**	0.893	30.59	0.09
**PA**	0.894	65.86	0.19
**PF**	0.884	-206.05	-0.59

(b)

Figure S8. Values of exchange-coupling constants (J_{12}) in presented diradical(oid) compounds for the triplet-optimized geometry as (a) figure and (b) table (with diradical characters).

The HDvV Hamiltonian for a four-particle system is given in Eq. S7. In this case, the basis is the set of 6 determinants from $S_z = 0$ subspace with four particles: $|\alpha\alpha\beta\beta\rangle$, $|\beta\beta\alpha\alpha\rangle$, $|\alpha\beta\alpha\beta\rangle$, $|\beta\alpha\beta\alpha\rangle$, $|\alpha\beta\beta\alpha\rangle$, $|\beta\alpha\alpha\beta\rangle$. The orbital ordering is given in Figure S3.

$$\hat{H}_{HDvV} = \begin{pmatrix} H_{1,1} & 0 & -\frac{J_{23}}{2} & -\frac{J_{14}}{2} & -\frac{J_{24}}{2} & -\frac{J_{13}}{2} \\ 0 & H_{2,2} & -\frac{J_{14}}{2} & -\frac{J_{23}}{2} & -\frac{J_{13}}{2} & -\frac{J_{24}}{2} \\ -\frac{J_{23}}{2} & -\frac{J_{14}}{2} & H_{3,3} & 0 & -\frac{J_{34}}{2} & -\frac{J_{12}}{2} \\ -\frac{J_{14}}{2} & -\frac{J_{23}}{2} & 0 & H_{4,4} & -\frac{J_{12}}{2} & -\frac{J_{34}}{2} \\ -\frac{J_{24}}{2} & -\frac{J_{13}}{2} & -\frac{J_{34}}{2} & -\frac{J_{12}}{2} & H_{5,5} & 0 \\ -\frac{J_{13}}{2} & -\frac{J_{24}}{2} & -\frac{J_{12}}{2} & -\frac{J_{34}}{2} & 0 & H_{6,6} \end{pmatrix} \tag{S7}$$

With the following diagonal elements:

$$\begin{aligned} H_{1,1} &= -\frac{J_{12}}{4} + \frac{J_{13}}{4} + \frac{J_{14}}{4} + \frac{J_{23}}{4} + \frac{J_{24}}{4} - \frac{J_{34}}{4} \\ H_{2,2} &= -\frac{J_{12}}{4} + \frac{J_{13}}{4} + \frac{J_{14}}{4} + \frac{J_{23}}{4} + \frac{J_{24}}{4} - \frac{J_{34}}{4} \\ H_{3,3} &= \frac{J_{12}}{4} - \frac{J_{13}}{4} + \frac{J_{14}}{4} + \frac{J_{23}}{4} - \frac{J_{24}}{4} + \frac{J_{34}}{4} \\ H_{4,4} &= \frac{J_{12}}{4} - \frac{J_{13}}{4} + \frac{J_{14}}{4} + \frac{J_{23}}{4} - \frac{J_{24}}{4} + \frac{J_{34}}{4} \\ H_{5,5} &= \frac{J_{12}}{4} + \frac{J_{13}}{4} - \frac{J_{14}}{4} - \frac{J_{23}}{4} + \frac{J_{24}}{4} + \frac{J_{34}}{4} \\ H_{6,6} &= \frac{J_{12}}{4} + \frac{J_{13}}{4} - \frac{J_{14}}{4} - \frac{J_{23}}{4} + \frac{J_{24}}{4} + \frac{J_{34}}{4} \end{aligned} \tag{S8}$$

To calculate exchange-coupling constants, we need to build an effective Hamiltonian by the method of Bloch and orthonormalize by the approach of des Cloizeaux or Gram-Schmidt.[35,36,46] Since determination of exchange-coupling constants for two-particle systems was trivial, let us build an effective Hamiltonian for a four-particle model. For tetraradical(oid) **PT**, the effective Hamiltonian built from the results of CASCI(4,4) calculations is given in Eq. S9 and built from results of CASCI(12,12) calculations is given in Eq. S10:

$$\hat{H}_{eff}(\text{CASCI(4,4)}) = \begin{pmatrix} 869.12 & 1.50 & -401.13 & -194.78 & -49.99 & -49.99 \\ 1.50 & 869.12 & -194.78 & -401.13 & -49.99 & -49.99 \\ -401.13 & -194.78 & 493.48 & 9.52 & -133.81 & -133.81 \\ -194.78 & -401.13 & 9.52 & 493.48 & -133.81 & -133.81 \\ -49.99 & -49.99 & -133.81 & -133.81 & 1197.95 & 0.95 \\ -49.99 & -49.99 & -133.81 & -133.81 & 0.95 & 1197.95 \end{pmatrix} \tag{S9}$$

$$\hat{H}_{eff}(\text{CASCI(12,12)}) = \begin{pmatrix} 829.01 & 1.30 & -401.30 & -171.09 & -41.00 & -41.00 \\ 1.30 & 829.01 & -171.09 & -401.30 & -41.00 & -41.00 \\ -401.30 & -171.09 & 475.90 & 9.11 & -131.65 & -131.65 \\ -171.09 & -401.30 & 9.11 & 475.90 & -131.65 & -131.65 \\ -41.00 & -41.00 & -131.65 & -131.65 & 1138.63 & 0.77 \\ -41.00 & -41.00 & -131.65 & -131.65 & 0.77 & 1138.63 \end{pmatrix} \tag{S10}$$

From comparing Eq. S7, Eq. S9 and Eq. S10, we can determine the values of each exchange-coupling constant. For CASCI(4,4) results, we see that largest exchange-coupling constant value is $J_{23} = J_L = 802.26$ cm^{-1} and the second largest is $J_{14} = J_S = 389.56$ cm^{-1}, followed by $J_{12} = J_{34} = J_a = 267.62$ cm^{-1} and $J_{13} = J_{24} = J_b = 99.98$ cm^{-1}. For CASCI(12,12) results, exchange-coupling constant values are $J_L = 802.60$ cm^{-1}, $J_S = 342.18$ cm^{-1}, $J_a = 263.30$ cm^{-1} and $J_b = 82.00$ cm^{-1}. According to adopted definition of the HDvV Hamiltonian in Eq. S1, the positive values mean antiferromagnetic coupling between spin centers, while the negative values mean ferromagnetic coupling between spin centers.

S4. Geometry Comparison for Diradical(oid)s and Tetraradical(oid)s

Differences in RKS/DFT (CSS) and UKS(triplet)/DFT geometries (CSG and TG) between **PH** derivatives are not dramatic and are usually ≤ 0.01Å for corresponding bonds. However, certain bonds do pronounce the difference between quinoidal closed-shell and diradical aromatic resonance structures depicted in Figure S2a more significantly. For example, for **PH**, a bond "3" between a dicyanomethylene substituent and a terminal benzenoid ring is 1.426 Å, and a bond "g" between benzenoid rings is 1.420 Å with RKS/DFT, while these bonds are 1.447 (+0.021) Å and 1.443 (+0.023) Å, respectively, when optimized with UKS(triplet)/DFT (Figure S9). In a quinoidal closed-shell resonance structure, these bonds appear as double, while for a diradical aromatic resonance structure, these bonds appear as single (Figure S2a). Since RKS/DFT is supposed to model a closed-shell (and thus, in our case, quinoidal) structure, while UKS(triplet)/DFT is supposed to model an open-shell (and thus, in our case, biradical aromatic) structure, these differences in RKS/DFT and UKS(triplet)/DFT geometries reflect differences between two resonance structures (quinoidal and aromatic) of **PH**. Also, for benzenoid rings it is important to point out that RKS/DFT-optimized geometry have their bond lengths vary in a wider range for terminal ($[1.376, 1.447]$ Å) and central benzene rings ($[1.384, 1.460]$ Å) than UKS(triplet)/DFT-optimized geometry, which shows variance of bond lengths in the range of $[1.386, 1.436]$ Å for terminal rings and $[1.393, 1.445]$ Å for the central rings. Other bond-length differences in benzenoid rings between RKS/DFT (CSS) and UKS(triplet)/DFT results follow the trend: if a bond is double in a quinoidal resonance structure, RKS/DFT gives its length to be about 0.01 Å shorter, but if a bond is single in quinoidal resonance structure, RKS/DFT gives its length to be about 0.01 Å longer compared to UKS(triplet)/DFT. A detailed geometry comparison between corresponding bonds for both closed-shell singlet and triplet DFT-optimized geometries of **PH** is given in Figure S9.

For **PT**, a quintet-optimized geometry has longer bonds where they are expected from its resonance structures. For example, bonds "3", "m" and bond "g" appear as single bonds in the tetraradical resonance structure, but at least one of them appears as double in diradical resonance structures. These bonds are the longest for the quintet-optimized geometry, as would be expected based on resonance structures. Other corresponding bonds

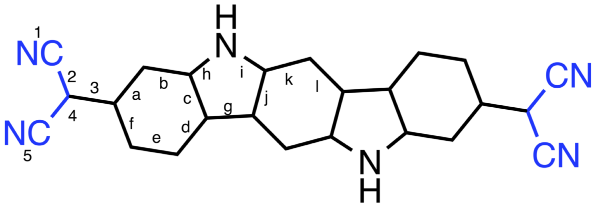

(a)

Label of a bond	RKS/DFT (CSS) geometry	UKS (triplet)/ DFT geometry
1	1.171	1.172
2	1.421	1.418
3	1.426	1.447
4	1.421	1.417
5	1.170	1.172
a	1.433	1.423
b	1.379	1.389
c	1.445	1.436
d	1.422	1.412
e	1.376	1.386
f	1.447	1.436
g	1.420	1.443
h	1.394	1.392
i	1.394	1.398
j	1.460	1.445
k	1.384	1.393
l	1.417	1.408

(b)

Figure S9. Comparison of RKS/DFT (CSS) and UKS(triplet)/DFT-optimized geometries (CSG and TG) of **PH** for bond lengths. Both optimized geometries belong to the C_{2h} point group. Bond lengths are given in the units of Å. Exchange-correlation functional: BLYP, basis set: Slater-type TZP.

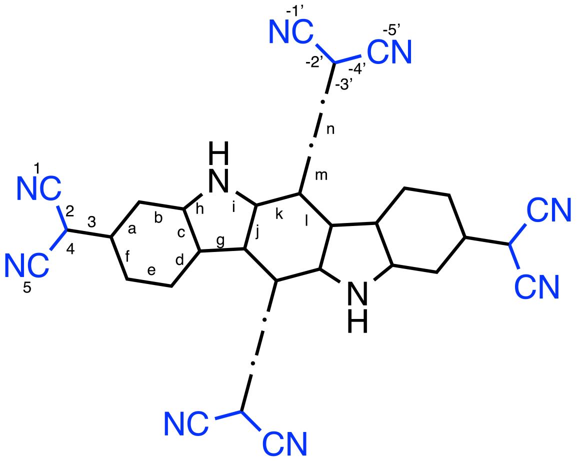

(a)

Label of a bond	RKS/DFT (CSS) geometry	UKS (triplet)/ DFT geometry	UKS (quintet)/ DFT geometry
1	1.170	1.171	1.171
2	1.420	1.419	1.417
3	1.428	1.443	1.449
4	1.420	1.418	1.416
5	1.170	1.171	1.171
a	1.431	1.422	1.422
b	1.380	1.388	1.388
c	1.442	1.438	1.434
d	1.423	1.418	1.414
e	1.378	1.382	1.386
f	1.444	1.440	1.434
g	1.424	1.434	1.443
h	1.399	1.393	1.397
i	1.371	1.378	1.379
j	1.432	1.422	1.436
k	1.438	1.441	1.426
l	1.445	1.447	1.427
m	1.373	1.371	1.403
n	1.243	1.243	1.231
-1'	1.169	1.169	1.171
-2'	1.428	1.428	1.421
-3'	1.367	1.367	1.392
-4'	1.427	1.428	1.421
-5'	1.169	1.169	1.171

(b)

Figure S10. Comparison of RKS/DFT (CSS), UKS(triplet)/DFT and UKS(quintet)/DFT-optimized geometries (CSG, TG and QG) of **PT** for bond lengths. All optimized geometries belong to the C_{2h} point group. Bond lengths are given in the units of Å. Exchange-correlation functional: BLYP, basis set: Slater-type TZP.

follow the same tendency of difference between different spin state optimized geometries as diradical(oid)s. For **PT**, some bonds pronounce greater differences than most, such as bonds "3" and "m".

For **PTP** a quintet-optimized geometry also has longer bonds where they are expected from resonance structures, similarly to **PT**. Nonetheless, these differences are less pronounced for corresponding bonds than in **PTP**. This greater similarity between optimized geometries of different spin states for **PTP** means that **PTP** will exhibit greater parallelism between the results of electronic structure calculations (CASCI and CASSCF) for different optimized geometries. This, indeed, has been verified to be the case in our preliminary calculations (not shown here).

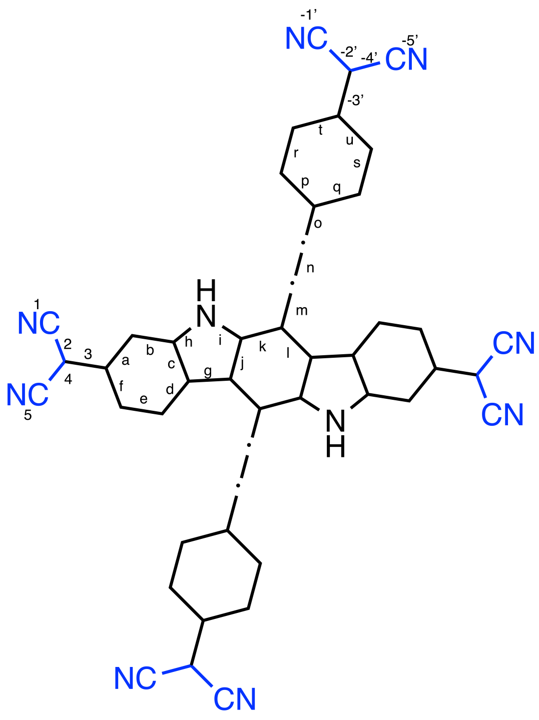

Label of a bond	RKS/DFT (CSS) geometry	UKS (triplet)/ DFT geometry	UKS (quintet)/ DFT geometry
1	1.170	1.170	1.171
2	1.421	1.421	1.418
3	1.427	1.426	1.447
4	1.420	1.421	1.417
5	1.170	1.170	1.171
a	1.432	1.431	1.422
b	1.379	1.380	1.388
c	1.443	1.446	1.435
d	1.423	1.424	1.414
e	1.377	1.376	1.385
f	1.445	1.447	1.435
g	1.422	1.420	1.443
h	1.396	1.392	1.393
i	1.378	1.383	1.384
j	1.442	1.443	1.435
k	1.422	1.413	1.421
l	1.442	1.445	1.428
m	1.393	1.401	1.408
n	1.234	1.231	1.228
o	1.395	1.404	1.411
p	1.434	1.430	1.426
q	1.434	1.430	1.426
r	1.376	1.379	1.382
s	1.376	1.379	1.383
t	1.436	1.431	1.428
u	1.436	1.431	1.428
−1′	1.170	1.171	1.171
−2′	1.421	1.420	1.418
−3′	1.425	1.434	1.441
−4′	1.421	1.420	1.418
−5′	1.170	1.171	1.171

(a) **(b)**

Figure S11. Comparison of RKS/DFT (CSS), UKS(triplet)/DFT and UKS(quintet)/DFT-optimized geometries (CSG, TG, and QG) of **PTP** for bond lengths. All optimized geometries belong to the C_{2h} point group. Bond lengths are given in the units of Å. Exchange-correlation functional: BLYP, basis set: Slater-type TZP.

S5. Study of *local* and *global* Aromaticity of Diradical(oid)s and Tetraradical(oid)s

Since indolo[3,2-*b*]carbazole contains continuous π-conjugation around its perimeter, it is important to explore electron currents that can be induced by the magnetic field applied in the perpendicular direction of the plane of diradical(oid) and tetraradical(oid) molecules for which we investigated the electronic structure. Based on the variation of derivatives according to substituents, we selected some of the derivatives representing most of the variance between derivatives of **PH** based on electron-donating and electron-withdrawing properties and the length of the π-conjugated chain. Therefore, we performed ACID calculations, for which plots are presented in Figure S12. For diradical(oid)s derived from **PH**, we see that all of them (except **PAH**, **PA** and **PF** as shown by MCI results below) possess continuous electron current upon the application of the magnetic field in the perpendicular direction of the plane of indolo[3,2-*b*]carbazole subsystem. Hence, it is apparent from the results of ACID calculations that both open-shell singlet and triplet states possess "*global aromaticity*". These results also demonstrate, based on the resonance structures given in Figure S2a, that the electronic structure of these compounds must be open-shell, because such states allow for global aromaticity, while quinoidal closed-shell structure would forbid it. Furthermore, "*global aromaticity*" is observed for singlet, triplet and quintet spin states of the tetraradical(oid) **PT** (for quintet-optimized DFT geometry) and the tetraradical(oid) **PTP** (latter shown in the main text and Supporting Information of our recent Letter [4]). Since the tetraradical resonance structure of **PT** has only one more Clar's π-sextet than the diradical resonance structures, for the relative stability of the quintet spin state of **PT**, there would not be sufficient aromatic stabilization, as by analogy for *para*-quinodimethane (pQDM), which has one aromatic ring that is insufficient to stabilize open-shell electronic structure. Therefore, we should invoke the argument of "*global aromaticity*" to help explain the vicinity of higher spin states of this tetraradical(oid) to the open-shell ground state.

It is also pertinent to explore the *local* aromaticity of the benzenoid rings in the diradical(oid) and tetraradical(oid) compounds that have been verified above to possess significant open-shell character in all cases. The results of calculations of the multicenter index of aromaticity for selected compounds are given in Table S8 and with ring numbering given in Figure S13. By comparing these values of MCI to those of other aromatic compounds with benzenoid rings, we can verify that terminal benzenoid rings (and the central benzenoid ring more weakly) of diradical(oid)s and tetraradical(oid)s are indeed aromatic. [43,44] It is noteworthy that the central benzenoid ring

of **PA** is non-aromatic. We can explain this if we consider that **PA** has doubly-bonded substituents in its central ring. These bonds preclude the aromaticity of the central benzenoid ring. Furthermore, even though there is some aromaticity in the central benzenoid rings of diradical(oid)s and tetraradical(oid)s, sometimes even comparable to the degree of aromaticity of the terminal rings, the greater number of different substituents distort the sharing of electronic density within the ring, compared to other rings. Nevertheless, these results show that diradical resonance structures are the most important (or at least very significant) contributors to the "resonance hybrid" of these compounds. This is because the diradical structure allows the aromaticity of the rings in these molecules, while quinoidal would forbid it.

In order to visualize the results from MCI calculations, we can refer to Figure S14, from which it is apparent that the variability of MCI is quite small for the terminal benzenoid rings, a little higher for the pyrrole rings (which also possess weak aromaticity) and the highest for the central benzenoid ring. From this graph, we can reason that the distortion of electronic density by the substituents of different electron-donating or electron-withdrawing properties and various lengths of π-conjugated chain affect the aromaticity of the central benzenoid ring accordingly. These distortions of electron density diminish the aromaticity of the central benzenoid ring, as shown in Figure S14. Taken to the extreme, if the distortion is significant enough, it diminishes the MCI values of the ring to those corresponding to non-aromatic rings, such as for **PA**, which has doubly bonded groups to the central benzenoid ring that restrict electron delocalization in the central benzenoid ring, thus diminishing its aromaticity dramatically. Even though an exact prediction about which substituent will distort the sharing of the electron density to the greater extent in the rings is not possible, it is still clear that the aromaticity of the central benzenoid ring is diminished by these substituents. This is evident from the comparison of MCI values of the central benzenoid rings of the derivatives of **PH** given in Table S8 and Figure S14.

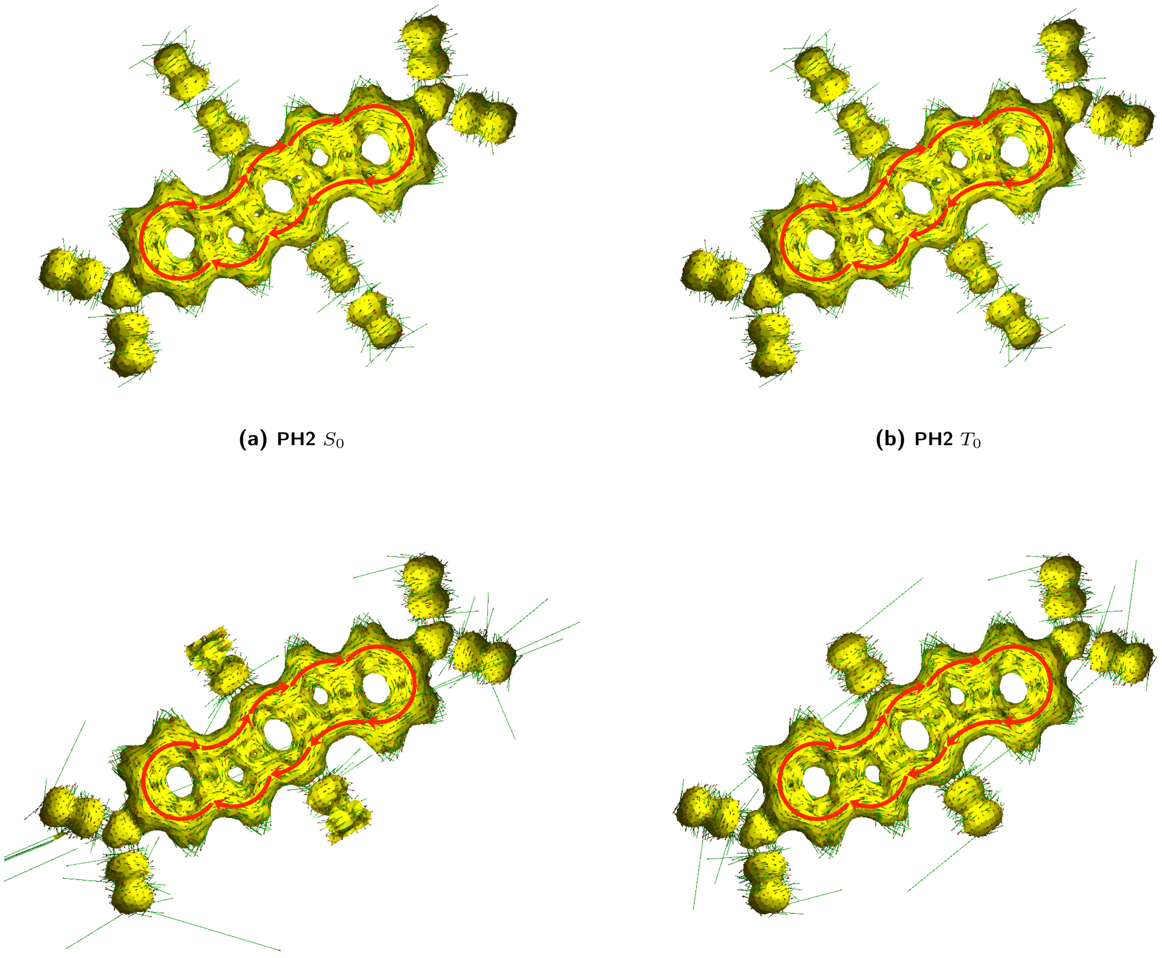

(a) **PH2** S_0 **(b)** **PH2** T_0

(c) **PCN** S_0 **(d)** **PCN** T_0

Figure S12. ACID plots for the different spin states of derivatives of **PH**. Isosurfaces with the value of 0.030.

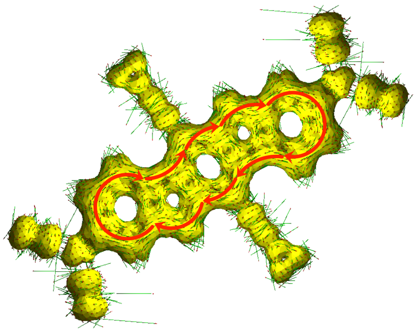

(e) PN1 S_0

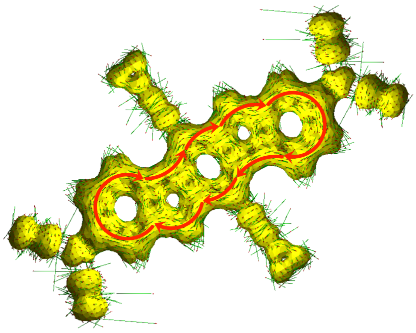

(f) PN1 T_0

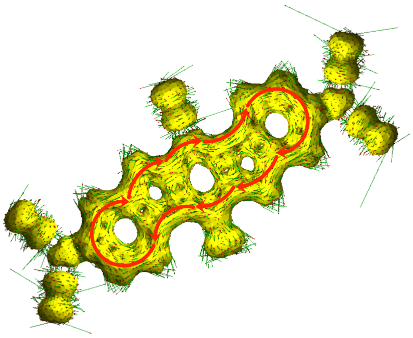

(g) PM S_0

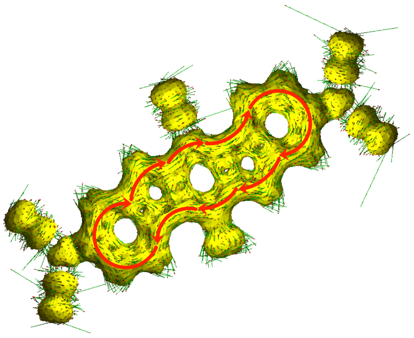

(h) PM T_0

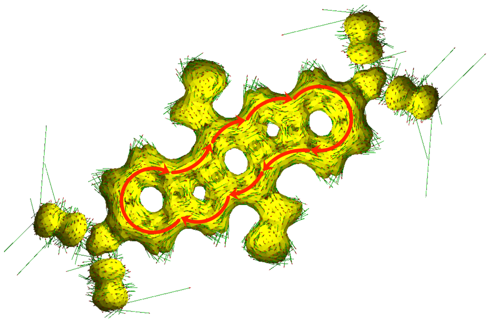

(i) PD1 S_0

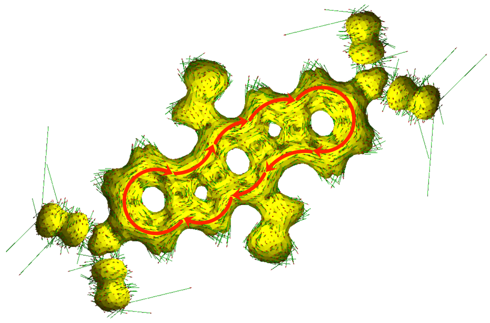

(j) PD1 T_0

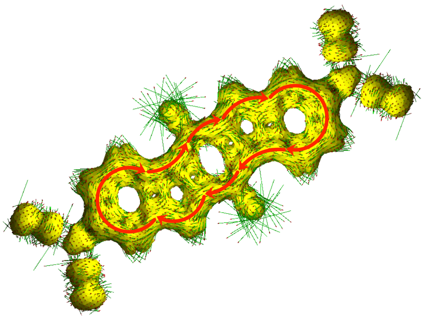

(k) PD2 S_0

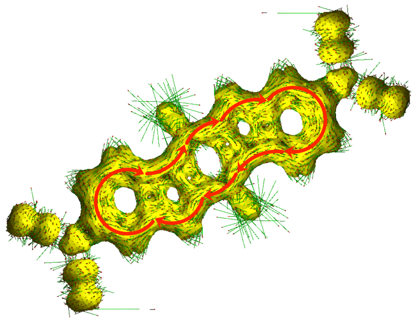

(l) PD2 T_0

ACID plots for different spin state of derivatives of **PH**. Isosurfaces with value of 0.030 (continued).

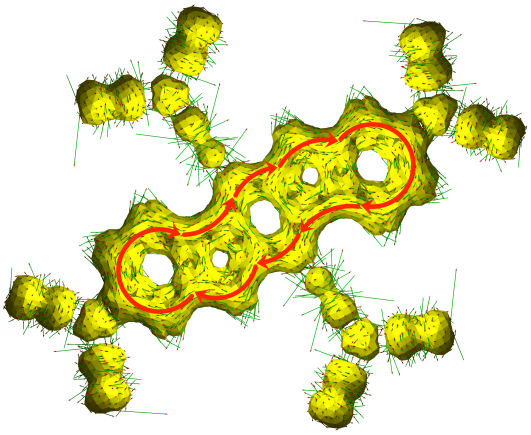

(m) **PT** S_0

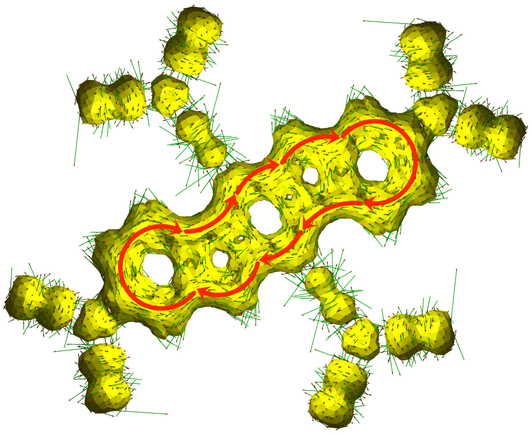

(n) **PT** T_0

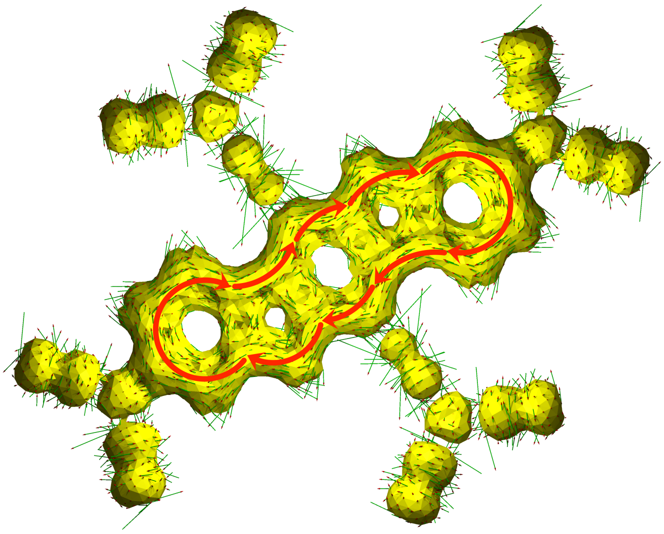

(o) **PT** Q_0

ACID plots for the different spin states of derivatives of **PH**. Isosurfaces with the value of 0.030 (continued).

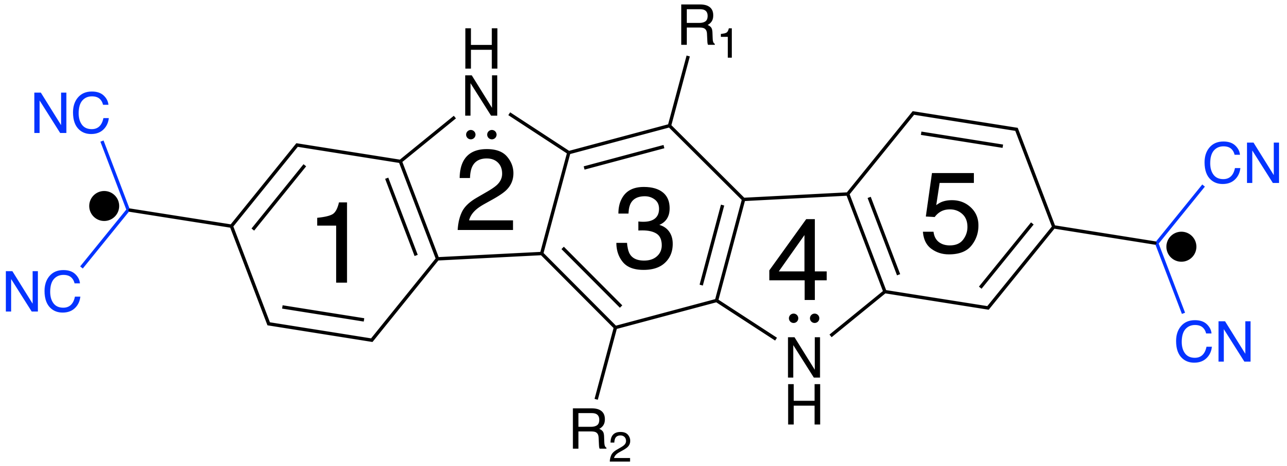

Figure S13. Ring numbering for **PH** and its derivatives.

Table S8. Multicenter index (MCI) results for Singlet (S), Triplet (T), and Quintet (Q) diradical(oid) and/or tetraradical(oid) spin states of derivatives of **PH** with different exchange-correlation functionals. Rings (R1, R2 ...) are numbered according to Figure S13. For diradical(oid)s triplet-optimized and for tetraradical(oid)s quintet-optimized DFT geometries are used.

Compound	State	Multicenter Index				
		R1	R2	R3	R4	R5
**PH**	S	0.027	0.011	0.024	0.011	0.027
**PH**	T	0.031	0.011	0.028	0.011	0.031
**PCN**	S	0.028	0.012	0.017	0.012	0.028
**PCN**	T	0.031	0.013	0.020	0.013	0.031
**PCN2**	S	0.027	0.013	0.015	0.013	0.027
**PCN2**	T	0.031	0.013	0.017	0.013	0.031
**PN1**	S	0.029	0.015	0.013	0.015	0.029
**PN1**	T	0.027	0.013	0.015	0.013	0.027
**PM1**	S	0.027	0.013	0.015	0.012	0.028
**PM1**	T	0.030	0.013	0.017	0.013	0.030
**PM2**	S	0.027	0.013	0.015	0.013	0.027
**PM2**	T	0.030	0.014	0.016	0.013	0.030
**PD**	S	0.027	0.012	0.020	0.012	0.027
**PD**	T	0.031	0.012	0.024	0.012	0.031
**PA**	S	0.030	0.019	0.005	0.019	0.030
**PA**	T	0.030	0.019	0.005	0.019	0.030
**PT**	S	0.029	0.016	0.011	0.016	0.029
**PT**	T	0.029	0.016	0.011	0.016	0.029
**PT**	Q	0.032	0.014	0.015	0.014	0.032
**PTP**	S	0.028	0.014	0.014	0.014	0.028
**PTP**	T	0.030	0.015	0.014	0.014	0.029
**PTP**	Q	0.031	0.013	0.017	0.013	0.031

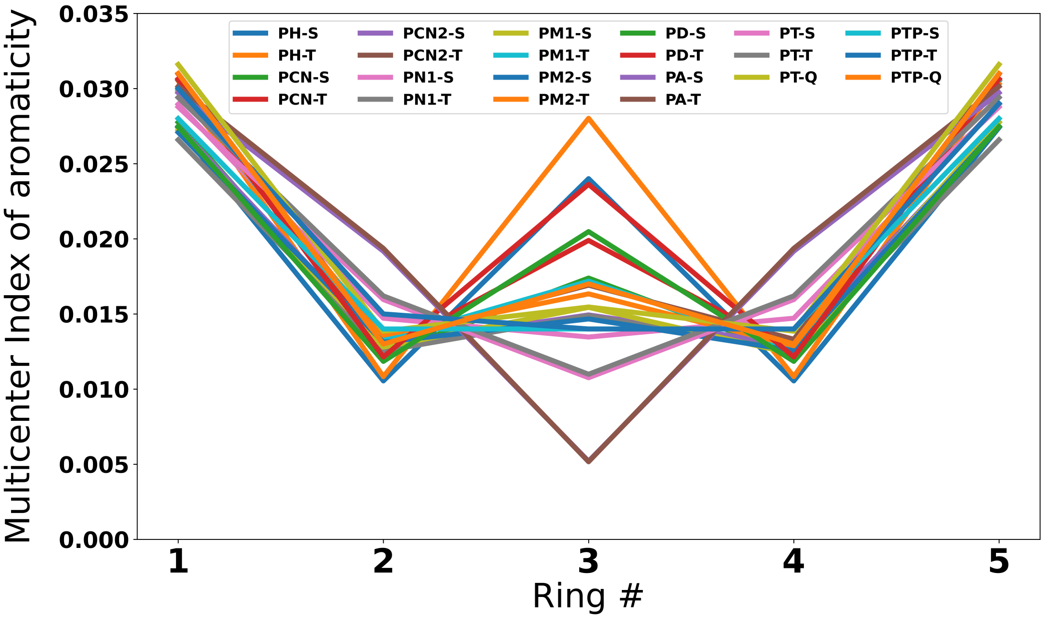

Figure S14. The graph of multicenter index (MCI) of aromaticity for several derivatives of **PH**. States are labeled as S (singlet), T (triplet), and Q (Quintet) for low-lying spin states of these diradical(oid)s and two tetraradical(oid)s **PT** and **PTP**. Ring numbering is given in Figure S13. For diradical(oid)s triplet-optimized and for tetraradical(oid)s quintet-optimized DFT geometries are used.

S6. Summary of the Electronic Structure of Tetraradical(oid) PT

By performing calculations on the electronic structure, exchange-coupling constants between radical centers, and *local* and *global* aromaticity of **PT**, we find that the molecule consists of two π-subsystems: 4 unpaired electrons and an aromatic π-subsystem. Since, according to CASSCF (4,4) and (12,12) results, the tetraradical character of **PT** is at best 22% (with 1.4% lower bound), this is only a partial structure, with another very significant contribution from the diradical configuration, which has one of the shells closed by pairing-up two electrons with the strongest coupling shown in Figure S15a. The energy spectrum of low-lying spin states of **PT** is given in Figure S15b with corresponding energy gaps for triplet- and quintet-optimized geometries.

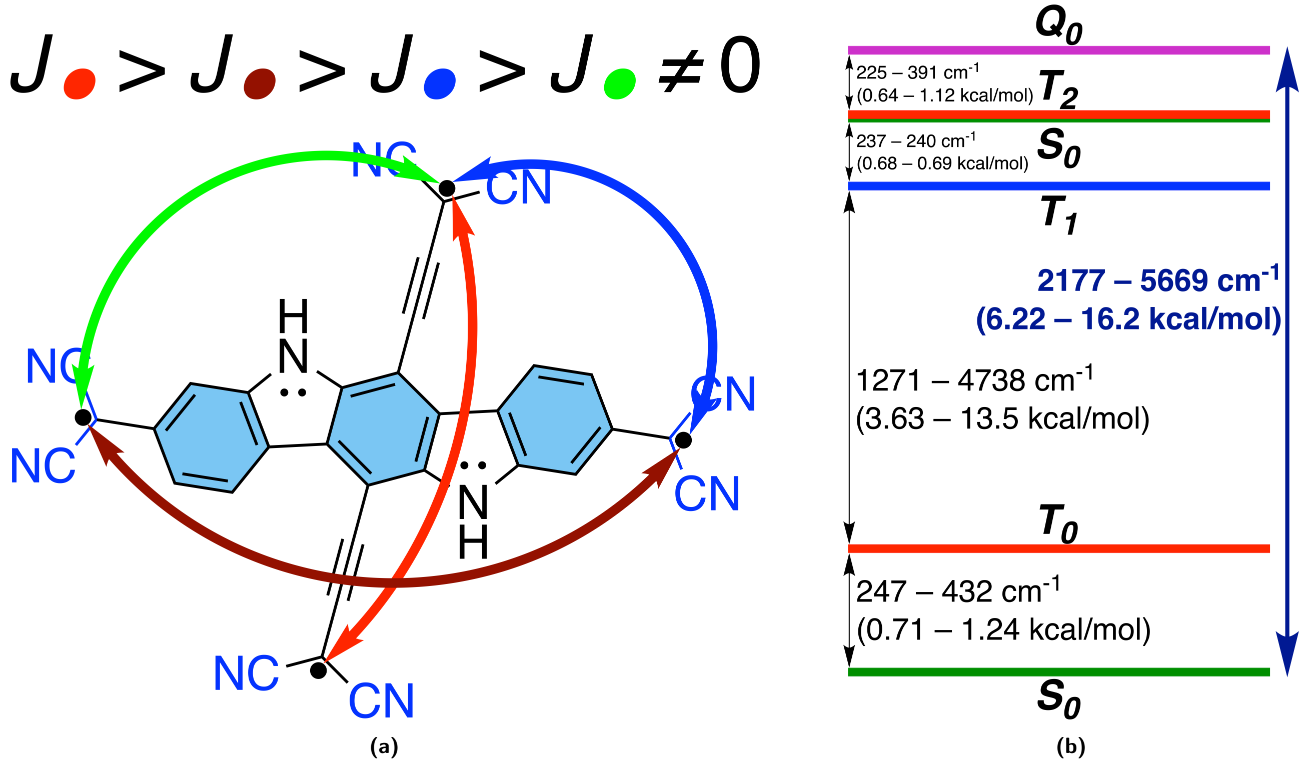

Figure S15. (a) A scheme of exchange-coupling constants and their strengths and (b) energy spectrum of low-lying spin states of **PT**.

S7. Comparison of CASSCF and CASCI Results for Triplet- and Quintet-optimized Geometries of PT

Table S9. CASSCF(4,4) states of **PT** determined by state-specific calculations. For all the calculations, UHF quintet NOs were used as initial guess. Triplet DFT-optimized geometry. Under HONO, LUNO, etc. three columns represent the symmetry of the orbital, its symbolic representation according to Figure S7 and NO occupation number.

State	Symmetry	Energy (a.u.)	CASSCF NOs symmetry, identity and occupation number.												ΔE from G. S. (cm^{-1})
			HONO − 1			HONO			LUNO			LUNO + 1			
S_0	A_g	-1836.5164127881	A_u	L_+	1.845	A_u	S_+	1.089	B_g	S_-	0.909	B_g	L_-	0.157	0.00
T_0	B_u	-1836.5158035006	A_u	L_+	1.854	A_u	S_+	1.001	B_g	S_-	0.997	B_g	L_-	0.148	133.72
T_1	B_u	-1836.4944678448	A_u	S_+	1.171	A_u	L_+	0.993	B_g	L_-	0.991	B_g	S_-	0.845	4816.36
S_1	A_g	-1836.4944336904	A_u	C_0	1.173	B_g	C_1	1.025	B_g	C_1'	0.951	A_u	C_2	0.850	4823.85
T_2	A_g	-1836.4940726388	A_u	C_0	1.122	B_g	C_1	1.061	B_g	C_1'	0.939	A_u	C_2	0.878	4903.10
Q_0	A_g	-1836.4928334987	A_u	C_0	1.000	B_g	S_-	1.000	A_u	C_2	1.000	B_g	L_-	1.000	5175.06

Since **PT** is *at least* a diradical by design, it is pertinent to compare CASSCF and CASCI results for geometries that are supposed to resemble diradical or tetraradical. We take a triplet-optimized geometry as resembling the diradical geometry of **PT** most closely and a quintet-optimized geometry as the resembling tetraradical geometry of **PT**. As we are using the monoconfigurational method DFT to optimize the geometry of **PT**, the indicated spin multiplicity will define which valence bond forms (VBFs) are allowed, and this will translate into variance in

Table S10. CASSCF(4,4) states of **PT** determined by state-specific calculations. For all the calculations, UHF quintet NOs were used as initial guess. Quintet DFT-optimized geometry. Under HONO, LUNO, etc. three columns represent the symmetry of the orbital, its symbolic representation according to Figure S7 and NO occupation number.

State	Symmetry	Energy (a.u.)	CASSCF NOs symmetry, identity and occupation number.												ΔE from G. S. (cm^{-1})
			HONO − 1			HONO			LUNO			LUNO + 1			
S_0	A_g	-1836.5108422253	A_u	L_+	1.487	A_u	S_+	1.110	B_g	S_-	0.886	B_g	L_-	0.518	0.00
T_0	B_u	-1836.5098812397	A_u	L_+	1.503	B_g	S_-	1.002	A_u	S_+	0.995	B_g	L_-	0.500	210.91
T_1	B_u	-1836.5039231981	A_u	S_+	1.142	A_u	L_+	1.004	B_g	L_-	0.987	B_g	S_-	0.868	1518.55
S_1	A_g	-1836.5035796484	A_u	C_0	1.126	B_g	C_1	1.010	B_g	$C_1^{'}$	0.967	A_u	C_2	0.898	1593.95
T_2	A_g	-1836.5034380787	A_u	C_0	1.091	B_g	C_1	1.048	B_g	$C_1^{'}$	0.952	A_u	C_2	0.909	1625.02
Q_0	A_g	-1836.5027117295	A_u	C_0	1.000	B_g	C_1	1.000	A_u	C_2	1.000	B_g	$C_1^{'}$	1.000	1784.44

Table S11. CASSCF(12,12) states determined by state-specific calculations. For all the calculations, UHF quintet NOs were used as initial guess. Triplet DFT-optimized geometry. Under HONO, LUNO, etc. three columns represent the symmetry of the orbital, its symbolic representation according to Figure S7 and NO occupation number.

State	Symmetry	Energy (a.u.)	CASSCF NOs symmetry, identity and occupation number.												ΔE from G. S. (cm^{-1})
			HONO − 1			HONO			LUNO			LUNO + 1			
S_0	A_g	-1836.6068368736	A_u	L_+	1.839	A_u	S_+	1.098	B_g	S_-	0.899	B_g	L_-	0.167	0.00
T_0	B_u	-1836.6057097226	A_u	L_+	1.857	B_g	S_-	0.999	A_u	S_+	0.999	B_g	L_-	0.148	247.38
T_1	B_u	-1836.5841212883	A_u	S_+	1.214	A_u	L_+	1.013	B_g	L_-	0.966	B_g	S_-	0.810	4985.49
S_1	A_g	-1836.5830281646	A_u	C_0	1.194	B_g	C_1	1.012	B_g	$C_1^{'}$	0.948	A_u	C_2	0.847	5225.41
T_2	A_g	-1836.5827862413	A_u	C_0	1.145	B_g	C_1	1.070	B_g	$C_1^{'}$	0.927	A_u	C_2	0.859	5278.50
Q_0	A_g	-1836.5810047998	A_u	C_0	1.005	B_g	L_-	1.000	A_u	C_2	0.998	B_g	S_-	0.996	5669.48

Table S12. CASSCF(12,12) states determined by state-specific calculations. For all the calculations, UHF quintet NOs were used as initial guess. Quintet DFT-optimized geometry. Under HONO, LUNO, etc. three columns represent the symmetry of the orbital, its symbolic representation according to Figure S7 and NO occupation number.

State	Symmetry	Energy (a.u.)	CASSCF NOs symmetry, identity and occupation number.												ΔE from G. S. (cm^{-1})
			HONO − 1			HONO			LUNO			LUNO + 1			
S_0	A_g	-1836.6003248840	A_u	L_+	1.497	A_u	S_+	1.122	B_g	S_-	0.872	B_g	L_-	0.511	0.00
T_0	B_u	-1836.5983566998	A_u	L_+	1.540	B_g	S_-	1.000	A_u	S_+	0.996	B_g	L_-	0.465	431.97
T_1	B_u	-1836.5925672155	A_u	S_+	1.154	A_u	L_+	1.033	B_g	L_-	0.957	B_g	S_-	0.858	1702.61
S_1	A_g	-1836.5914852556	A_u	C_0	1.140	B_g	C_1	1.001	B_g	$C_1^{'}$	0.963	A_u	C_2	0.897	1940.07
T_2	A_g	-1836.5914276798	A_u	C_0	1.106	B_g	C_1	1.054	B_g	$C_1^{'}$	0.943	A_u	C_2	0.898	1952.71
Q_0	A_g	-1836.5904040182	A_u	C_0	1.005	B_g	L_-	1.000	A_u	C_2	0.999	B_g	S_-	0.997	2177.38

Table S13. CASCI(4,4) for **PT** from CASSCF(12,12) quintet converged orbitals as initial guess. Triplet DFT-optimized geometry. Under HONO, LUNO, etc. three columns represent the symmetry of the orbital, its symbolic representation according to Figure S7 and NO occupation number.

State	Symmetry	Energy (a.u.)	CASCI NOs occupancy, identity and symmetry												ΔE from G. S. (cm^{-1})
			HONO − 1			HONO			LUNO			LUNO + 1			
S_0	A	-1836.4905063983	A	L_+	1.388	A	S_+	1.120	A	S_-	0.871	A	L_-	0.621	0.00
T_0	A	-1836.4883811269	A	L_+	1.366	A	S_-	1.003	A	S_+	0.990	A	L_-	0.641	466.44
T_1	A	-1836.4819232909	A	S_+	1.204	A	L_+	0.999	A	L_-	0.982	A	S_-	0.815	1883.77
S_1	A	-1836.4808958271	A	C_0	1.194	A	C_1	1.024	A	$C_1^{'}$	0.941	A	C_2	0.840	2109.28
T_2	A	-1836.4808259676	A	C_0	1.145	A	C_1	1.068	A	$C_1^{'}$	0.932	A	C_2	0.855	2124.61
Q_0	A	-1836.4794440226	A	D_0[a]	1.000	A	D_1	1.000	A	D_2	1.000	A	D_3	1.000	2427.91

[a] D_0, D_1, D_2, D_3 means **distorted** orbitals with the same overall electron density as from C_0, C_1, $C_1^{'}$, C_2 orbitals.

optimized geometries, as exemplified in Figure S10. By referring to Figure S6, we can reason that an optimized geometry for a quintet state will resemble the VBF of the tetraradical only, which implies bias towards tetraradical states and bias against diradical states. Conversely, when we optimize geometry for a triplet state, we allow all diradical and tetraradical VBFs to be mixed in the resulting structure, which by default means a bias against tetraradical states and bias towards diradical states because there are much more diradical VBFs than tetraradical VBFs for **PT**. Since it has been shown that **PT** possesses some tetraradical character, and is at least a diradical, the real results should fall between extremes of results from triplet-optimized and quintet-optimized geometries.

Results given in Table S9 to Table S16 compare CASSCF(4,4), CASSCF(12,12), CASCI(4,4) and CASCI(12,12) for triplet- and quintet-optimized geometries. Notably, for triplet-optimized geometry, according to CASSCF(4,4) and CASSCF(12,12), S_0 states imply very small tetraradical character, y_1 = 0.014–0.016, while for quintet-

Table S14. CASCI(4,4) for **PT** from CASSCF(12,12) quintet converged orbitals as initial guess. Quintet DFT-optimized geometry. Under HONO, LUNO, etc. three columns represent the symmetry of the orbital, its symbolic representation according to Figure S7 and NO occupation number.

State	Symmetry	Energy (a.u.)	CASCI NOs occupancy, identity and symmetry												ΔE from G. S. (cm^{-1})
			HONO − 1			HONO			LUNO			LUNO + 1			
S_0	A	-1836.4954786613	A	C_0	1.285	A	C_2	1.110	A	C_1	0.893	A	C_1'	0.722	0.00
T_0	A	-1836.4936832384	A	L_+	1.238	A	S_+	0.999	A	S_-	0.997	A	L_-	0.767	394.05
T_1	A	-1836.4911158413	A	S_+	1.166	A	L_+	1.021	A	L_-	0.969	A	S_-	0.843	957.53
T_2	A	-1836.4900247041	A	C_0	1.110	A	C_1	1.054	A	C_1'	0.946	A	C_2	0.890	1197.01
S_1	A	-1836.4899838313	A	C_0	1.146	A	C_1	1.012	A	C_1'	0.956	A	C_2	0.886	1205.98
Q_0	A	-1836.4892522623	A	D_0[a]	1.000	A	D_1	1.000	A	D_2	1.000	A	D_3	1.000	1366.54

[a] D_0, D_1, D_2, D_3 means **distorted** orbitals with the same overall electron density as from C_0, C_1, C_1', C_2 orbitals.

Table S15. CASCI(12,12) for **PT** from CASSCF(12,12) quintet converged orbitals as initial guess. Triplet DFT-optimized geometry. Under HONO, LUNO, etc. three columns represent the symmetry of the orbital, its symbolic representation according to Figure S7 and NO occupation number.

State	Symmetry	Energy (a.u.)	CASCI NOs occupancy, identity and symmetry.												ΔE from G. S. (cm^{-1})
			HONO − 1			HONO			LUNO			LUNO + 1			
S_0	A	-1836.5918472899	A	L_+	1.372	A	S_+	1.091	A	S_-	0.902	A	L_-	0.621	0.00
T_0	A	-1836.5899303437	A	L_+	1.363	A	S_-	1.000	A	S_+	0.995	A	L_-	0.641	420.72
T_1	A	-1836.5833385682	A	S_+	1.158	A	L_+	0.995	A	L_-	0.985	A	S_-	0.815	1867.45
S_1	A	-1836.5825912324	A	C_0	1.165	A	C_1	1.025	A	C_1'	0.943	A	C_2	0.840	2031.47
T_2	A	-1836.5824474708	A	C_0	1.125	A	C_1	1.060	A	C_1'	0.937	A	C_2	0.855	2063.02
Q_0	A	-1836.5810047797	A	C_0	1.005	A	L_-	1.000	A	C_2	0.998	A	S_-	1.000	2379.66

Table S16. CASCI(12,12) for **PT** from CASSCF(12,12) quintet converged orbitals as initial guess. Quintet DFT-optimized geometry. Under HONO, LUNO, etc. three columns represent the symmetry of the orbital, its symbolic representation according to Figure S7 and NO occupation number.

State	Symmetry	Energy (a.u.)	CASCI NOs occupancy, identity and symmetry.												ΔE from G. S. (cm^{-1})
			HONO − 1			HONO			LUNO			LUNO + 1			
S_0	A	-1836.5964215286	A	C_0	1.263	A	C_2	1.077	A	C_1	0.918	A	C_1'	0.744	0.00
T_0	A	-1836.5948052330	A	L_+	1.236	A	S_+	0.999	A	S_-	0.997	A	L_-	0.768	354.74
T_1	A	-1836.5921396709	A	S_+	1.128	A	L_+	1.013	A	L_-	0.975	A	S_-	0.885	939.76
S_1	A	-1836.5912545906	A	C_0	1.122	A	C_1	1.012	A	C_1'	0.958	A	C_2	0.908	1134.01
T_2	A	-1836.5912370713	A	C_0	1.094	A	C_1	1.046	A	C_1'	0.951	A	C_2	0.910	1137.86
Q_0	A	-1836.5904039925	A	C_0	1.005	A	L_-	1.000	A	C_2	0.999	A	S_-	0.995	1320.70

optimized geometries, the results imply much higher tetraradical character, $y_1 = 0.21$–0.22, while diradical characters change from $y_0 = 0.80$–0.82 for triplet-optimized geometry to $y_0 = 0.75$–0.78 for quintet-optimized geometry. Moreover, CASSCF(12,12) results show that the energy gap between T_0 and T_1 states ($\Delta E_{T_0-T_1}$) is 4738 cm^{-1} and the spectral range for the six spin-states in the low-energy spectrum is 5669 cm^{-1} for triplet-optimized geometry, while for quintet-optimized geometry $\Delta E_{T_0-T_1} = 1271$ cm^{-1} and the spectral range is 2177 cm^{-1}.

S8. Optimized Geometries

The closed-shell singlet-optimized C_{2h} geometry of molecule **PH** is given below (BLYP/TZP level of theory). Total bonding energy: -10.322358435993245 $a.u.$

```
40
C24H10N6 optimized with ADF in AMS.
C       -3.28932983555643       0.73453199901555       0.00000000000000
C       -2.73776758059487      -0.60103680369406       0.00000000000000
N       -2.22759055295166       1.63841074376692       0.00000000000000
C       -4.64717811826357       0.97681320180130       0.00000000000000
C       -3.63237981621405      -1.70686329262708       0.00000000000000
C       -4.99039767377712      -1.48475703730038       0.00000000000000
C       -5.53801403686675      -0.14557678939623       0.00000000000000
N        8.59818150103390       1.94754719447538       0.00000000000000
```

```
H       -5.04742468663157       1.98885027294210       0.00000000000000
H        3.24814780960379       2.72576556168529       0.00000000000000
C       -1.32309181212508      -0.48149681209836       0.00000000000000
H       -3.24814780960379      -2.72576556168529       0.00000000000000
H       -5.67950687703697      -2.32610274040767       0.00000000000000
C       -1.01749377059353       0.94582153126793       0.00000000000000
C        0.27613380069447       1.43699721892214       0.00000000000000
C        1.32309181212508       0.48149681209836       0.00000000000000
C        1.01749377059353      -0.94582153126793       0.00000000000000
C       -0.27613380069447      -1.43699721892214       0.00000000000000
H       -0.48670869923928      -2.50493068308227       0.00000000000000
H        0.48670869923928       2.50493068308227       0.00000000000000
C        2.73776758059487       0.60103680369406       0.00000000000000
C        3.28932983555643      -0.73453199901555       0.00000000000000
N        2.22759055295166      -1.63841074376692       0.00000000000000
N        7.94600950683144      -2.44474633644683       0.00000000000000
C        3.63237981621405       1.70686329262708       0.00000000000000
C        4.99039767377712       1.48475703730038       0.00000000000000
C        5.53801403686675       0.14557678939623       0.00000000000000
C        4.64717811826357      -0.97681320180130       0.00000000000000
N       -7.94600950683144       2.44474633644683       0.00000000000000
H        5.67950687703697       2.32610274040767       0.00000000000000
H        5.04742468663157      -1.98885027294210       0.00000000000000
C       -6.94988804141580       0.05220572623322       0.00000000000000
C       -7.85965035722390      -1.03949335136698       0.00000000000000
C       -7.51226251515986       1.35756125358201       0.00000000000000
N       -8.59818150103390      -1.94754719447538       0.00000000000000
C        6.94988804141580      -0.05220572623322       0.00000000000000
C        7.85965035722390       1.03949335136698       0.00000000000000
C        7.51226251515986      -1.35756125358201       0.00000000000000
H       -2.32239866788273       2.64740958182783       0.00000000000000
H        2.32239866788273      -2.64740958182783       0.00000000000000
```

The triplet-optimized C_{2h} geometry of molecule **PH** is given below (BLYP/TZP level of theory).
Total bonding energy: -10.315426748220721$a.u.$

```
40
C24H10N6 optimized with ADF in AMS.
C       -3.29682143948277       0.72118054188573       0.00000000000000
C       -2.75584592773433      -0.60918306904103       0.00000000000000
N       -2.23836800106932       1.62554665586360       0.00000000000000
C       -4.66474222541586       0.96461981587275       0.00000000000000
C       -3.64359015512497      -1.70771432844440       0.00000000000000
C       -5.01083319380143      -1.48351697643797       0.00000000000000
C       -5.54702647201785      -0.15171330629033       0.00000000000000
N        8.62871969719202       1.93946075704300       0.00000000000000
H       -5.06195187062772       1.97802061323854       0.00000000000000
H        3.26236877299436       2.72776359127281       0.00000000000000
C       -1.31831417184317      -0.48394518469552       0.00000000000000
H       -3.26236877299436      -2.72776359127281       0.00000000000000
H       -5.69840925491024      -2.32611149815264       0.00000000000000
C       -1.02509955197532       0.93121990509070       0.00000000000000
C        0.27556012459595       1.43040909075843       0.00000000000000
C        1.31831417184317       0.48394518469552       0.00000000000000
C        1.02509955197532      -0.93121990509070       0.00000000000000
C       -0.27556012459595      -1.43040909075843       0.00000000000000
H       -0.47955787050839      -2.49987473509340       0.00000000000000
H        0.47955787050839       2.49987473509340       0.00000000000000
```

```
C        2.75584592773433       0.60918306904103       0.00000000000000
C        3.29682143948277      -0.72118054188573       0.00000000000000
N        2.23836800106932      -1.62554665586360       0.00000000000000
N        7.96449308570473      -2.44954603444705       0.00000000000000
C        3.64359015512497       1.70771432844440       0.00000000000000
C        5.01083319380143       1.48351697643797       0.00000000000000
C        5.54702647201785       0.15171330629033       0.00000000000000
C        4.66474222541586      -0.96461981587275       0.00000000000000
N       -7.96449308570473       2.44954603444705       0.00000000000000
H        5.69840925491024       2.32611149815264       0.00000000000000
H        5.06195187062772      -1.97802061323854       0.00000000000000
C       -6.97894554984883       0.05547587967166       0.00000000000000
C       -7.88891616805757      -1.03108486658144       0.00000000000000
C       -7.53458292218716       1.35977542883174       0.00000000000000
N       -8.62871969719202      -1.93946075704300       0.00000000000000
C        6.97894554984883      -0.05547587967166       0.00000000000000
C        7.88891616805757       1.03108486658144       0.00000000000000
C        7.53458292218716      -1.35977542883174       0.00000000000000
H       -2.33397447152207       2.63426412183599       0.00000000000000
H        2.33397447152207      -2.63426412183599       0.00000000000000
```

The closed-shell singlet-optimized C_{2h} geometry of molecule **PH1** is given below (BLYP/TZP level of theory). Total bonding energy: -11.429983486172727 $a.u.$

```
44
C28H10N6 optimized with ADF in AMS.
C       3.28236079155494        0.77086866669358        0.00000000000000
C       2.75556398073554        -0.57604466856377       0.00000000000000
N       2.20247492225071        1.65256094619377        0.00000000000000
C       4.63323285555941        1.04462731856467        0.00000000000000
C       3.67682368144107        -1.66276406468587       0.00000000000000
C       5.02883303421463        -1.40775476751995       0.00000000000000
C       5.54847436582193        -0.05813337045215       0.00000000000000
N       -8.65096698471763       1.78807518476151        0.00000000000000
H       5.00910858181356        2.06594945439293        0.00000000000000
H       -3.31114389209992       2.68617287360394        0.00000000000000
C       1.33904635234329        -0.47811711737226       0.00000000000000
H       3.31114389209992        -2.68617287360394       0.00000000000000
H       5.73596745831903        -2.23417380722990       0.00000000000000
C       1.01610203784644        0.93810457782250        0.00000000000000
C       -0.28755218153778       1.45208771953389        0.00000000000000
C       -1.33904635234329       0.47811711737226        0.00000000000000
C       -1.01610203784644       -0.93810457782250       0.00000000000000
C       0.28755218153778        -1.45208771953389       0.00000000000000
H       2.25766931413495        2.66575173106475        0.00000000000000
H       -2.25766931413495       -2.66575173106475       0.00000000000000
C       -2.75556398073554       0.57604466856377        0.00000000000000
C       -3.28236079155494       -0.77086866669358       0.00000000000000
N       -2.20247492225071       -1.65256094619377       0.00000000000000
N       -7.90066685671256       -2.58496433014708       0.00000000000000
C       -3.67682368144107       1.66276406468587        0.00000000000000
C       -5.02883303421463       1.40775476751995        0.00000000000000
C       -5.54847436582193       0.05813337045215        0.00000000000000
C       -4.63323285555941       -1.04462731856467       0.00000000000000
N       7.90066685671256        2.58496433014708        0.00000000000000
H       -5.73596745831903       2.23417380722990        0.00000000000000
H       -5.00910858181356       -2.06594945439293       0.00000000000000
C       6.95500655575747        0.17172959282316        0.00000000000000
```

C	7.89008843480016	-0.89870429781584	0.00000000000000
C	7.48889301787084	1.48928145995733	0.00000000000000
N	8.65096698471763	-1.78807518476151	0.00000000000000
C	-6.95500655575747	-0.17172959282316	0.00000000000000
C	-7.89008843480016	0.89870429781584	0.00000000000000
C	-7.48889301787084	-1.48928145995733	0.00000000000000
C	0.50762263667663	-2.85546558525020	0.00000000000000
C	-0.50762263667663	2.85546558525020	0.00000000000000
C	0.63015553806627	-4.06440002389122	0.00000000000000
C	-0.63015553806627	4.06440002389122	0.00000000000000
H	-0.76241698069224	5.12530765194672	0.00000000000000
H	0.76241698069224	-5.12530765194672	0.00000000000000

The triplet-optimized C_{2h} geometry of molecule **PH1** is given below (BLYP/TZP level of theory).
Total bonding energy: -11.423780349368114 *a.u.*

44
C28H10N6 optimized with ADF in AMS.

C	3.28992567886860	0.76053688359150	0.00000000000000
C	2.77432079400913	-0.58121815450606	0.00000000000000
N	2.21289545827639	1.64238042567592	0.00000000000000
C	4.65123777907925	1.03631458171327	0.00000000000000
C	3.68827556822849	-1.66115761629486	0.00000000000000
C	5.04909464137886	-1.40343697421535	0.00000000000000
C	5.55722824014797	-0.06044360498087	0.00000000000000
N	-8.67814199026212	1.78058538337941	0.00000000000000
H	5.02462634278635	2.05888419895227	0.00000000000000
H	-3.32547565643549	2.68585117593693	0.00000000000000
C	1.33519293969773	-0.47896293187671	0.00000000000000
H	3.32547565643549	-2.68585117593693	0.00000000000000
H	5.75588762116427	-2.23046397602149	0.00000000000000
C	1.02374048012004	0.92551339666143	0.00000000000000
C	-0.28870517633960	1.44428563938031	0.00000000000000
C	-1.33519293969773	0.47896293187671	0.00000000000000
C	-1.02374048012004	-0.92551339666143	0.00000000000000
C	0.28870517633960	-1.44428563938031	0.00000000000000
H	2.26847830451175	2.65542423486241	0.00000000000000
H	-2.26847830451175	-2.65542423486241	0.00000000000000
C	-2.77432079400913	0.58121815450606	0.00000000000000
C	-3.28992567886860	-0.76053688359150	0.00000000000000
N	-2.21289545827639	-1.64238042567592	0.00000000000000
N	-7.91796086139214	-2.59228820317927	0.00000000000000
C	-3.68827556822849	1.66115761629486	0.00000000000000
C	-5.04909464137886	1.40343697421535	0.00000000000000
C	-5.55722824014797	0.06044360498087	0.00000000000000
C	-4.65123777907925	-1.03631458171327	0.00000000000000
N	7.91796086139214	2.59228820317927	0.00000000000000
H	-5.75588762116427	2.23046397602149	0.00000000000000
H	-5.02462634278635	-2.05888419895227	0.00000000000000
C	6.98377315047508	0.17722610004495	0.00000000000000
C	7.91742691929575	-0.88945976994339	0.00000000000000
C	7.51212762825352	1.49328662608201	0.00000000000000
N	8.67814199026212	-1.78058538337941	0.00000000000000
C	-6.98377315047508	-0.17722610004495	0.00000000000000
C	-7.91742691929575	0.88945976994339	0.00000000000000
C	-7.51212762825352	-1.49328662608201	0.00000000000000
C	0.50262086083042	-2.84868076620269	0.00000000000000
C	-0.50262086083042	2.84868076620269	0.00000000000000

```
C       0.61770207927313        -4.05831005062405        0.00000000000000
C       -0.61770207927313       4.05831005062405         0.00000000000000
H       -0.74506987008248       5.11984739670003         0.00000000000000
H       0.74506987008248        -5.11984739670003        0.00000000000000
```

The closed-shell singlet-optimized C_{2h} geometry of molecule **PH2** is given below (BLYP/TZP level of theory). Total bonding energy: -12.560674625269501 $a.u.$

```
48
C32H10N6 optimized with ADF in AMS.
C        3.28473400267099       0.76895363860594       0.00000000000000
C        2.75594515031305      -0.57725058801976       0.00000000000000
N        2.20534773297187       1.65137968006142       0.00000000000000
C        4.63567993375438       1.04147608473919       0.00000000000000
C        3.67578248779899      -1.66558173949709       0.00000000000000
C        5.02776210567410      -1.41178696727090       0.00000000000000
C        5.54921667497100      -0.06264271757755       0.00000000000000
N       -8.64931139144932       1.79692409200358       0.00000000000000
H        5.01259226772561       2.06234065626591       0.00000000000000
H       -3.31111763958048       2.68935569150848       0.00000000000000
C        1.34053053708517      -0.47632578037240       0.00000000000000
H        3.31111763958048      -2.68935569150848       0.00000000000000
H        5.73365107528126      -2.23916903980552       0.00000000000000
C        1.01934613467760       0.93747294266402       0.00000000000000
C       -0.28731274792907       1.45502371908809       0.00000000000000
C       -1.34053053708517       0.47632578037240       0.00000000000000
C       -1.01934613467760      -0.93747294266402       0.00000000000000
C        0.28731274792907      -1.45502371908809       0.00000000000000
H        2.26296002481776       2.66444583673660       0.00000000000000
H       -2.26296002481776      -2.66444583673660       0.00000000000000
C       -2.75594515031305       0.57725058801976       0.00000000000000
C       -3.28473400267099      -0.76895363860594       0.00000000000000
N       -2.20534773297187      -1.65137968006142       0.00000000000000
N       -7.90574251308752      -2.57628433484800       0.00000000000000
C       -3.67578248779899       1.66558173949709       0.00000000000000
C       -5.02776210567410       1.41178696727090       0.00000000000000
C       -5.54921667497100       0.06264271757755       0.00000000000000
C       -4.63567993375438      -1.04147608473919       0.00000000000000
N        7.90574251308752       2.57628433484800       0.00000000000000
H       -5.73365107528126       2.23916903980552       0.00000000000000
H       -5.01259226772561      -2.06234065626591       0.00000000000000
C        6.95589008280397       0.16487859053615       0.00000000000000
C        7.88938004865011      -0.90687854050264       0.00000000000000
C        7.49195031563749       1.48150080420268       0.00000000000000
N        8.64931139144932      -1.79692409200358       0.00000000000000
C       -6.95589008280397      -0.16487859053615       0.00000000000000
C       -7.88938004865011       0.90687854050264       0.00000000000000
C       -7.49195031563749      -1.48150080420268       0.00000000000000
C        0.51103144612970      -2.84722871475795       0.00000000000000
C       -0.51103144612970       2.84722871475795       0.00000000000000
C        0.64333436154832      -4.06805032973023       0.00000000000000
C       -0.64333436154832       4.06805032973023       0.00000000000000
C        0.81294163630147      -5.41449650049100       0.00000000000000
C        0.96465686385895      -6.62446077232409       0.00000000000000
C       -0.81294163630147       5.41449650049100       0.00000000000000
C       -0.96465686385895       6.62446077232409       0.00000000000000
H       -1.09849574474187       7.68478708270425       0.00000000000000
H        1.09849574474187      -7.68478708270425       0.00000000000000
```

The triplet-optimized C_{2h} geometry of molecule **PH1** is given below (BLYP/TZP level of theory).
Total bonding energy: -12.554663360465186 *a.u.*

```
48
C32H10N6 optimized with ADF in AMS.
C        3.29118484772453       0.76117969176311       0.00000000000000
C        2.77475483405666      -0.57973773729402       0.00000000000000
N        2.21338215230005       1.64293484295740       0.00000000000000
C        4.65173307875495       1.03721764505536       0.00000000000000
C        3.68867801540577      -1.66011020846369       0.00000000000000
C        5.04915599752899      -1.40210885877140       0.00000000000000
C        5.55798376978639      -0.05958687001810       0.00000000000000
N       -8.67924947648367       1.77844699431284       0.00000000000000
H        5.02409646145517       2.05988280851455       0.00000000000000
H       -3.32865727929835       2.68563182641505       0.00000000000000
C        1.33715857695077      -0.47626637991261       0.00000000000000
H        3.32865727929835      -2.68563182641505       0.00000000000000
H        5.75474030460612      -2.22975011681950       0.00000000000000
C        1.02584503517991       0.92575525235843       0.00000000000000
C       -0.29021437910587       1.44736622537052       0.00000000000000
C       -1.33715857695077       0.47626637991261       0.00000000000000
C       -1.02584503517991      -0.92575525235843       0.00000000000000
C        0.29021437910587      -1.44736622537052       0.00000000000000
H        2.27053981893327       2.65578544464779       0.00000000000000
H       -2.27053981893327      -2.65578544464779       0.00000000000000
C       -2.77475483405666       0.57973773729402       0.00000000000000
C       -3.29118484772453      -0.76117969176311       0.00000000000000
N       -2.21338215230005      -1.64293484295740       0.00000000000000
N       -7.91949563432804      -2.59271304446996       0.00000000000000
C       -3.68867801540577       1.66011020846369       0.00000000000000
C       -5.04915599752899       1.40210885877140       0.00000000000000
C       -5.55798376978639       0.05958687001810       0.00000000000000
C       -4.65173307875495      -1.03721764505536       0.00000000000000
N        7.91949563432804       2.59271304446996       0.00000000000000
H       -5.75474030460612       2.22975011681950       0.00000000000000
H       -5.02409646145517      -2.05988280851455       0.00000000000000
C        6.98437094098557       0.17841729446868       0.00000000000000
C        7.91827454792889      -0.88786346511883       0.00000000000000
C        7.51208845503991       1.49445209953016       0.00000000000000
N        8.67924947648367      -1.77844699431284       0.00000000000000
C       -6.98437094098557      -0.17841729446868       0.00000000000000
C       -7.91827454792889       0.88786346511883       0.00000000000000
C       -7.51208845503991      -1.49445209953016       0.00000000000000
C        0.50782793832091      -2.84014442024594       0.00000000000000
C       -0.50782793832091       2.84014442024594       0.00000000000000
C        0.63239834192033      -4.06199234073799       0.00000000000000
C       -0.63239834192033       4.06199234073799       0.00000000000000
C        0.79414669381792      -5.40921253865243       0.00000000000000
C        0.93844858461621      -6.62021034027609       0.00000000000000
C       -0.79414669381792       5.40921253865243       0.00000000000000
C       -0.93844858461621       6.62021034027609       0.00000000000000
H       -1.06594617292059       7.68128120795268       0.00000000000000
H        1.06594617292059      -7.68128120795268       0.00000000000000
```

The closed-shell singlet-optimized C_{2h} geometry of molecule **PCN** is given below (BLYP/TZP level of theory).
Total bonding energy: -11.203110301072069 *a.u.*

```
42
```

```
C26H8N8 optimized with ADF in AMS.
C       3.28876455000000        0.76625322000000         0.00000000000000
C       2.76036683000000        -0.57872606000000        0.00000000000000
N       2.20817464000000        1.65359007000000         0.00000000000000
C       4.63836628000000        1.03842282000000         0.00000000000000
C       3.67672868000000        -1.67043255000000        0.00000000000000
C       5.02860788000000        -1.41756198000000        0.00000000000000
C       5.55079883000000        -0.06864115000000        0.00000000000000
N       -8.65266560000000       1.79979411000000         0.00000000000000
H       5.01647128000000        2.05888632000000         0.00000000000000
H       -3.31285009000000       2.69538158000000         0.00000000000000
C       1.34561925000000        -0.47528476000000        0.00000000000000
H       3.31285009000000        -2.69538158000000        0.00000000000000
H       5.73337396000000        -2.24594185000000        0.00000000000000
C       1.02503307000000        0.94157063000000         0.00000000000000
C       -0.28625135000000       1.43627054000000         0.00000000000000
C       -1.34561925000000       0.47528476000000         0.00000000000000
C       -1.02503307000000       -0.94157063000000        0.00000000000000
C       0.28625135000000        -1.43627054000000        0.00000000000000
H       2.27327068000000        2.66710833000000         0.00000000000000
H       -2.27327068000000       -2.66710833000000        0.00000000000000
C       -2.76036683000000       0.57872606000000         0.00000000000000
C       -3.28876455000000       -0.76625322000000        0.00000000000000
N       -2.20817464000000       -1.65359007000000        0.00000000000000
N       -7.90784954000000       -2.57014345000000        0.00000000000000
C       -3.67672868000000       1.67043255000000         0.00000000000000
C       -5.02860788000000       1.41756198000000         0.00000000000000
C       -5.55079883000000       0.06864115000000         0.00000000000000
C       -4.63836628000000       -1.03842282000000        0.00000000000000
N       7.90784954000000        2.57014345000000         0.00000000000000
H       -5.73337396000000       2.24594185000000         0.00000000000000
H       -5.01647128000000       -2.05888632000000        0.00000000000000
C       6.95586445000000        0.15929375000000         0.00000000000000
C       7.89055767000000        -0.91196048000000        0.00000000000000
C       7.49250116000000        1.47622381000000         0.00000000000000
N       8.65266560000000        -1.79979411000000        0.00000000000000
C       -6.95586445000000       -0.15929375000000        0.00000000000000
C       -7.89055767000000       0.91196048000000         0.00000000000000
C       -7.49250116000000       -1.47622381000000        0.00000000000000
C       0.51724554000000        -2.84164367000000        0.00000000000000
C       -0.51724554000000       2.84164367000000         0.00000000000000
N       0.65765913000000        -4.00160517000000        0.00000000000000
N       -0.65765913000000       4.00160517000000         0.00000000000000
```

The triplet-optimized C_{2h} geometry of molecule **PCN** is given below (BLYP/TZP level of theory).
Total bonding energy: -11.197047801307546 *a.u.*

```
42
C26H8N8 optimized with ADF in AMS.
C        3.29547606372133       0.75503407895049        0.00000000000000
C        2.77867260689919      -0.58358830840048        0.00000000000000
N        2.21874484494423       1.64328811855508        0.00000000000000
C        4.65654424439718       1.02856712793211        0.00000000000000
C        3.68668947588700      -1.66849958826432        0.00000000000000
C        5.04768740761583      -1.41269033911128        0.00000000000000
C        5.55849208037564      -0.07138560999593        0.00000000000000
N       -8.67652404604145       1.79660904536771        0.00000000000000
H        5.03133708922880       2.05027810406455        0.00000000000000
```

```
H       -3.32622490077110       2.69459653913539       0.00000000000000
C        1.34063610688972      -0.47536427229231       0.00000000000000
H        3.32622490077110      -2.69459653913539       0.00000000000000
H        5.75098754435150      -2.24220710542626       0.00000000000000
C        1.03189096608993       0.92963871489338       0.00000000000000
C       -0.28735153583604       1.42797879346058       0.00000000000000
C       -1.34063610688972       0.47536427229231       0.00000000000000
C       -1.03189096608993      -0.92963871489338       0.00000000000000
C        0.28735153583604      -1.42797879346058       0.00000000000000
H        2.28535676572840       2.65629798741514       0.00000000000000
H       -2.28535676572840      -2.65629798741514       0.00000000000000
C       -2.77867260689919       0.58358830840048       0.00000000000000
C       -3.29547606372133      -0.75503407895049       0.00000000000000
N       -2.21874484494423      -1.64328811855508       0.00000000000000
N       -7.92470464530492      -2.57643221236262       0.00000000000000
C       -3.68668947588700       1.66849958826432       0.00000000000000
C       -5.04768740761583       1.41269033911128       0.00000000000000
C       -5.55849208037564       0.07138560999593       0.00000000000000
C       -4.65654424439718      -1.02856712793211       0.00000000000000
N        7.92470464530492       2.57643221236262       0.00000000000000
H       -5.75098754435150       2.24220710542626       0.00000000000000
H       -5.03133708922880      -2.05027810406455       0.00000000000000
C        6.98634118348676       0.16376346800734       0.00000000000000
C        7.91748931590491      -0.90451101420270       0.00000000000000
C        7.51611639430043       1.47874984193577       0.00000000000000
N        8.67652404604145      -1.79660904536771       0.00000000000000
C       -6.98634118348676      -0.16376346800734       0.00000000000000
C       -7.91748931590491       0.90451101420270       0.00000000000000
C       -7.51611639430043      -1.47874984193577       0.00000000000000
C        0.51463161732170      -2.83505525870250       0.00000000000000
C       -0.51463161732170       2.83505525870250       0.00000000000000
N        0.64930989774123      -3.99541790905976       0.00000000000000
N       -0.64930989774123       3.99541790905976       0.00000000000000
```

The closed-shell singlet-optimized C_{2h} geometry of molecule **PCN1** is given below (BLYP/TZP level of theory). Total bonding energy: -12.322465817742092 $a.u.$

```
46
C30H8N8 optimized with ADF in AMS.
C       3.28840682258722        0.76634637926508        0.00000000000000
C       2.76059560022911        -0.57914234080019       0.00000000000000
N       2.20685750706933        1.65009439435554        0.00000000000000
C       4.63742615302846        1.04238953597673        0.00000000000000
C       3.68249731260218        -1.66642875536126       0.00000000000000
C       5.03384314895605        -1.41108462943945       0.00000000000000
C       5.55328806921529        -0.06123883803971       0.00000000000000
N       -8.65821982400693       1.78569915882459        0.00000000000000
H       5.01245548684668        2.06403167892317        0.00000000000000
H       -3.32256324684758       2.69193943350321        0.00000000000000
C       1.34533244262240        -0.47742818824955       0.00000000000000
H       3.32256324684758        -2.69193943350321       0.00000000000000
H       5.74040074770254        -2.23796313739927       0.00000000000000
C       1.02403775273833        0.93718165646021        0.00000000000000
C       -0.28697787052075       1.44921856031341        0.00000000000000
C       -1.34533244262240       0.47742818824955        0.00000000000000
C       -1.02403775273833       -0.93718165646021       0.00000000000000
C       0.28697787052075        -1.44921856031341       0.00000000000000
H       2.26911771741755        2.66318896935767        0.00000000000000
```

```
H       -2.26911771741755       -2.66318896935767       0.00000000000000
C       -2.76059560022911        0.57914234080019       0.00000000000000
C       -3.28840682258722       -0.76634637926508       0.00000000000000
N       -2.20685750706933       -1.65009439435554       0.00000000000000
N       -7.90115662691579       -2.58434368065994       0.00000000000000
C       -3.68249731260218        1.66642875536126       0.00000000000000
C       -5.03384314895605        1.41108462943945       0.00000000000000
C       -5.55328806921529        0.06123883803971       0.00000000000000
C       -4.63742615302846       -1.04238953597673       0.00000000000000
N        7.90115662691579        2.58434368065994       0.00000000000000
H       -5.74040074770254        2.23796313739927       0.00000000000000
H       -5.01245548684668       -2.06403167892317       0.00000000000000
C        6.95783349173640        0.17017583318186       0.00000000000000
C        7.89491559457924       -0.89889848547871       0.00000000000000
C        7.49042447299756        1.48868756300815       0.00000000000000
N        8.65821982400693       -1.78569915882459       0.00000000000000
C       -6.95783349173640       -0.17017583318186       0.00000000000000
C       -7.89491559457924        0.89889848547871       0.00000000000000
C       -7.49042447299756       -1.48868756300815       0.00000000000000
C        0.50768121306312       -2.84134152566155       0.00000000000000
C       -0.50768121306312        2.84134152566155       0.00000000000000
C        0.63628306568872       -4.05979441348447       0.00000000000000
C       -0.63628306568872        4.05979441348447       0.00000000000000
N       -0.94146995931026        6.57792979665202       0.00000000000000
N        0.94146995931026       -6.57792979665202       0.00000000000000
C       -0.80218658346764        5.41252176002381       0.00000000000000
C        0.80218658346764       -5.41252176002381       0.00000000000000
```

The triplet-optimized C_{2h} geometry of molecule **PCN1** is given below (BLYP/TZP level of theory).
Total bonding energy: -12.316558504943446 $a.u.$

```
46
C30H8N8 optimized with ADF in AMS.
C        3.29550431844435        0.75490353777245       0.00000000000000
C        2.77888762294509       -0.58419428324155       0.00000000000000
N        2.21742060967508        1.63960696415882       0.00000000000000
C        4.65560829725242        1.03251982758330       0.00000000000000
C        3.69281688654530       -1.66473706604738       0.00000000000000
C        5.05310254692978       -1.40636695489112       0.00000000000000
C        5.56135063478018       -0.06409372620165       0.00000000000000
N       -8.68222510149013        1.78229184671493       0.00000000000000
H        5.02715895466157        2.05546778654604       0.00000000000000
H       -3.33664154232286        2.69154046606597       0.00000000000000
C        1.34090872642173       -0.47778820039980       0.00000000000000
H        3.33664154232286       -2.69154046606597       0.00000000000000
H        5.75819653167770       -2.23439070422751       0.00000000000000
C        1.03135682295952        0.92519871969612       0.00000000000000
C       -0.28824661012367        1.44095646437551       0.00000000000000
C       -1.34090872642173        0.47778820039980       0.00000000000000
C       -1.03135682295952       -0.92519871969612       0.00000000000000
C        0.28824661012367       -1.44095646437551       0.00000000000000
H        2.28161700919907        2.65219709855353       0.00000000000000
H       -2.28161700919907       -2.65219709855353       0.00000000000000
C       -2.77888762294509        0.58419428324155       0.00000000000000
C       -3.29550431844435       -0.75490353777245       0.00000000000000
N       -2.21742060967508       -1.63960696415882       0.00000000000000
N       -7.91759937932351       -2.59087858957055       0.00000000000000
C       -3.69281688654530        1.66473706604738       0.00000000000000
```

```
C       -5.05310254692978       1.40636695489112        0.00000000000000
C       -5.56135063478018       0.06409372620165        0.00000000000000
C       -4.65560829725242       -1.03251982758330       0.00000000000000
N       7.91759937932351        2.59087858957055        0.00000000000000
H       -5.75819653167770       2.23439070422751        0.00000000000000
H       -5.02715895466157       -2.05546778654604       0.00000000000000
C       6.98820737058560        0.17475216183233        0.00000000000000
C       7.92192821812864        -0.89128588181832       0.00000000000000
C       7.51380985301193        1.49143618087510        0.00000000000000
N       8.68222510149013        -1.78229184671493       0.00000000000000
C       -6.98820737058560       -0.17475216183233       0.00000000000000
C       -7.92192821812864       0.89128588181832        0.00000000000000
C       -7.51380985301193       -1.49143618087510       0.00000000000000
C       0.50482028304856        -2.83386407109146       0.00000000000000
C       -0.50482028304856       2.83386407109146        0.00000000000000
C       0.62822721247520        -4.05271839547664       0.00000000000000
C       -0.62822721247520       4.05271839547664        0.00000000000000
N       -0.92388872040015       6.57177181339488        0.00000000000000
N       0.92388872040015        -6.57177181339488       0.00000000000000
C       -0.78924632860105       5.40594568397757        0.00000000000000
C       0.78924632860105        -5.40594568397757       0.00000000000000
```

The closed-shell singlet-optimized C_{2h} geometry of molecule **PCN2** is given below (BLYP/TZP level of theory). Total bonding energy: -13.456848496976015 $a.u.$

```
50
C34H8N8 optimized with ADF in AMS.
C       3.28811515625945        0.76659050779359        0.00000000000000
C       2.75966701443937        -0.57885110556198       0.00000000000000
N       2.20666934043334        1.64967135252542        0.00000000000000
C       4.63749823446895        1.04190831830684        0.00000000000000
C       3.68108078352642        -1.66646689423017       0.00000000000000
C       5.03255431488273        -1.41163382423274       0.00000000000000
C       5.55273131877370        -0.06197173740741       0.00000000000000
N       -8.65521835521984       1.78983831450149        0.00000000000000
H       5.01315788567502        2.06331969611051        0.00000000000000
H       -3.31950693001814       2.69143022946258        0.00000000000000
C       1.34470962909428        -0.47695590950622       0.00000000000000
H       3.31950693001814        -2.69143022946258       0.00000000000000
H       5.73906929962150        -2.23856279841540       0.00000000000000
C       1.02372934394172        0.93654739980543        0.00000000000000
C       -0.28746942480778       1.45309143352653        0.00000000000000
C       -1.34470962909428       0.47695590950622        0.00000000000000
C       -1.02372934394172       -0.93654739980543       0.00000000000000
C       0.28746942480778        -1.45309143352653       0.00000000000000
H       2.26726278191683        2.66282470209140        0.00000000000000
H       -2.26726278191683       -2.66282470209140       0.00000000000000
C       -2.75966701443937       0.57885110556198        0.00000000000000
C       -3.28811515625945       -0.76659050779359       0.00000000000000
N       -2.20666934043334       -1.64967135252542       0.00000000000000
N       -7.90112121550626       -2.58252913383397       0.00000000000000
C       -3.68108078352642       1.66646689423017        0.00000000000000
C       -5.03255431488273       1.41163382423274        0.00000000000000
C       -5.55273131877370       0.06197173740741        0.00000000000000
C       -4.63749823446895       -1.04190831830684       0.00000000000000
N       7.90112121550626        2.58252913383397        0.00000000000000
H       -5.73906929962150       2.23856279841540        0.00000000000000
H       -5.01315788567502       -2.06331969611051       0.00000000000000
```

C	6.95765616634413	0.16845955015950	0.00000000000000
C	7.89362996952682	-0.90150671780594	0.00000000000000
C	7.49078302044124	1.48668163017611	0.00000000000000
N	8.65521835521984	-1.78983831450149	0.00000000000000
C	-6.95765616634413	-0.16845955015950	0.00000000000000
C	-7.89362996952682	0.90150671780594	0.00000000000000
C	-7.49078302044124	-1.48668163017611	0.00000000000000
C	0.50850969604009	-2.84105657376930	0.00000000000000
C	-0.50850969604009	2.84105657376930	0.00000000000000
C	0.64001246522565	-4.06610161319271	0.00000000000000
C	-0.64001246522565	4.06610161319271	0.00000000000000
N	-1.26150402677037	9.13698460017957	0.00000000000000
N	1.26150402677037	-9.13698460017957	0.00000000000000
C	-0.80407830045482	5.40091178694309	0.00000000000000
C	-0.95366598522306	6.62302931786158	0.00000000000000
C	0.80407830045482	-5.40091178694309	0.00000000000000
C	0.95366598522306	-6.62302931786158	0.00000000000000
C	-1.11893385434077	7.97016582272874	0.00000000000000
C	1.11893385434077	-7.97016582272874	0.00000000000000

The triplet-optimized C_{2h} geometry of molecule **PCN2** is given below (BLYP/TZP level of theory).
Total bonding energy: -13.451035074091484 *a.u.*

50
C34H8N8 optimized with ADF in AMS.

C	3.29481033855515	0.75746864011987	0.00000000000000
C	2.77832700763079	-0.58203514071449	0.00000000000000
N	2.21593208815857	1.64064004555038	0.00000000000000
C	4.65473074607331	1.03559583551432	0.00000000000000
C	3.69301711223929	-1.66218927106306	0.00000000000000
C	5.05301894154408	-1.40330088739569	0.00000000000000
C	5.56115092848263	-0.06062547216788	0.00000000000000
N	-8.68134739412472	1.77882128550013	0.00000000000000
H	5.02609183300865	2.05862390636264	0.00000000000000
H	-3.33599707885797	2.68874547139497	0.00000000000000
C	1.34110154602387	-0.47658801411420	0.00000000000000
H	3.33599707885797	-2.68874547139497	0.00000000000000
H	5.75888023979990	-2.23069665291746	0.00000000000000
C	1.03064009964920	0.92508929732849	0.00000000000000
C	-0.28979724172533	1.44482449306487	0.00000000000000
C	-1.34110154602387	0.47658801411420	0.00000000000000
C	-1.03064009964920	-0.92508929732849	0.00000000000000
C	0.28979724172533	-1.44482449306487	0.00000000000000
H	2.27761403167091	2.65335828070456	0.00000000000000
H	-2.27761403167091	-2.65335828070456	0.00000000000000
C	-2.77832700763079	0.58203514071449	0.00000000000000
C	-3.29481033855515	-0.75746864011987	0.00000000000000
N	-2.21593208815857	-1.64064004555038	0.00000000000000
N	-7.91562472583237	-2.59520105248068	0.00000000000000
C	-3.69301711223929	1.66218927106306	0.00000000000000
C	-5.05301894154408	1.40330088739569	0.00000000000000
C	-5.56115092848263	0.06062547216788	0.00000000000000
C	-4.65473074607331	-1.03559583551432	0.00000000000000
N	7.91562472583237	2.59520105248068	0.00000000000000
H	-5.75888023979990	2.23069665291746	0.00000000000000
H	-5.02609183300865	-2.05862390636264	0.00000000000000
C	6.98749199064805	0.17849065422953	0.00000000000000
C	7.92148878002551	-0.88745256819008	0.00000000000000

```
C       7.51275164873755        1.49543111518713        0.00000000000000
N       8.68134739412472        -1.77882128550013       0.00000000000000
C       -6.98749199064805       -0.17849065422953       0.00000000000000
C       -7.92148878002551       0.88745256819008        0.00000000000000
C       -7.51275164873755       -1.49543111518713       0.00000000000000
C       0.50678017238866        -2.83301286917338       0.00000000000000
C       -0.50678017238866       2.83301286917338        0.00000000000000
C       0.63207815749925        -4.05852868810963       0.00000000000000
C       -0.63207815749925       4.05852868810963        0.00000000000000
N       -1.24067573646139       9.12935928513138        0.00000000000000
N       1.24067573646139        -9.12935928513138       0.00000000000000
C       -0.79612332594160       5.39261219634754        0.00000000000000
C       -0.94011317815142       6.61525212647595        0.00000000000000
C       0.79612332594160        -5.39261219634754       0.00000000000000
C       0.94011317815142        -6.61525212647595       0.00000000000000
C       -1.10186790850307       7.96224283515076        0.00000000000000
C       1.10186790850307        -7.96224283515076       0.00000000000000
```

The closed-shell singlet-optimized C_{2h} geometry of molecule **PN** is given below (BLYP/TZP level of theory). Total bonding energy: -11.147000532214243 *a.u.*

```
44
C24H12N8 optimized with ADF in AMS.
C       -3.30500071009967        0.73044450784766       0.00000000000000
C       -2.74950739733851       -0.60710829215299       0.00000000000000
N       -2.24760368549589        1.62706787119516       0.00000000000000
C       -4.66518627035174        0.98684663971017       0.00000000000000
C       -3.66969174624781       -1.69664082905621       0.00000000000000
C       -5.02525459826592       -1.45610361778450       0.00000000000000
C       -5.56615331730052       -0.11550704205854       0.00000000000000
N        8.62233517240769        1.92499738107396       0.00000000000000
H       -5.04902289721232        2.00537260653674       0.00000000000000
H        3.33520065756573        2.73209684582341       0.00000000000000
C       -1.33010294474781       -0.47828019351747       0.00000000000000
H       -3.33520065756573       -2.73209684582341       0.00000000000000
H       -5.71944349309150       -2.29354192890631       0.00000000000000
C       -1.02819944127914        0.92832210884183       0.00000000000000
C        0.26435681727488        1.45126418039393       0.00000000000000
C        1.33010294474781        0.47828019351747       0.00000000000000
C        1.02819944127914       -0.92832210884183       0.00000000000000
C       -0.26435681727488       -1.45126418039393       0.00000000000000
H       -2.36511224007746        2.63211010180093       0.00000000000000
H        2.36511224007746       -2.63211010180093       0.00000000000000
C        2.74950739733851        0.60710829215299       0.00000000000000
C        3.30500071009967       -0.73044450784766       0.00000000000000
N        2.24760368549589       -1.62706787119516       0.00000000000000
N        7.95211924517752       -2.48380577510032       0.00000000000000
C        3.66969174624781        1.69664082905621       0.00000000000000
C        5.02525459826592        1.45610361778450       0.00000000000000
C        5.56615331730052        0.11550704205854       0.00000000000000
C        4.66518627035174       -0.98684663971017       0.00000000000000
N       -7.95211924517752        2.48380577510032       0.00000000000000
H        5.71944349309150        2.29354192890631       0.00000000000000
H        5.04902289721232       -2.00537260653674       0.00000000000000
C       -6.98400528433242        0.08144371437757       0.00000000000000
C       -7.89061665427289       -1.01039213100202       0.00000000000000
C       -7.53665342719775        1.38868314154988       0.00000000000000
N       -8.62233517240769       -1.92499738107396       0.00000000000000
```

C	6.98400528433242	-0.08144371437757	0.00000000000000
C	7.89061665427289	1.01039213100202	0.00000000000000
C	7.53665342719775	-1.38868314154988	0.00000000000000
N	-0.52117945363477	-2.80770067836561	0.00000000000000
N	0.52117945363477	2.80770067836561	0.00000000000000
H	1.46560893071089	3.16078451842805	0.00000000000000
H	-0.21960885764905	3.49371267415206	0.00000000000000
H	-1.46560893071089	-3.16078451842805	0.00000000000000
H	0.21960885764905	-3.49371267415206	0.00000000000000

The triplet-optimized C_{2h} geometry of molecule **PN** is given below (BLYP/TZP level of theory).
Total bonding energy: -11.12757310000000 [a] $a.u.$

44
C24H12N8 optimized with ADF in AMS.

C	-3.32020336000000	0.68252912000000	0.00000000000000
C	-2.77240313000000	-0.64326148000000	0.00000000000000
N	-2.25120874000000	1.58430743000000	0.00000000000000
C	-4.67965641000000	0.96079167000000	0.00000000000000
C	-3.69556972000000	-1.71955536000000	0.00000000000000
C	-5.05741279000000	-1.46628727000000	0.00000000000000
C	-5.59168449000000	-0.13110195000000	0.00000000000000
N	8.66152940000000	1.90402383000000	0.00000000000000
H	-5.04950215000000	1.98474173000000	0.00000000000000
H	3.37122183000000	2.75948346000000	0.00000000000000
C	-1.33786815000000	-0.50343462000000	0.00000000000000
H	-3.37122183000000	-2.75948346000000	0.00000000000000
H	-5.75438510000000	-2.30158891000000	0.00000000000000
C	-1.04733890000000	0.89785174000000	0.00000000000000
C	0.26745687000000	1.44330779000000	0.00000000000000
C	1.33786815000000	0.50343462000000	0.00000000000000
C	1.04733890000000	-0.89785174000000	0.00000000000000
C	-0.26745687000000	-1.44330779000000	0.00000000000000
H	-2.37312153000000	2.59057307000000	0.00000000000000
H	2.37312153000000	-2.59057307000000	0.00000000000000
C	2.77240313000000	0.64326148000000	0.00000000000000
C	3.32020336000000	-0.68252912000000	0.00000000000000
N	2.25120874000000	-1.58430743000000	0.00000000000000
N	7.91047083000000	-2.52260628000000	0.00000000000000
C	3.69556972000000	1.71955536000000	0.00000000000000
C	5.05741279000000	1.46628727000000	0.00000000000000
C	5.59168449000000	0.13110195000000	0.00000000000000
C	4.67965641000000	-0.96079167000000	0.00000000000000
N	-7.91047083000000	2.52260628000000	0.00000000000000
H	5.75438510000000	2.30158891000000	0.00000000000000
H	5.04950215000000	-1.98474173000000	0.00000000000000
C	-7.01651123000000	0.09423061000000	0.00000000000000
C	-7.93170291000000	-0.98616068000000	0.00000000000000
C	-7.53631854000000	1.41154345000000	0.00000000000000
N	-8.66152940000000	-1.90402383000000	0.00000000000000
C	7.01651123000000	-0.09423061000000	0.00000000000000
C	7.93170291000000	0.98616068000000	0.00000000000000
C	7.53631854000000	-1.41154345000000	0.00000000000000
N	-0.49429431000000	-2.79573416000000	0.00000000000000
N	0.49429431000000	2.79573416000000	0.00000000000000
H	1.43719199000000	3.15979041000000	0.00000000000000
H	-0.25457633000000	3.47421386000000	0.00000000000000
H	-1.43719199000000	-3.15979041000000	0.00000000000000

H 0.25457633000000 -3.47421386000000 0.00000000000000

This geometry was not completely optimized and that is why the energy is higher. Nonetheless, this geometry is very close to optimized, and this was demonstrated in *ab-initio* calculations.

The closed-shell singlet-optimized C_{2h} geometry of molecule **PN1** is given below (BLYP/TZP level of theory). Total bonding energy: -12.266740770523901 *a.u.*

```
48
C28H12N8 optimized with ADF in AMS.
C       3.26497928821315      0.85685777466606      0.00000000000000
C       2.76485692517484     -0.50223118345408      0.00000000000000
N       2.16933056077114      1.70902520226471      0.00000000000000
C       4.61396130098373      1.15707172530686      0.00000000000000
C       3.71116497869496     -1.56641852939118      0.00000000000000
C       5.05746047802814     -1.28207151352370      0.00000000000000
C       5.55039905232399      0.07856447887768      0.00000000000000
N      -8.67751056222009      1.60839784646888      0.00000000000000
H       4.96787897342973      2.18631100474602      0.00000000000000
H      -3.36894903094431      2.59783508443738      0.00000000000000
C       1.34640948470599     -0.43584582050824      0.00000000000000
H       3.36894903094431     -2.59783508443738      0.00000000000000
H       5.78253462835938     -2.09299008838776      0.00000000000000
C       0.99023296091974      0.96364260582726      0.00000000000000
C      -0.32168862113300      1.45255665219024      0.00000000000000
C      -1.34640948470599      0.43584582050824      0.00000000000000
C      -0.99023296091974     -0.96364260582726      0.00000000000000
C       0.32168862113300     -1.45255665219024      0.00000000000000
H      -2.20540709603010     -2.72168904331192      0.00000000000000
H       2.20540709603010      2.72168904331192      0.00000000000000
C      -2.76485692517484      0.50223118345408      0.00000000000000
C      -3.26497928821315     -0.85685777466606      0.00000000000000
N      -2.16933056077114     -1.70902520226471      0.00000000000000
N      -7.85312092373613     -2.76092037451948      0.00000000000000
C      -3.71116497869496      1.56641852939118      0.00000000000000
C      -5.05746047802814      1.28207151352370      0.00000000000000
C      -5.55039905232399     -0.07856447887768      0.00000000000000
C      -4.61396130098373     -1.15707172530686      0.00000000000000
N       7.85312092373613      2.76092037451948      0.00000000000000
H      -5.78253462835938      2.09299008838776      0.00000000000000
H      -4.96787897342973     -2.18631100474602      0.00000000000000
C       6.95716154678651      0.32951028095171      0.00000000000000
C       7.90681826702896     -0.72667808490387      0.00000000000000
C       7.46674488658126      1.65542994969479      0.00000000000000
N       8.67751056222009     -1.60839784646888      0.00000000000000
C      -6.95716154678651     -0.32951028095171      0.00000000000000
C      -7.90681826702896      0.72667808490387      0.00000000000000
C      -7.46674488658126     -1.65542994969479      0.00000000000000
H      -1.94994193157497      5.72632164269293      0.00000000000000
C      -0.59188222770536      2.83929337757412      0.00000000000000
C      -0.76962226958331      4.04911873750820      0.00000000000000
H      -0.24410688239673      6.03326044795684      0.00000000000000
N      -1.00363777193831      5.36249507678551      0.00000000000000
C       0.59188222770536     -2.83929337757412      0.00000000000000
C       0.76962226958331     -4.04911873750820      0.00000000000000
N       1.00363777193831     -5.36249507678551      0.00000000000000
H       1.94994193157497     -5.72632164269293      0.00000000000000
H       0.24410688239673     -6.03326044795684      0.00000000000000
```

The triplet-optimized C_{2h} geometry of molecule **PN1** is given below (BLYP/TZP level of theory).
Total bonding energy: -12.250933153430955 *a.u.*

```
48
C28H12N8 optimized with ADF in AMS.
C       3.28609909863791       0.81333175224362        0.00000000000000
C       2.79366275062631       -0.53523423001235       0.00000000000000
N       2.17734384341790       1.66497262132512        0.00000000000000
C       4.63356134242727       1.14109765263753        0.00000000000000
C       3.74855641384927       -1.57977029863160       0.00000000000000
C       5.09998576890959       -1.27528930976030       0.00000000000000
C       5.58474456844184       0.08029230499170        0.00000000000000
N       -8.72218278229719      1.57468678383764        0.00000000000000
H       4.96733851517760       2.17724221751028        0.00000000000000
H       -3.42824352993185      2.61872909077749        0.00000000000000
C       1.35848369739306       -0.46087409614460       0.00000000000000
H       3.42824352993185       -2.61872909077749       0.00000000000000
H       5.83012803882530       -2.08193167699550       0.00000000000000
C       1.01135616280256       0.93016912296591        0.00000000000000
C       -0.32147945489507      1.44433944509335        0.00000000000000
C       -1.35848369739306      0.46087409614460        0.00000000000000
C       -1.01135616280256      -0.93016912296591       0.00000000000000
C       0.32147945489507       -1.44433944509335       0.00000000000000
H       -2.21533111375998      -2.67939617916050       0.00000000000000
H       2.21533111375998       2.67939617916050        0.00000000000000
C       -2.79366275062631      0.53523423001235        0.00000000000000
C       -3.28609909863791      -0.81333175224362       0.00000000000000
N       -2.17734384341790      -1.66497262132512       0.00000000000000
N       -7.81713903442597      -2.81318281855756       0.00000000000000
C       -3.74855641384927      1.57977029863160        0.00000000000000
C       -5.09998576890959      1.27528930976030        0.00000000000000
C       -5.58474456844184      -0.08029230499170       0.00000000000000
C       -4.63356134242727      -1.14109765263753       0.00000000000000
N       7.81713903442597       2.81318281855756        0.00000000000000
H       -5.83012803882530      2.08193167699550        0.00000000000000
H       -4.96733851517760      -2.17724221751028       0.00000000000000
C       6.99879420925785       0.35718196454361        0.00000000000000
C       7.95490528264857       -0.68807630126032       0.00000000000000
C       7.47425908594018       1.69180308679947        0.00000000000000
N       8.72218278229719       -1.57468678383764       0.00000000000000
C       -6.99879420925785      -0.35718196454361       0.00000000000000
C       -7.95490528264857      0.68807630126032        0.00000000000000
C       -7.47425908594018      -1.69180308679947       0.00000000000000
H       -1.88875323409245      5.72081584729101        0.00000000000000
C       -0.55789795689173      2.82715149550046        0.00000000000000
C       -0.72078703582852      4.04243309915695        0.00000000000000
H       -0.17934051666929      6.01749810363668        0.00000000000000
N       -0.94358453410712      5.35025681351067        0.00000000000000
C       0.55789795689173       -2.82715149550046       0.00000000000000
C       0.72078703582852       -4.04243309915695       0.00000000000000
N       0.94358453410712       -5.35025681351067       0.00000000000000
H       1.88875323409245       -5.72081584729101       0.00000000000000
H       0.17934051666929       -6.01749810363668       0.00000000000000
```

The closed-shell singlet-optimized C_{2h} geometry of molecule **PN2** is given below (BLYP/TZP level of theory).
Total bonding energy: -13.405472967436216 *a.u.*

```
52
```

```
C32H12N8 optimized with ADF in AMS.
C        3.19511119279560      -1.09041564094158       0.00000000000000
C        2.79655056819289       0.30146121268837       0.00000000000000
N        2.03851901565166      -1.86154711069116       0.00000000000000
C        4.51738179472242      -1.48876454371746       0.00000000000000
C        3.81638345858741       1.29613650001001       0.00000000000000
C        5.13801751171659       0.91343992152723       0.00000000000000
C        5.53000164688754      -0.47953653614667       0.00000000000000
N       -8.77738043700892      -0.96625686472163       0.00000000000000
H        4.79552877928776      -2.54100074454633       0.00000000000000
H       -3.55036614868317      -2.35002677099489       0.00000000000000
C        1.37783901334410       0.33741355749100       0.00000000000000
H        3.55036614868317       2.35002677099489       0.00000000000000
H        5.92017412768546       1.66944477242251       0.00000000000000
C        0.92148954481291      -1.03289915972184       0.00000000000000
C       -0.42704547044204      -1.42434946780623       0.00000000000000
C       -1.37783901334410      -0.33741355749100       0.00000000000000
C       -0.92148954481291       1.03289915972184       0.00000000000000
C        0.42704547044204       1.42434946780623       0.00000000000000
H        1.99768851377025      -2.87487899991879       0.00000000000000
H       -1.99768851377025       2.87487899991879       0.00000000000000
C       -2.79655056819289      -0.30146121268837       0.00000000000000
C       -3.19511119279560       1.09041564094158       0.00000000000000
N       -2.03851901565166       1.86154711069116       0.00000000000000
N       -7.63155305580096       3.32422744615678       0.00000000000000
C       -3.81638345858741      -1.29613650001001       0.00000000000000
C       -5.13801751171659      -0.91343992152723       0.00000000000000
C       -5.53000164688754       0.47953653614667       0.00000000000000
C       -4.51738179472242       1.48876454371746       0.00000000000000
N        7.63155305580096      -3.32422744615678       0.00000000000000
H       -5.92017412768546      -1.66944477242251       0.00000000000000
H       -4.79552877928776       2.54100074454633       0.00000000000000
C        6.91340901240879      -0.83423747969129       0.00000000000000
C        7.93981203547995       0.14796081845944       0.00000000000000
C        7.32494704764784      -2.19413665979516       0.00000000000000
N        8.77738043700892       0.96625686472163       0.00000000000000
C       -6.91340901240879       0.83423747969129       0.00000000000000
C       -7.93981203547995      -0.14796081845944       0.00000000000000
C       -7.32494704764784       2.19413665979516       0.00000000000000
C        0.79142512651316       2.78222601089006       0.00000000000000
C       -0.79142512651316      -2.78222601089006       0.00000000000000
C        1.04817600521331       3.98732555796053       0.00000000000000
C       -1.04817600521331      -3.98732555796053       0.00000000000000
N        1.94708256692413       7.78831817060308       0.00000000000000
H       -2.91320328146792      -8.09898156241258       0.00000000000000
C       -1.35869195024179      -5.30074426416969       0.00000000000000
C       -1.63959994231406      -6.49662433003038       0.00000000000000
C        1.35869195024179       5.30074426416969       0.00000000000000
C        1.63959994231406       6.49662433003038       0.00000000000000
N       -1.94708256692413      -7.78831817060308       0.00000000000000
H       -1.22509926805776      -8.50137716654254       0.00000000000000
H        2.91320328146792       8.09898156241258       0.00000000000000
H        1.22509926805776       8.50137716654254       0.00000000000000
```

The triplet-optimized C_{2h} geometry of molecule **PN2** is given below (BLYP/TZP level of theory).
Total bonding energy: -13.387146455656104 *a.u.*

```
52
```

```
C32H12N8 optimized with ADF in AMS.
C        3.19940605148809      -1.08564949411433       0.00000000000000
C        2.81370339764382       0.30094229761961       0.00000000000000
N        2.04218605333945      -1.85540314318390       0.00000000000000
C        4.52753541027211      -1.49077459259683       0.00000000000000
C        3.82950540397672       1.28687368646467       0.00000000000000
C        5.15832152740909       0.89819966547932       0.00000000000000
C        5.53655763851461      -0.48796588744551       0.00000000000000
N       -8.80732503843008      -0.92685227910810       0.00000000000000
H        4.79941268113512      -2.54469279414694       0.00000000000000
H       -3.56936494320008      -2.34231930440982       0.00000000000000
C        1.37511035570786       0.33944664015552       0.00000000000000
H        3.56936494320008       2.34231930440982       0.00000000000000
H        5.94131178894350       1.65318508280328       0.00000000000000
C        0.92686176360528      -1.02277820735882       0.00000000000000
C       -0.43134802433916      -1.41769065373187       0.00000000000000
C       -1.37511035570786      -0.33944664015552       0.00000000000000
C       -0.92686176360528       1.02277820735882       0.00000000000000
C        0.43134802433916       1.41769065373187       0.00000000000000
H        1.99859795363676      -2.86844523890847       0.00000000000000
H       -1.99859795363676       2.86844523890847       0.00000000000000
C       -2.81370339764382      -0.30094229761961       0.00000000000000
C       -3.19940605148809       1.08564949411433       0.00000000000000
N       -2.04218605333945       1.85540314318390       0.00000000000000
N       -7.63140072808525       3.35337318979715       0.00000000000000
C       -3.82950540397672      -1.28687368646467       0.00000000000000
C       -5.15832152740909      -0.89819966547932       0.00000000000000
C       -5.53655763851461       0.48796588744551       0.00000000000000
C       -4.52753541027211       1.49077459259683       0.00000000000000
N        7.63140072808525      -3.35337318979715       0.00000000000000
H       -5.94131178894350      -1.65318508280328       0.00000000000000
H       -4.79941268113512       2.54469279414694       0.00000000000000
C        6.93349346873800      -0.85988609965084       0.00000000000000
C        7.96458609957393       0.11278749710585       0.00000000000000
C        7.33307981970114      -2.22026646913660       0.00000000000000
N        8.80732503843008       0.92685227910810       0.00000000000000
C       -6.93349346873800       0.85988609965084       0.00000000000000
C       -7.96458609957393      -0.11278749710585       0.00000000000000
C       -7.33307981970114       2.22026646913660       0.00000000000000
C        0.78611195883497       2.77866571155418       0.00000000000000
C       -0.78611195883497      -2.77866571155418       0.00000000000000
C        1.03675722783875       3.98521630199187       0.00000000000000
C       -1.03675722783875      -3.98521630199187       0.00000000000000
N        1.90605514145512       7.79389436252228       0.00000000000000
H       -2.87014521875255      -8.11014876889809       0.00000000000000
C       -1.33398634816146      -5.30191261114786       0.00000000000000
C       -1.60620774516994      -6.49977842347576       0.00000000000000
C        1.33398634816146       5.30191261114786       0.00000000000000
C        1.60620774516994       6.49977842347576       0.00000000000000
N       -1.90605514145512      -7.79389436252228       0.00000000000000
H       -1.17976834942673      -8.50229929064032       0.00000000000000
H        2.87014521875255       8.11014876889809       0.00000000000000
H        1.17976834942673       8.50229929064032       0.00000000000000
```

The closed-shell singlet-optimized C_s geometry of molecule **PM** is given below (BLYP/TZP level of theory). Total bonding energy: -11.178275915981327 *a.u.*

```
43
```

```
C25H10N8 optimized with ADF in AMS.
C        3.31809856493817      -0.69343416809997       0.00000000000000
C        2.76619632701401       0.64129728780726       0.00000000000000
N        2.25870206875754      -1.59548309329937       0.00000000000000
C        4.67767947328666      -0.93829523715701       0.00000000000000
C        3.66164502449318       1.74924695775942       0.00000000000000
C        5.01848149548516       1.52062943529111       0.00000000000000
C        5.56599601898706       0.18143779920002       0.00000000000000
N       -8.63220983353473      -1.77778056047991       0.00000000000000
H        5.07599216229209      -1.95112875343995       0.00000000000000
H       -3.34587552203829      -2.67579326131081       0.00000000000000
C        1.35057950951373       0.51500022783248       0.00000000000000
H        3.27863500652569       2.76705644642209       0.00000000000000
H        5.70792322712702       2.36184179884968       0.00000000000000
C        1.04590328673233      -0.89088968848654       0.00000000000000
C       -0.25637882816839      -1.41097900806046       0.00000000000000
C       -1.31594672264850      -0.44835376448776       0.00000000000000
C       -1.00660451824332       0.97118382506972       0.00000000000000
C        0.29190820877874       1.48508022141207       0.00000000000000
H        2.37406180221105      -2.60076717724977       0.00000000000000
H       -2.26743386344710       2.68578659456026       0.00000000000000
C       -2.73750151320754      -0.55981334391356       0.00000000000000
C       -3.27251810882381       0.78485938463229       0.00000000000000
N       -2.19816224404141       1.67275086899258       0.00000000000000
N       -7.89702327901667       2.60588857697830       0.00000000000000
C       -3.67040856734162      -1.63716961000169       0.00000000000000
C       -5.02409565062902      -1.38178994385285       0.00000000000000
C       -5.54278335826345      -0.03501446834084       0.00000000000000
C       -4.62295244254435       1.06153153595300       0.00000000000000
N        7.96443481145857      -2.41361277306589       0.00000000000000
H       -5.72900099010752      -2.20985935086957       0.00000000000000
H       -4.99277930839213       2.08507317481371       0.00000000000000
C        6.98026723824451      -0.01664736465170       0.00000000000000
C        7.89243185120695       1.07194044798587       0.00000000000000
C        7.53843872336442      -1.32320671999344       0.00000000000000
N        8.63694407138183       1.97524087165126       0.00000000000000
C       -6.95098615366919       0.19383138603290       0.00000000000000
C       -7.88110819919766      -0.88002814678718       0.00000000000000
C       -7.48518009229695       1.51023541495927       0.00000000000000
C        0.51103265088499       2.88901495671426       0.00000000000000
N       -0.50777121154564      -2.76667708360795       0.00000000000000
N        0.63769088543454       4.05166042798625       0.00000000000000
H        0.23477616999121      -3.45066184128450       0.00000000000000
H       -1.45109815095216      -3.12320230246309       0.00000000000000
```

The triplet-optimized C_s geometry of molecule **PM** is given below (BLYP/TZP level of theory).
Total bonding energy: -11.174552498699549 *a.u.*

```
43
C25H10N8 optimized with ADF in AMS.
C        3.32279101997152      -0.67436418420569       0.00000000000000
C        2.78214232461670       0.65351253946299       0.00000000000000
N        2.26405062668440      -1.57924833195003       0.00000000000000
C        4.68936939660707      -0.92612231474977       0.00000000000000
C        3.67577598390829       1.75272298727029       0.00000000000000
C        5.04000288926552       1.52026189456277       0.00000000000000
C        5.57440663553769       0.18720546899242       0.00000000000000
N       -8.65251178211609      -1.78451338421646       0.00000000000000
```

```
H        5.08175704617155      -1.94145270010842       0.00000000000000
H       -3.35359672197144      -2.67506433064760       0.00000000000000
C        1.34745677851744       0.52621249393903       0.00000000000000
H        3.29721767562075       2.77231933011384       0.00000000000000
H        5.72971318031061       2.36117304060728       0.00000000000000
C        1.05518556568899      -0.87261425852861       0.00000000000000
C       -0.26021648641347      -1.39921585426414       0.00000000000000
C       -1.31394816447677      -0.44491397512424       0.00000000000000
C       -1.01362524504459       0.96322437145947       0.00000000000000
C        0.29009636631305       1.48516652307804       0.00000000000000
H        2.37985509667954      -2.58454589242088       0.00000000000000
H       -2.27951142909405       2.67709317562223       0.00000000000000
C       -2.75502460042848      -0.56293787612479       0.00000000000000
C       -3.28211911436163       0.77662512271930       0.00000000000000
N       -2.20942931640301       1.66430817121545       0.00000000000000
N       -7.92097912201183       2.60169801847057       0.00000000000000
C       -3.67785962458968      -1.63601708846468       0.00000000000000
C       -5.04060081680482      -1.37964851686928       0.00000000000000
C       -5.55161316478356      -0.04048388760650       0.00000000000000
C       -4.64135225287181       1.05205283288504       0.00000000000000
N        7.96416026974632      -2.43467523589725       0.00000000000000
H       -5.74319059731239      -2.20975780853414       0.00000000000000
H       -5.01033710470438       2.07599697206216       0.00000000000000
C        7.00438741226462      -0.03000371264076       0.00000000000000
C        7.92339461206429       1.04866035649524       0.00000000000000
C        7.54833023757375      -1.33958221696728       0.00000000000000
N        8.67339921398153       1.94846945584920       0.00000000000000
C       -6.97920810697586       0.19064744134823       0.00000000000000
C       -7.90468663088826      -0.88272027617368       0.00000000000000
C       -7.51122660831613       1.50418324813864       0.00000000000000
C        0.50395354182590       2.88996092804241       0.00000000000000
N       -0.49484137263338      -2.75461814418945       0.00000000000000
N        0.62941918899476       4.05290761918662       0.00000000000000
H        0.25451362193321      -3.43139808401235       0.00000000000000
H       -1.43550040207592      -3.12050393782523       0.00000000000000
```

The closed-shell singlet-optimized C_s geometry of molecule **PM1** is given below (BLYP/TZP level of theory). Total bonding energy: -12.296849010722768 *a.u.*

```
47
C29H10N8 optimized with ADF in AMS.
C       3.26659070019729       0.87969508015964        0.00000000000000
C       2.73369174303411       -0.46335073649777       0.00000000000000
N       2.18763798176074       1.76530481036506        0.00000000000000
C       4.61789399051280       1.15028335261630        0.00000000000000
C       3.65188130248843       -1.55113723527859       0.00000000000000
C       5.00551451354819       -1.30172258433446       0.00000000000000
C       5.53035949740998       0.04484120010007        0.00000000000000
N       -8.68261330269868      1.89332356305070        0.00000000000000
H       4.99652511685442       2.17065694103416        0.00000000000000
H       -3.34129030245156      2.80191268402742        0.00000000000000
C       1.31698835329777       -0.36022264666314       0.00000000000000
H       3.28338052688947       -2.57332567544036       0.00000000000000
H       5.70977847933638       -2.13057348847169       0.00000000000000
C       0.99945354141287       1.05684419281476        0.00000000000000
C       -0.30698249293954      1.57320123625303        0.00000000000000
C       -1.36161768585499      0.58993810417327        0.00000000000000
C       -1.03771543124146      -0.81628728698545       0.00000000000000
```

```
C       0.26704528319919        -1.33891170809446       0.00000000000000
H       2.25225635168257        2.77809405971254        0.00000000000000
H       -2.28468114643570       -2.54795399286208       0.00000000000000
C       -2.77819739991329       0.69000884827407        0.00000000000000
C       -3.30528965499665       -0.65774931656427       0.00000000000000
N       -2.22731484705541       -1.53605071068466       0.00000000000000
N       -7.91305234866239       -2.47815915158281       0.00000000000000
C       -3.70093205343982       1.77618783239650        0.00000000000000
C       -5.05258393322578       1.51990517006527        0.00000000000000
C       -5.57203942590180       0.16947920874940        0.00000000000000
C       -4.65812697950027       -0.93090080479908       0.00000000000000
N       7.90079984351101        2.67386843908111        0.00000000000000
H       -5.75958726485884       2.34650223510079        0.00000000000000
H       -5.03373191145192       -1.95234590735470       0.00000000000000
C       6.93851192106893        0.26762848817382        0.00000000000000
C       7.86465013663459        -0.81044123744919       0.00000000000000
C       7.48078618080095        1.58131945793037        0.00000000000000
N       8.61333722250456        -1.71019545818088       0.00000000000000
C       -6.98018566926709       -0.06021993939157       0.00000000000000
C       -7.91732204448114       1.00759473288471        0.00000000000000
C       -7.50972587492985       -1.37918758590629       0.00000000000000
C       0.49520073371012        -2.73277164103606       0.00000000000000
C       -0.52868539772393       2.96286166320606        0.00000000000000
C       0.63488739888459        -3.94776057976754       0.00000000000000
C       -0.65493930439480       4.18257214953244        0.00000000000000
N       -0.95488749788049       6.70147112989429        0.00000000000000
H       0.04591961442982        -5.91266744056657       0.00000000000000
C       -0.81817592811201       5.53495900717228        0.00000000000000
N       0.82629784023304        -5.26559976992587       0.00000000000000
H       1.76028966401558        -5.66091880893062       0.00000000000000
```

The triplet-optimized C_s geometry of molecule **PM1** is given below (BLYP/TZP level of theory).
Total bonding energy: -12.292058876577441 *a.u.*

```
47
C29H10N8 optimized with ADF in AMS.
C       3.30961378333444        0.64153120178742        0.00000000000000
C       2.79358190048171        -0.69929001663950       0.00000000000000
N       2.23289279714577        1.52231380980034        0.00000000000000
C       4.67014349332887        0.92025686447071        0.00000000000000
C       3.71274976152928        -1.77741760033636       0.00000000000000
C       5.07208517432740        -1.51764371695049       0.00000000000000
C       5.57915888604889        -0.17401555317044       0.00000000000000
N       -8.63707080000050       1.71120507690878        0.00000000000000
H       5.04047668202384        1.94373556828664        0.00000000000000
H       -3.29938764031349       2.57315411131077        0.00000000000000
C       1.35727454778721        -0.59709107800614       0.00000000000000
H       3.35748677326308        -2.80472112110850       0.00000000000000
H       5.77870701726706        -2.34441292270597       0.00000000000000
C       1.04570829914090        0.80015867835802        0.00000000000000
C       -0.27171851922468       1.32915802698430        0.00000000000000
C       -1.31576115523527       0.35904454975840        0.00000000000000
C       -1.00773010000047       -1.04650275599330       0.00000000000000
C       0.30471556550612        -1.56957024024166       0.00000000000000
H       2.29071469910881        2.53406014200482        0.00000000000000
H       -2.26520479771623       -2.76761500393955       0.00000000000000
C       -2.75352243822255       0.46771586932795        0.00000000000000
C       -3.27704956355729       -0.87036880452066       0.00000000000000
```

```
N       -2.19985134993320       -1.75515345797445       0.00000000000000
N       -7.92373146594000       -2.67526475125532       0.00000000000000
C       -3.66362442311495       1.54932445755048        0.00000000000000
C       -5.02594950354423       1.29695423855537        0.00000000000000
C       -5.54128359822368       -0.04261554649861       0.00000000000000
C       -4.63747049769073       -1.14186658881212       0.00000000000000
N       7.91413280820785        2.49661887956898        0.00000000000000
H       -5.72871953543839       2.12706056585042        0.00000000000000
H       -5.01312513752350       -2.16337377843049       0.00000000000000
C       7.00375793313906        0.07266027468552        0.00000000000000
C       7.94541221430868        -0.98644853902290       0.00000000000000
C       7.52055412945300        1.39330927799441        0.00000000000000
N       8.71442793711713        -1.87009409520593       0.00000000000000
C       -6.96925680721780       -0.26894475629097       0.00000000000000
C       -7.89139681710130       0.80763040991203        0.00000000000000
C       -7.50764905573357       -1.58016268733083       0.00000000000000
C       0.52093056669544        -2.95996419205767       0.00000000000000
C       -0.48509680139350       2.72261123286804        0.00000000000000
C       0.64566494536292        -4.18005183129858       0.00000000000000
C       -0.61246642280553       3.93992654725368        0.00000000000000
H       -1.72685320339501       5.65814265998080        0.00000000000000
N       0.94181051786580        -6.69901979364168       0.00000000000000
N       -0.79499556296651       5.25759887485711        0.00000000000000
H       -0.01059035101711       5.89979479434442        0.00000000000000
C       0.80750507486623        -5.53235728098730       0.00000000000000
```

The closed-shell singlet-optimized C_s geometry of molecule **PM2** is given below (BLYP/TZP level of theory). Total bonding energy: -13.432974983410046 *a.u.*

```
51
C33H10N8 optimized with ADF in AMS.
C       -3.21210535229227      -1.00587090054163       0.00000000000000
C       -2.85214018407761       0.39585566222269       0.00000000000000
N       -2.03473439774162      -1.74723069085455       0.00000000000000
C       -4.52117994040490      -1.44153057939089       0.00000000000000
C       -3.89974694782634       1.36223177517076       0.00000000000000
C       -5.21019299984726       0.94375897143431       0.00000000000000
C       -5.56208713089992      -0.45969989560686       0.00000000000000
N        8.75163668227344      -0.60885893589960       0.00000000000000
H       -4.76967661834341      -2.50108055866169       0.00000000000000
H        3.55838471278960      -2.11569758118214       0.00000000000000
C       -1.43455117584239       0.46794762370965       0.00000000000000
H       -3.66622032133789       2.42384895466049       0.00000000000000
H       -6.01253085129969       1.67820048249537       0.00000000000000
C       -0.94399411769014      -0.88995233643686       0.00000000000000
C        0.41668705315700      -1.25072015998430       0.00000000000000
C        1.34272296651633      -0.15120992515958       0.00000000000000
C        0.85615682635613       1.21558393263578       0.00000000000000
C       -0.50532505248760       1.57223431492481       0.00000000000000
H       -1.96648589096139      -2.75934719032637       0.00000000000000
H        1.89029019375920       3.07580979106358       0.00000000000000
C        2.76021417624771      -0.08419730456448       0.00000000000000
C        3.12728767487727       1.31355178971531       0.00000000000000
N        1.94921277600987       2.06272364479815       0.00000000000000
N        7.50567370838100       3.65212052460868       0.00000000000000
C        3.80129616868747      -1.05632323106261       0.00000000000000
C        5.11440899909585      -0.64502443103041       0.00000000000000
C        5.47392268562308       0.75534987587450       0.00000000000000
```

```
C        4.43600985245189        1.74434659312041        0.00000000000000
N       -7.56765533934776       -3.37046210951294        0.00000000000000
H        5.91304101515403       -1.38340113593606        0.00000000000000
H        4.69006192709430        2.80263239859217        0.00000000000000
C       -6.93179242671586       -0.85799886301340        0.00000000000000
C       -7.99085858446294        0.08920401565621        0.00000000000000
C       -7.29892844071268       -2.23113639415183        0.00000000000000
N       -8.85768819211530        0.87578152542334        0.00000000000000
C        6.84450105313912        1.14616965164444        0.00000000000000
C        7.89551427090867        0.18924915589040        0.00000000000000
C        7.22347869752035        2.51621287260314        0.00000000000000
C       -0.89199091708642        2.92173012798149        0.00000000000000
C        0.80683142243587       -2.60181734821762        0.00000000000000
C       -1.16515643833846        4.12444662903593        0.00000000000000
C        1.08500425033719       -3.80258677733296        0.00000000000000
C       -2.08949003031048        7.94749920015806        0.00000000000000
N       -2.36417602347386        9.09084016352178        0.00000000000000
C        1.41552238672117       -5.10986907982396        0.00000000000000
C        1.71397873432865       -6.30212960212277        0.00000000000000
C       -1.48509551797908        5.42997387405335        0.00000000000000
C       -1.77420548649129        6.62790840279728        0.00000000000000
N        2.03909975765490       -7.58752555516646        0.00000000000000
H        1.32655356332912       -8.31035183892640        0.00000000000000
H        3.00984183323737       -7.88419534888571        0.00000000000000
```

The triplet-optimized C_s geometry of molecule **PM2** is given below (BLYP/TZP level of theory).
Total bonding energy: -13.428031646017946 $a.u.$

```
51
C33H10N8 optimized with ADF in AMS.
C       -3.21603531189293       -0.99442983171758        0.00000000000000
C       -2.86775411145185        0.39969485883518        0.00000000000000
N       -2.03888821782517       -1.73687029343065        0.00000000000000
C       -4.53168184443854       -1.43780726679050        0.00000000000000
C       -3.91189232984937        1.35741658444069        0.00000000000000
C       -5.22900323782331        0.93306896712509        0.00000000000000
C       -5.56802816263651       -0.46273499911206        0.00000000000000
N        8.77733151595748       -0.59650486900052        0.00000000000000
H       -4.77371753305357       -2.49895260160126        0.00000000000000
H        3.57790820005660       -2.10914326210226        0.00000000000000
C       -1.43055376899823        0.47354312879824        0.00000000000000
H       -3.68396826157539        2.42029767505881        0.00000000000000
H       -6.03198329309691        1.66665102439503        0.00000000000000
C       -0.95135906772567       -0.87620997865964        0.00000000000000
C        0.42270328345990       -1.24007260489468        0.00000000000000
C        1.34296883708623       -0.14894862896351        0.00000000000000
C        0.86565201601284        1.20725258636878        0.00000000000000
C       -0.50342237757103        1.56786064263894        0.00000000000000
H       -1.97003943410987       -2.74877843153350        0.00000000000000
H        1.90216452855720        3.06911032667800        0.00000000000000
C        2.78182644828826       -0.08296656415385        0.00000000000000
C        3.13854816189569        1.30881123675684        0.00000000000000
N        1.96134653836184        2.05632258719067        0.00000000000000
N        7.52550746014237        3.66532093258783        0.00000000000000
C        3.81546749169481       -1.04850288702033        0.00000000000000
C        5.13630284971192       -0.63176741260670        0.00000000000000
C        5.48570818213258        0.76100108624089        0.00000000000000
C        4.45576581023603        1.74343307383591        0.00000000000000
```

```
N       -7.56259176585863      -3.39692463561612       0.00000000000000
H        5.93464645000325      -1.37044743971228       0.00000000000000
H        4.70546594129756       2.80277586861398       0.00000000000000
C       -6.95149391791135      -0.88072646874329       0.00000000000000
C       -8.01500129788010       0.05620115273204       0.00000000000000
C       -7.30476062367732      -2.25430528537130       0.00000000000000
N       -8.88632293472311       0.83906458662841       0.00000000000000
C        6.87487888735375       1.15951369064897       0.00000000000000
C        7.92293311312142       0.20495033672459       0.00000000000000
C        7.24872671638991       2.52706065357368       0.00000000000000
C       -0.88611543546272       2.91834724713802       0.00000000000000
C        0.80062122255286      -2.59199130402416       0.00000000000000
C       -1.15788836277864       4.12130622932584       0.00000000000000
C        1.06892602405555      -3.79585369082534       0.00000000000000
C       -2.07707990611724       7.94456205488877       0.00000000000000
N       -2.35085461729307       9.08819043154478       0.00000000000000
C        1.39019004499624      -5.10358658415346       0.00000000000000
C        1.68244315595628      -6.29807735699321       0.00000000000000
C       -1.47586714140343       5.42696128067804       0.00000000000000
C       -1.76277267148231       6.62506304615763       0.00000000000000
N        2.00197755288440      -7.58335590669639       0.00000000000000
H        1.28667274697676      -8.30375903819436       0.00000000000000
H        2.97171745745454      -7.88406976768867       0.00000000000000
```

The closed-shell singlet-optimized C_{2h} geometry of molecule **PD** is given below (BLYP/TZP level of theory). Total bonding energy: -11.530884728134012 *a.u.*

```
46
C26H8F6N6 optimized with ADF in AMS.
C       -3.28647049726794      -0.78567817022835       0.00000000000000
C       -2.77484443278273       0.56439185517989       0.00000000000000
N       -2.20733026728518      -1.66076684916000       0.00000000000000
C       -4.63002488182560      -1.09201537593923       0.00000000000000
C       -3.72911271237586       1.62785824449914       0.00000000000000
C       -5.07440274804885       1.34420265882650       0.00000000000000
C       -5.57026848054122      -0.01290688685067       0.00000000000000
N        8.70923409279822      -1.64905446631531       0.00000000000000
H       -4.97749116130786      -2.12330556634048       0.00000000000000
H        3.41025261271602      -2.66341508855264       0.00000000000000
C       -1.35018684090394       0.47437414459478       0.00000000000000
H       -3.41025261271602       2.66341508855264       0.00000000000000
H       -5.79436211885889       2.15937846956674       0.00000000000000
C       -1.01803997260209      -0.95251226979386       0.00000000000000
C        0.29280463844855      -1.43539187947726       0.00000000000000
C        1.35018684090394      -0.47437414459478       0.00000000000000
C        1.01803997260209       0.95251226979386       0.00000000000000
C       -0.29280463844855       1.43539187947726       0.00000000000000
H       -2.27079502564985      -2.67085448322661       0.00000000000000
H        2.27079502564985       2.67085448322661       0.00000000000000
C        2.77484443278273      -0.56439185517989       0.00000000000000
C        3.28647049726794       0.78567817022835       0.00000000000000
N        2.20733026728518       1.66076684916000       0.00000000000000
N        7.86411798995792       2.70482211004442       0.00000000000000
C        3.72911271237586      -1.62785824449914       0.00000000000000
C        5.07440274804885      -1.34420265882650       0.00000000000000
C        5.57026848054122       0.01290688685067       0.00000000000000
C        4.63002488182560       1.09201537593923       0.00000000000000
N       -7.86411798995792      -2.70482211004442       0.00000000000000
```

```
H        5.79436211885889      -2.15937846956674       0.00000000000000
H        4.97749116130786       2.12330556634048       0.00000000000000
C       -6.96990908784411      -0.27214954702437       0.00000000000000
C       -7.92791369529579       0.77804579671047       0.00000000000000
C       -7.47572525179207      -1.60103424353713       0.00000000000000
N       -8.70923409279822       1.64905446631531       0.00000000000000
C        6.96990908784411       0.27214954702437       0.00000000000000
C        7.92791369529579      -0.77804579671047       0.00000000000000
C        7.47572525179207       1.60103424353713       0.00000000000000
C       -0.61173202483911       2.91170474553767       0.00000000000000
C        0.61173202483911      -2.91170474553767       0.00000000000000
F       -0.51311191297493      -3.71638678568524       0.00000000000000
F        1.34243603193623      -3.28610506872096       1.10560749173682
F        1.34243603193623      -3.28610506872096      -1.10560749173682
F       -1.34243603193623       3.28610506872096      -1.10560749173682
F        0.51311191297493       3.71638678568524       0.00000000000000
F       -1.34243603193623       3.28610506872096       1.10560749173682
```

The triplet-optimized C_{2h} geometry of molecule **PD** is given below (BLYP/TZP level of theory).
Total bonding energy: -11.524607282272729 *a.u.*

```
46
C26H8F6N6 optimized with ADF in AMS.
C       -3.29310127961638       -0.77470184071994       0.00000000000000
C       -2.79394233729564       0.56882719881886        0.00000000000000
N       -2.21773658552256       -1.65053418510688       0.00000000000000
C       -4.64866104497290       -1.08257336379808       0.00000000000000
C       -3.73915640470531       1.62592455820614        0.00000000000000
C       -5.09348787394155       1.33989538317758        0.00000000000000
C       -5.57778797649392       -0.00981204952416       0.00000000000000
N       8.73557420199592        -1.64322267710879       0.00000000000000
H       -4.99341322970905       -2.11492240881930       0.00000000000000
H       3.42208300463835        -2.66225526033626       0.00000000000000
C       -1.34434989150192       0.47445163103487        0.00000000000000
H       -3.42208300463835       2.66225526033626        0.00000000000000
H       -5.81250996775255       2.15593883823001        0.00000000000000
C       -1.02477802690362       -0.94016359427758       0.00000000000000
C       0.29417949064806        -1.42711398105817       0.00000000000000
C       1.34434989150192        -0.47445163103487       0.00000000000000
C       1.02477802690362        0.94016359427758        0.00000000000000
C       -0.29417949064806       1.42711398105817        0.00000000000000
H       -2.28248316946146       -2.66023386441174       0.00000000000000
H       2.28248316946146        2.66023386441174        0.00000000000000
C       2.79394233729564        -0.56882719881886       0.00000000000000
C       3.29310127961638        0.77470184071994        0.00000000000000
N       2.21773658552256        1.65053418510688        0.00000000000000
N       7.88336094039025        2.71086861634696        0.00000000000000
C       3.73915640470531        -1.62592455820614       0.00000000000000
C       5.09348787394155        -1.33989538317758       0.00000000000000
C       5.57778797649392        0.00981204952416        0.00000000000000
C       4.64866104497290        1.08257336379808        0.00000000000000
N       -7.88336094039025       -2.71086861634696       0.00000000000000
H       5.81250996775255        -2.15593883823001       0.00000000000000
H       4.99341322970905        2.11492240881930        0.00000000000000
C       -6.99967493478877       -0.27725403969193       0.00000000000000
C       -7.95535922124443       0.76925258990152        0.00000000000000
C       -7.49967334955467       -1.60402235743744       0.00000000000000
N       -8.73557420199592       1.64322267710879        0.00000000000000
```

```
C       6.99967493478877        0.27725403969193        0.00000000000000
C       7.95535922124443        -0.76925258990152       0.00000000000000
C       7.49967334955467        1.60402235743744        0.00000000000000
C       -0.60942628207226       2.90614072396254        0.00000000000000
C       0.60942628207226        -2.90614072396254       0.00000000000000
F       -0.51757710167070       -3.70772764243273       0.00000000000000
F       1.33773815558822        -3.28249655974315       1.10579518279646
F       1.33773815558822        -3.28249655974315       -1.10579518279646
F       -1.33773815558822       3.28249655974315        -1.10579518279646
F       0.51757710167070        3.70772764243273        0.00000000000000
F       -1.33773815558822       3.28249655974315        1.10579518279646
```

The closed-shell singlet-optimized C_{2h} geometry of molecule **PD1** is given below (BLYP/TZP level of theory). Total bonding energy: -11.752603862989492 *a.u.*

```
46
C26H12N8 optimized with ADF in AMS.
C       -3.29915139782421       -0.67174996357442       0.00000000000000
C       -2.75174269699137       0.66930333647555        0.00000000000000
N       -2.23980228197530       -1.57305699785507       0.00000000000000
C       -4.65104806476806       -0.93916787902334       0.00000000000000
C       -3.68095677668581       1.75341823963850        0.00000000000000
C       -5.03481464532014       1.50846880199982        0.00000000000000
C       -5.56441627381685       0.16467156934098        0.00000000000000
N       8.65109900040343        -1.91456136365603       0.00000000000000
H       -5.02559634995634       -1.96089956071582       0.00000000000000
H       3.34034281839595        -2.78515689008178       0.00000000000000
C       -1.33092409565376       0.54577726887207        0.00000000000000
H       -3.34034281839595       2.78515689008178        0.00000000000000
H       -5.73359075753292       2.34186236387563        0.00000000000000
C       -1.05005178676549       -0.88905604014754       0.00000000000000
C       0.23459777725179        -1.46823443307250       0.00000000000000
C       1.33092409565376        -0.54577726887207       0.00000000000000
C       1.05005178676549        0.88905604014754        0.00000000000000
C       -0.23459777725179       1.46823443307250        0.00000000000000
H       -2.23814080829135       -2.60031173230123       0.00000000000000
H       2.23814080829135        2.60031173230123        0.00000000000000
C       2.75174269699137        -0.66930333647555       0.00000000000000
C       3.29915139782421        0.67174996357442        0.00000000000000
N       2.23980228197530        1.57305699785507        0.00000000000000
N       7.93770183326525        2.46379797318772        0.00000000000000
C       3.68095677668581        -1.75341823963850       0.00000000000000
C       5.03481464532014        -1.50846880199982       0.00000000000000
C       5.56441627381685        -0.16467156934098       0.00000000000000
C       4.65104806476806        0.93916787902334        0.00000000000000
N       -7.93770183326525       -2.46379797318772       0.00000000000000
H       5.73359075753292        -2.34186236387563       0.00000000000000
H       5.02559634995634        1.96089956071582        0.00000000000000
C       -6.97088924909908       -0.05893197702384       0.00000000000000
C       -7.89884577333388       1.01800847345306        0.00000000000000
C       -7.51374857421742       -1.37295692912513       0.00000000000000
N       -8.65109900040343       1.91456136365603        0.00000000000000
C       6.97088924909908        0.05893197702384        0.00000000000000
C       7.89884577333388        -1.01800847345306       0.00000000000000
C       7.51374857421742        1.37295692912513        0.00000000000000
C       -0.40131015196974       2.92173880965785        0.00000000000000
N       0.60742274816963        3.73794994825618        0.00000000000000
C       0.40131015196974        -2.92173880965785       0.00000000000000
```

N	-0.60742274816963	-3.73794994825618	0.00000000000000
H	-0.29252757479612	-4.71521257190833	0.00000000000000
H	-1.43359621503723	3.28891496536368	0.00000000000000
H	1.43359621503723	-3.28891496536368	0.00000000000000
H	0.29252757479612	4.71521257190833	0.00000000000000

The triplet-optimized C_{2h} geometry of molecule **PD1** is given below (BLYP/TZP level of theory).
Total bonding energy: -11.745659174511584 $a.u.$

46
C26H12N8 optimized with ADF in AMS.

C	-3.30686667804886	-0.66384102327337	0.00000000000000
C	-2.76978703804657	0.67205139434403	0.00000000000000
N	-2.25276607281736	-1.56455984408976	0.00000000000000
C	-4.67063597249004	-0.93038074849605	0.00000000000000
C	-3.69006089660993	1.75076924108942	0.00000000000000
C	-5.05260562597008	1.50430036927197	0.00000000000000
C	-5.57248486047027	0.16750710870617	0.00000000000000
N	8.67481825318918	-1.91375877606458	0.00000000000000
H	-5.04333254413677	-1.95283479011498	0.00000000000000
H	3.35109124392377	-2.78293165867295	0.00000000000000
C	-1.32559684883296	0.54151424606173	0.00000000000000
H	-3.35109124392377	2.78293165867295	0.00000000000000
H	-5.74947507462640	2.33921794091468	0.00000000000000
C	-1.05707152279070	-0.87857459253191	0.00000000000000
C	0.23541917575370	-1.45891789016117	0.00000000000000
C	1.32559684883296	-0.54151424606173	0.00000000000000
C	1.05707152279070	0.87857459253191	0.00000000000000
C	-0.23541917575370	1.45891789016117	0.00000000000000
H	-2.25452088186256	-2.59021504069979	0.00000000000000
H	2.25452088186256	2.59021504069979	0.00000000000000
C	2.76978703804657	-0.67205139434403	0.00000000000000
C	3.30686667804886	0.66384102327337	0.00000000000000
N	2.25276607281736	1.56455984408976	0.00000000000000
N	7.95977164805318	2.46544463295830	0.00000000000000
C	3.69006089660993	-1.75076924108942	0.00000000000000
C	5.05260562597008	-1.50430036927197	0.00000000000000
C	5.57248486047027	-0.16750710870617	0.00000000000000
C	4.67063597249004	0.93038074849605	0.00000000000000
N	-7.95977164805318	-2.46544463295830	0.00000000000000
H	5.74947507462640	-2.33921794091468	0.00000000000000
H	5.04333254413677	1.95283479011498	0.00000000000000
C	-7.00053238135112	-0.06074647055499	0.00000000000000
C	-7.92477299973133	1.01388149602164	0.00000000000000
C	-7.53927955491460	-1.37213920335015	0.00000000000000
N	-8.67481825318918	1.91375877606458	0.00000000000000
C	7.00053238135112	0.06074647055499	0.00000000000000
C	7.92477299973133	-1.01388149602164	0.00000000000000
C	7.53927955491460	1.37213920335015	0.00000000000000
C	-0.40529642577527	2.91303611637834	0.00000000000000
N	0.60173156790651	3.73009733119011	0.00000000000000
C	0.40529642577527	-2.91303611637834	0.00000000000000
N	-0.60173156790651	-3.73009733119011	0.00000000000000
H	-0.28547759070064	-4.70685804279420	0.00000000000000
H	-1.43738289224135	3.27951612747947	0.00000000000000
H	1.43738289224135	-3.27951612747947	0.00000000000000
H	0.28547759070064	4.70685804279420	0.00000000000000

The closed-shell singlet-optimized C_{2h} geometry of molecule **PD2** is given below (BLYP/TZP level of theory).
Total bonding energy: -10.321822890528704 *a.u.*

```
40
C24H8F2N6 optimized with ADF in AMS.
C       3.29335533740136        0.75723992488991        0.00000000000000
C       2.75446825058561        -0.58519279874921       0.00000000000000
N       2.22538639621865        1.65494481976615        0.00000000000000
C       4.64981836781302        1.00980121754508        0.00000000000000
C       3.65515616976206        -1.68632721310329       0.00000000000000
C       5.01082911227703        -1.44894477207412       0.00000000000000
C       5.54754172670767        -0.10567811150423       0.00000000000000
N       -8.62335274757939       1.88430602113987        0.00000000000000
H       5.04143820257182        2.02516496045657        0.00000000000000
H       -3.27328650332932       2.70461334955529        0.00000000000000
C       1.33896565947758        -0.47243711234717       0.00000000000000
H       3.27328650332932        -2.70461334955529       0.00000000000000
H       5.70695252403732        -2.28466640896263       0.00000000000000
C       1.02772628126761        0.94839260754771        0.00000000000000
C       -0.27574857364147       1.40099573049190        0.00000000000000
C       -1.33896565947758       0.47243711234717        0.00000000000000
C       -1.02772628126761       -0.94839260754771       0.00000000000000
C       0.27574857364147        -1.40099573049190       0.00000000000000
H       -2.29955465218297       -2.66582119746847       0.00000000000000
H       2.29955465218297        2.66582119746847        0.00000000000000
C       -2.75446825058561       0.58519279874921        0.00000000000000
C       -3.29335533740136       -0.75723992488991       0.00000000000000
N       -2.22538639621865       -1.65494481976615       0.00000000000000
N       -7.93875969815035       -2.50099887138765       0.00000000000000
C       -3.65515616976206       1.68632721310329        0.00000000000000
C       -5.01082911227703       1.44894477207412        0.00000000000000
C       -5.54754172670767       0.10567811150423        0.00000000000000
C       -4.64981836781302       -1.00980121754508       0.00000000000000
N       7.93875969815035        2.50099887138765        0.00000000000000
H       -5.70695252403732       2.28466640896263        0.00000000000000
H       -5.04143820257182       -2.02516496045657       0.00000000000000
C       6.95881822451838        0.10185514179870        0.00000000000000
C       7.87673353861747        -0.98281823843934       0.00000000000000
C       7.51210273893796        1.41096326772159        0.00000000000000
N       8.62335274757939        -1.88430602113987       0.00000000000000
C       -6.95881822451838       -0.10185514179870       0.00000000000000
C       -7.87673353861747       0.98281823843934        0.00000000000000
C       -7.51210273893796       -1.41096326772159       0.00000000000000
F       0.53483666198791        -2.75213575898035       0.00000000000000
F       -0.53483666198791       2.75213575898035        0.00000000000000
```

The triplet-optimized C_{2h} geometry of molecule **PD2** is given below (BLYP/TZP level of theory).
Total bonding energy: -10.316529623901287 *a.u.*

```
40
C24H8F2N6 optimized with ADF in AMS.
C        3.29935828570592        0.74276164090840       0.00000000000000
C        2.77159479699937       -0.59368441215080       0.00000000000000
N        2.23433710594708        1.64149462922883       0.00000000000000
C        4.66522498146235        0.99752695664359       0.00000000000000
C        3.66551711408109       -1.68762408812897       0.00000000000000
C        5.03024870711359       -1.44799135142610       0.00000000000000
C        5.55503190712216       -0.11204485144447       0.00000000000000
```

N	-8.65225692396869	1.87447985398802	0.00000000000000
H	5.05321270706588	2.01434714649888	0.00000000000000
H	-3.28682666715604	2.70711073958748	0.00000000000000
C	1.33400369141722	-0.47724554367534	0.00000000000000
H	3.28682666715604	-2.70711073958748	0.00000000000000
H	5.72508070735840	-2.28464652093206	0.00000000000000
C	1.03457939900406	0.93408956215992	0.00000000000000
C	-0.27532287844692	1.39619987341887	0.00000000000000
C	-1.33400369141722	0.47724554367534	0.00000000000000
C	-1.03457939900406	-0.93408956215992	0.00000000000000
C	0.27532287844692	-1.39619987341887	0.00000000000000
H	-2.30843351220784	-2.65212445100902	0.00000000000000
H	2.30843351220784	2.65212445100902	0.00000000000000
C	-2.77159479699937	0.59368441215080	0.00000000000000
C	-3.29935828570592	-0.74276164090840	0.00000000000000
N	-2.23433710594708	-1.64149462922883	0.00000000000000
N	-7.95180520070323	-2.50828639338388	0.00000000000000
C	-3.66551711408109	1.68762408812897	0.00000000000000
C	-5.03024870711359	1.44799135142610	0.00000000000000
C	-5.55503190712216	0.11204485144447	0.00000000000000
C	-4.66522498146235	-0.99752695664359	0.00000000000000
N	7.95180520070323	2.50828639338388	0.00000000000000
H	-5.72508070735840	2.28464652093206	0.00000000000000
H	-5.05321270706588	-2.01434714649888	0.00000000000000
C	6.98591983787513	0.10641366469585	0.00000000000000
C	7.90452167572992	-0.97269767145686	0.00000000000000
C	7.53092248005406	1.41506802709861	0.00000000000000
N	8.65225692396869	-1.87447985398802	0.00000000000000
C	-6.98591983787513	-0.10641366469585	0.00000000000000
C	-7.90452167572992	0.97269767145686	0.00000000000000
C	-7.53092248005406	-1.41506802709861	0.00000000000000
F	0.52001797234679	-2.75083463467515	0.00000000000000
F	-0.52001797234679	2.75083463467515	0.00000000000000

The closed-shell singlet-optimized C_{2h} geometry of molecule **PD3** is given below (BLYP/TZP level of theory). Total bonding energy: -11.886288929825877 $a.u.$

48
C26H14N6O2 optimized with ADF in AMS.

C	-3.35065550841116	-0.47819443309904	0.00000000000000
C	-2.68049273595207	0.80184710572896	0.00000000000000
N	-2.38041576664900	-1.46895332820382	0.00000000000000
C	-4.72734246843283	-0.60489856098370	0.00000000000000
C	-3.48097074317810	1.98189544924820	0.00000000000000
C	-4.85212001579828	1.87031040494407	0.00000000000000
C	-5.51691436606309	0.58429119027424	0.00000000000000
N	8.40728116481205	-2.65576765515924	0.00000000000000
H	-5.20975232105691	-1.58057782007101	0.00000000000000
H	3.00729650809432	-2.95856487536076	0.00000000000000
C	-1.28295518064461	0.54990878721798	0.00000000000000
H	-3.00729650809432	2.95856487536076	0.00000000000000
H	-5.46636158474319	2.76822312019730	0.00000000000000
C	-1.09704430547617	-0.88982151006816	0.00000000000000
C	0.17334686412865	-1.45740819279458	0.00000000000000
C	1.28295518064461	-0.54990878721798	0.00000000000000
C	1.09704430547617	0.88982151006816	0.00000000000000
C	-0.17334686412865	1.45740819279458	0.00000000000000
H	-2.58300039711352	-2.45818358970567	0.00000000000000

```
H       2.58300039711352        2.45818358970567        0.00000000000000
C       2.68049273595207        -0.80184710572896       0.00000000000000
C       3.35065550841116        0.47819443309904        0.00000000000000
N       2.38041576664900        1.46895332820382        0.00000000000000
N       8.13735780316007        1.78273858202061        0.00000000000000
C       3.48097074317810        -1.98189544924820       0.00000000000000
C       4.85212001579828        -1.87031040494407       0.00000000000000
C       5.51691436606309        -0.58429119027424       0.00000000000000
C       4.72734246843283        0.60489856098370        0.00000000000000
N       -8.13735780316007       -1.78273858202061       0.00000000000000
H       5.46636158474319        -2.76822312019730       0.00000000000000
H       5.20975232105691        1.58057782007101        0.00000000000000
C       -6.94352773529502       0.51723818527081        0.00000000000000
C       -7.75104540845568       1.68583446841708        0.00000000000000
C       -7.61778857898112       -0.73317234989392       0.00000000000000
N       -8.40728116481205       2.65576765515924        0.00000000000000
C       6.94352773529502        -0.51723818527081       0.00000000000000
C       7.75104540845568        -1.68583446841708       0.00000000000000
C       7.61778857898112        0.73317234989392        0.00000000000000
O       -0.53142789172023       2.78886982926664        0.00000000000000
O       0.53142789172023        -2.78886982926664       0.00000000000000
C       -0.47116549795281       -3.83122729579471       0.00000000000000
C       0.47116549795281        3.83122729579471        0.00000000000000
H       -1.08852175230532       -3.78869864345499       0.90762151393099
H       0.08926351802896        -4.76938456745110       0.00000000000000
H       -1.08852175230532       -3.78869864345499       -0.90762151393099
H       -0.08926351802896       4.76938456745110        0.00000000000000
H       1.08852175230532        3.78869864345499        -0.90762151393099
H       1.08852175230532        3.78869864345499        0.90762151393099
```

The triplet-optimized C_{2h} geometry of molecule **PD3** is given below (BLYP/TZP level of theory).
Total bonding energy: -11.878825743938210 *a.u.*

```
48
C26H14N6O2 optimized with ADF in AMS.
C       -3.35351235225490       -0.46364030463340       0.00000000000000
C       -2.69589766569376       0.81191285228979        0.00000000000000
N       -2.38458861158258       -1.45572859858155       0.00000000000000
C       -4.73801701401826       -0.59301984273555       0.00000000000000
C       -3.49067631888534       1.98362424827570        0.00000000000000
C       -4.87126687428952       1.87133377693058        0.00000000000000
C       -5.52239630793332       0.59115915958952        0.00000000000000
N       8.44232281239325        -2.63793845153990       0.00000000000000
H       -5.21707811010068       -1.57044362032062       0.00000000000000
H       3.01922220090131        -2.96155642596623       0.00000000000000
C       -1.27702506534506       0.55537783600624        0.00000000000000
H       -3.01922220090131       2.96155642596623        0.00000000000000
H       -5.48477404613650       2.76956008163160        0.00000000000000
C       -1.10099773736330       -0.87582538527862       0.00000000000000
C       0.17671925367124        -1.45329605679906       0.00000000000000
C       1.27702506534506        -0.55537783600624       0.00000000000000
C       1.10099773736330        0.87582538527862        0.00000000000000
C       -0.17671925367124       1.45329605679906        0.00000000000000
H       -2.58696983484209       -2.44474739539131       0.00000000000000
H       2.58696983484209        2.44474739539131        0.00000000000000
C       2.69589766569376        -0.81191285228979       0.00000000000000
C       3.35351235225490        0.46364030463340        0.00000000000000
N       2.38458861158258        1.45572859858155        0.00000000000000
```

```
N       8.14494049341173         1.79519350150658         0.00000000000000
C       3.49067631888534        -1.98362424827570         0.00000000000000
C       4.87126687428952        -1.87133377693058         0.00000000000000
C       5.52239630793332        -0.59115915958952         0.00000000000000
C       4.73801701401826         0.59301984273555         0.00000000000000
N      -8.14494049341173        -1.79519350150658         0.00000000000000
H       5.48477404613650        -2.76956008163160         0.00000000000000
H       5.21707811010068         1.57044362032062         0.00000000000000
C      -6.96713893766105         0.51049523829863         0.00000000000000
C      -7.78039453150462         1.67093179742039         0.00000000000000
C      -7.63108966303227        -0.74197245060515         0.00000000000000
N      -8.44232281239325         2.63793845153990         0.00000000000000
C       6.96713893766105        -0.51049523829863         0.00000000000000
C       7.78039453150462        -1.67093179742039         0.00000000000000
C       7.63108966303227         0.74197245060515         0.00000000000000
O      -0.52249048736674         2.79147073280141         0.00000000000000
O       0.52249048736674        -2.79147073280141         0.00000000000000
C      -0.48910618907581        -3.82308487918574         0.00000000000000
C       0.48910618907581         3.82308487918574         0.00000000000000
H      -1.10687745303666        -3.77641421460098         0.90749913513865
H       0.06212250506741        -4.76684471998676         0.00000000000000
H      -1.10687745303666        -3.77641421460098        -0.90749913513865
H      -0.06212250506741         4.76684471998676         0.00000000000000
H       1.10687745303666         3.77641421460098        -0.90749913513865
H       1.10687745303666         3.77641421460098         0.90749913513865
```

The closed-shell singlet-optimized C_{2h} geometry of molecule **PAH** is given below (BLYP/TZP level of theory). Total bonding energy: -11.702338656325288 *a.u.*

```
46
C28H12N6 optimized with ADF in AMS.
C        3.30453299915454       0.75957994984797       0.00000000000000
C        2.77709047118348      -0.58163560668413       0.00000000000000
N        2.21624722200560       1.63398598867113       0.00000000000000
C        4.65712065248041       1.03992737849391       0.00000000000000
C        3.69921553082676      -1.66427407182207       0.00000000000000
C        5.05409254457829      -1.40456667338127       0.00000000000000
C        5.57213669071078      -0.05896220187893       0.00000000000000
N       -8.67980681492181       1.78007804924931       0.00000000000000
H        5.02906023556251       2.06286538856312       0.00000000000000
H       -3.34061745700893       2.69021376161272       0.00000000000000
C        1.35607372240206      -0.47134032586976       0.00000000000000
H        3.34061745700893      -2.69021376161272       0.00000000000000
H        5.76142056402716      -2.23093476168668       0.00000000000000
C        1.04468402102343       0.90969094805962       0.00000000000000
C       -0.29575105348103       1.48729836592366       0.00000000000000
C       -1.35607372240206       0.47134032586976       0.00000000000000
C       -1.04468402102343      -0.90969094805962       0.00000000000000
C        0.29575105348103      -1.48729836592366       0.00000000000000
H        2.26244759469639       2.64801749235890       0.00000000000000
H       -2.26244759469639      -2.64801749235890       0.00000000000000
C       -2.77709047118348       0.58163560668413       0.00000000000000
C       -3.30453299915454      -0.75957994984797       0.00000000000000
N       -2.21624722200560      -1.63398598867113       0.00000000000000
N       -7.91126739603246      -2.59809096720350       0.00000000000000
C       -3.69921553082676       1.66427407182207       0.00000000000000
C       -5.05409254457829       1.40456667338127       0.00000000000000
C       -5.57213669071078       0.05896220187893       0.00000000000000
```

C	-4.65712065248041	-1.03992737849391	0.00000000000000
N	7.91126739603246	2.59809096720350	0.00000000000000
H	-5.76142056402716	2.23093476168668	0.00000000000000
H	-5.02906023556251	-2.06286538856312	0.00000000000000
C	6.98583863019711	0.17897581859945	0.00000000000000
C	7.92128440096846	-0.88767009962898	0.00000000000000
C	7.51086883056528	1.49737267907868	0.00000000000000
N	8.67980681492181	-1.78007804924931	0.00000000000000
C	-6.98583863019711	-0.17897581859945	0.00000000000000
C	-7.92128440096846	0.88767009962898	0.00000000000000
C	-7.51086883056528	-1.49737267907868	0.00000000000000
C	0.48236906612222	-2.81011336335166	0.00000000000000
C	-0.48236906612222	2.81011336335166	0.00000000000000
C	0.60026148747612	-4.11174236378001	0.00000000000000
C	-0.60026148747612	4.11174236378001	0.00000000000000
H	-0.66257692975991	4.68631019913736	0.92990611586748
H	-0.66257692975991	4.68631019913736	-0.92990611586748
H	0.66257692975991	-4.68631019913736	-0.92990611586748
H	0.66257692975991	-4.68631019913736	0.92990611586748

The triplet-optimized C_{2h} geometry of molecule **PAH** is given below (BLYP/TZP level of theory).
Total bonding energy: -11.708235116595144 $a.u.$

46
C28H12N6 optimized with ADF in AMS.

C	3.31372568746208	0.75652971094132	0.00000000000000
C	2.78923886028958	-0.58203542341098	0.00000000000000
N	2.22799958686922	1.63122844286483	0.00000000000000
C	4.67134992611402	1.03772915546712	0.00000000000000
C	3.71027594140690	-1.66153393148894	0.00000000000000
C	5.06773336797409	-1.39984127914734	0.00000000000000
C	5.58175368559321	-0.05670311739768	0.00000000000000
N	-8.69596106103330	1.77903084081078	0.00000000000000
H	5.04105442146302	2.06155844213524	0.00000000000000
H	-3.35515992054238	2.68881918822726	0.00000000000000
C	1.36102548692432	-0.46991149672878	0.00000000000000
H	3.35515992054238	-2.68881918822726	0.00000000000000
H	5.77475559160860	-2.22657661356590	0.00000000000000
C	1.05452086580758	0.90611979387040	0.00000000000000
C	-0.29209288817790	1.47726737055845	0.00000000000000
C	-1.36102548692432	0.46991149672878	0.00000000000000
C	-1.05452086580758	-0.90611979387040	0.00000000000000
C	0.29209288817790	-1.47726737055845	0.00000000000000
H	2.27705656901845	2.64485303476621	0.00000000000000
H	-2.27705656901845	-2.64485303476621	0.00000000000000
C	-2.78923886028958	0.58203542341098	0.00000000000000
C	-3.31372568746208	-0.75652971094132	0.00000000000000
N	-2.22799958686922	-1.63122844286483	0.00000000000000
N	-7.92848098616226	-2.60103575602829	0.00000000000000
C	-3.71027594140690	1.66153393148894	0.00000000000000
C	-5.06773336797409	1.39984127914734	0.00000000000000
C	-5.58175368559321	0.05670311739768	0.00000000000000
C	-4.67134992611402	-1.03772915546712	0.00000000000000
N	7.92848098616226	2.60103575602829	0.00000000000000
H	-5.77475559160860	2.22657661356590	0.00000000000000
H	-5.04105442146302	-2.06155844213524	0.00000000000000
C	7.00534419022499	0.18184284391609	0.00000000000000
C	7.93891227382056	-0.88500873351379	0.00000000000000

```
C        7.52943386069311      1.49962700397671       0.00000000000000
N        8.69596106103330     -1.77903084081078       0.00000000000000
C       -7.00534419022499     -0.18184284391609       0.00000000000000
C       -7.93891227382056      0.88500873351379       0.00000000000000
C       -7.52943386069311     -1.49962700397671       0.00000000000000
C        0.47493961601221     -2.80105243809717       0.00000000000000
C       -0.47493961601221      2.80105243809717       0.00000000000000
C        0.59268245772313     -4.10156342673329       0.00000000000000
C       -0.59268245772313      4.10156342673329       0.00000000000000
H       -0.65479310372654      4.67465163840496       0.93049264954420
H       -0.65479310372654      4.67465163840496      -0.93049264954420
H        0.65479310372654     -4.67465163840496      -0.93049264954420
H        0.65479310372654     -4.67465163840496       0.93049264954420
```

The closed-shell singlet-optimized C_{2h} geometry of molecule **PA** is given below (BLYP/TZP level of theory). Total bonding energy: -13.428683548223836 $a.u.$

```
50
C32H8N10 optimized with ADF in AMS.
C       3.30902603000000       0.75250199000000        0.00000000000000
C       2.78215350000000       -0.58640087000000       0.00000000000000
N       2.22113871000000       1.63029989000000        0.00000000000000
C       4.66167153000000       1.03602987000000        0.00000000000000
C       3.70300801000000       -1.66959924000000       0.00000000000000
C       5.05674249000000       -1.40900248000000       0.00000000000000
C       5.57484160000000       -0.06274421000000       0.00000000000000
N       -8.67962582000000      1.78677241000000        0.00000000000000
H       5.03195375000000       2.05945128000000        0.00000000000000
H       -3.35209613000000      2.69824044000000        0.00000000000000
C       1.36089962000000       -0.47036689000000       0.00000000000000
H       3.35209613000000       -2.69824044000000       0.00000000000000
H       5.76329516000000       -2.23591803000000       0.00000000000000
C       1.05081780000000       0.91021842000000        0.00000000000000
C       -0.29687800000000      1.47687823000000        0.00000000000000
C       -1.36089962000000      0.47036689000000        0.00000000000000
C       -1.05081780000000      -0.91021842000000       0.00000000000000
C       0.29687800000000       -1.47687823000000       0.00000000000000
H       2.28369961000000       2.64382807000000        0.00000000000000
H       -2.28369961000000      -2.64382807000000       0.00000000000000
C       -2.78215350000000      0.58640087000000        0.00000000000000
C       -3.30902603000000      -0.75250199000000       0.00000000000000
N       -2.22113871000000      -1.63029989000000       0.00000000000000
N       -7.91183146000000      -2.59280738000000       0.00000000000000
C       -3.70300801000000      1.66959924000000        0.00000000000000
C       -5.05674249000000      1.40900248000000        0.00000000000000
C       -5.57484160000000      0.06274421000000        0.00000000000000
C       -4.66167153000000      -1.03602987000000       0.00000000000000
N       7.91183146000000       2.59280738000000        0.00000000000000
H       -5.76329516000000      2.23591803000000        0.00000000000000
H       -5.03195375000000      -2.05945128000000       0.00000000000000
C       6.98799053000000       0.17359669000000        0.00000000000000
C       7.92211473000000       -0.89399298000000       0.00000000000000
C       7.51300419000000       1.49203309000000        0.00000000000000
N       8.67962582000000       -1.78677241000000       0.00000000000000
C       -6.98799053000000      -0.17359669000000       0.00000000000000
C       -7.92211473000000      0.89399298000000        0.00000000000000
C       -7.51300419000000      -1.49203309000000       0.00000000000000
C       0.49459486000000       -2.79467044000000       0.00000000000000
```

C	-0.49459486000000	2.79467044000000	0.00000000000000
C	0.62767792000000	-4.11567962000000	0.00000000000000
C	-0.62767792000000	4.11567962000000	0.00000000000000
N	-0.76471920000000	5.45228585000000	2.24127797000000
N	-0.76471920000000	5.45228585000000	-2.24127797000000
N	0.76471920000000	-5.45228585000000	2.24127797000000
N	0.76471920000000	-5.45228585000000	-2.24127797000000
C	-0.70747831000000	4.86395897000000	1.23703990000000
C	-0.70747831000000	4.86395897000000	-1.23703990000000
C	0.70747831000000	-4.86395897000000	-1.23703990000000
C	0.70747831000000	-4.86395897000000	1.23703990000000

The triplet-optimized C_{2h} geometry of molecule **PA** is given below (BLYP/TZP level of theory).
Total bonding energy: -13.433207902556621 $a.u.$

50
C32H8N10 optimized with ADF in AMS.

C	3.31519685000000	0.75356099000000	0.00000000000000
C	2.79257677000000	-0.58311749000000	0.00000000000000
N	2.23108395000000	1.63056282000000	0.00000000000000
C	4.67478899000000	1.03564655000000	0.00000000000000
C	3.70955139000000	-1.66411391000000	0.00000000000000
C	5.06697779000000	-1.40250509000000	0.00000000000000
C	5.57993912000000	-0.05960279000000	0.00000000000000
N	-8.69305640000000	1.78633185000000	0.00000000000000
H	5.04442996000000	2.05938167000000	0.00000000000000
H	-3.36020055000000	2.69331287000000	0.00000000000000
C	1.36225520000000	-0.46568416000000	0.00000000000000
H	3.36020055000000	-2.69331287000000	0.00000000000000
H	5.77254368000000	-2.23030273000000	0.00000000000000
C	1.05762574000000	0.90796831000000	0.00000000000000
C	-0.29543825000000	1.47221130000000	0.00000000000000
C	-1.36225520000000	0.46568416000000	0.00000000000000
C	-1.05762574000000	-0.90796831000000	0.00000000000000
C	0.29543825000000	-1.47221130000000	0.00000000000000
H	2.29445227000000	2.64372270000000	0.00000000000000
H	-2.29445227000000	-2.64372270000000	0.00000000000000
C	-2.79257677000000	0.58311749000000	0.00000000000000
C	-3.31519685000000	-0.75356099000000	0.00000000000000
N	-2.23108395000000	-1.63056282000000	0.00000000000000
N	-7.93118680000000	-2.59445558000000	0.00000000000000
C	-3.70955139000000	1.66411391000000	0.00000000000000
C	-5.06697779000000	1.40250509000000	0.00000000000000
C	-5.57993912000000	0.05960279000000	0.00000000000000
C	-4.67478899000000	-1.03564655000000	0.00000000000000
N	7.93118680000000	2.59445558000000	0.00000000000000
H	-5.77254368000000	2.23030273000000	0.00000000000000
H	-5.04442996000000	-2.05938167000000	0.00000000000000
C	7.00627116000000	0.17673653000000	0.00000000000000
C	7.93729718000000	-0.89156868000000	0.00000000000000
C	7.53157745000000	1.49361600000000	0.00000000000000
N	8.69305640000000	-1.78633185000000	0.00000000000000
C	-7.00627116000000	-0.17673653000000	0.00000000000000
C	-7.93729718000000	0.89156868000000	0.00000000000000
C	-7.53157745000000	-1.49361600000000	0.00000000000000
C	0.49120077000000	-2.78878470000000	0.00000000000000
C	-0.49120077000000	2.78878470000000	0.00000000000000
C	0.62222623000000	-4.11013565000000	0.00000000000000

```
C       -0.62222623000000       4.11013565000000        0.00000000000000
N       -0.75809248000000       5.44888044000000        2.23821573000000
N       -0.75809248000000       5.44888044000000       -2.23821573000000
N       0.75809248000000       -5.44888044000000        2.23821573000000
N       0.75809248000000       -5.44888044000000       -2.23821573000000
C       -0.70136276000000       4.85759732000000        1.23583248000000
C       -0.70136276000000       4.85759732000000       -1.23583248000000
C       0.70136276000000       -4.85759732000000       -1.23583248000000
C       0.70136276000000       -4.85759732000000        1.23583248000000
```

The closed-shell singlet-optimized C_{2h} geometry of molecule **PF** is given below (BLYP/TZP level of theory). Total bonding energy: -11.710767147474160 *a.u.*

```
46
C28H8F4N6 optimized with ADF in AMS.
C        3.30163236771529       0.74940211135120       0.00000000000000
C        2.77549000186140      -0.59153063043488       0.00000000000000
N        2.21205951835165       1.62490430581005       0.00000000000000
C        4.65327486872477       1.03380230694408       0.00000000000000
C        3.69788826979959      -1.67406929772973       0.00000000000000
C        5.05128744359882      -1.41180317683267       0.00000000000000
C        5.56860636214279      -0.06483301451505       0.00000000000000
N       -8.67710486606172       1.78297252984255       0.00000000000000
H        5.02312663165988       2.05740163146091       0.00000000000000
H       -3.34141460177541       2.70052375515438       0.00000000000000
C        1.35520513907122      -0.47931876870829       0.00000000000000
H        3.34141460177541      -2.70052375515438       0.00000000000000
H        5.75944452973155      -2.23742624362835       0.00000000000000
C        1.04424000317145       0.90609778722681       0.00000000000000
C       -0.29541244527348       1.48535838869173       0.00000000000000
C       -1.35520513907122       0.47931876870829       0.00000000000000
C       -1.04424000317145      -0.90609778722681       0.00000000000000
C        0.29541244527348      -1.48535838869173       0.00000000000000
H        2.25834160251138       2.63987155294522       0.00000000000000
H       -2.25834160251138      -2.63987155294522       0.00000000000000
C       -2.77549000186140       0.59153063043488       0.00000000000000
C       -3.30163236771529      -0.74940211135120       0.00000000000000
N       -2.21205951835165      -1.62490430581005       0.00000000000000
N       -7.90409912534240      -2.59362136633071       0.00000000000000
C       -3.69788826979959       1.67406929772973       0.00000000000000
C       -5.05128744359882       1.41180317683267       0.00000000000000
C       -5.56860636214279       0.06483301451505       0.00000000000000
C       -4.65327486872477      -1.03380230694408       0.00000000000000
N        7.90409912534240       2.59362136633071       0.00000000000000
H       -5.75944452973155       2.23742624362835       0.00000000000000
H       -5.02312663165988      -2.05740163146091       0.00000000000000
C        6.98053337683584       0.17377542216060       0.00000000000000
C        7.91706708097558      -0.89228457947407       0.00000000000000
C        7.50459612511237       1.49295292661584       0.00000000000000
N        8.67710486606172      -1.78297252984255       0.00000000000000
C       -6.98053337683584      -0.17377542216060       0.00000000000000
C       -7.91706708097558       0.89228457947407       0.00000000000000
C       -7.50459612511237      -1.49295292661584       0.00000000000000
C        0.48817258734928      -2.81281844448141       0.00000000000000
C       -0.48817258734928       2.81281844448141       0.00000000000000
C        0.62609404854920      -4.10215953348176       0.00000000000000
C       -0.62609404854920       4.10215953348176       0.00000000000000
F       -0.71986207850521       4.89112916058128       1.10360508026704
```

F	-0.71986207850521	4.89112916058128	-1.10360508026704
F	0.71986207850521	-4.89112916058128	-1.10360508026704
F	0.71986207850521	-4.89112916058128	1.10360508026704

The triplet-optimized C_{2h} geometry of molecule **PF** is given below (BLYP/TZP level of theory).
Total bonding energy: -11.713815910175793 *a.u.*

46
C28H8F4N6 optimized with ADF in AMS.

C	3.30864407252103	0.75090962945785	0.00000000000000
C	2.78651280224242	-0.58792252947891	0.00000000000000
N	2.22313064694131	1.62504950918835	0.00000000000000
C	4.66758853968082	1.03346677935772	0.00000000000000
C	3.70484213063845	-1.66821895393415	0.00000000000000
C	5.06214147055726	-1.40532371219940	0.00000000000000
C	5.57466953557336	-0.06166472412699	0.00000000000000
N	-8.69088361176604	1.78419069669428	0.00000000000000
H	5.03701436376569	2.05728804765099	0.00000000000000
H	-3.34977911546207	2.69522716283142	0.00000000000000
C	1.35654827549472	-0.47403144233418	0.00000000000000
H	3.34977911546207	-2.69522716283142	0.00000000000000
H	5.76920634280081	-2.23195802021605	0.00000000000000
C	1.05154799406088	0.90329670863978	0.00000000000000
C	-0.29398848268777	1.47988841309205	0.00000000000000
C	-1.35654827549472	0.47403144233418	0.00000000000000
C	-1.05154799406088	-0.90329670863978	0.00000000000000
C	0.29398848268777	-1.47988841309205	0.00000000000000
H	2.27090286408032	2.63952163619470	0.00000000000000
H	-2.27090286408032	-2.63952163619470	0.00000000000000
C	-2.78651280224242	0.58792252947891	0.00000000000000
C	-3.30864407252103	-0.75090962945785	0.00000000000000
N	-2.22313064694131	-1.62504950918835	0.00000000000000
N	-7.92715585709660	-2.59302237917633	0.00000000000000
C	-3.70484213063845	1.66821895393415	0.00000000000000
C	-5.06214147055726	1.40532371219940	0.00000000000000
C	-5.57466953557336	0.06166472412699	0.00000000000000
C	-4.66758853968082	-1.03346677935772	0.00000000000000
N	7.92715585709660	2.59302237917633	0.00000000000000
H	-5.76920634280081	2.23195802021605	0.00000000000000
H	-5.03701436376569	-2.05728804765099	0.00000000000000
C	6.99976271844278	0.17576596616868	0.00000000000000
C	7.93271636884312	-0.89134505839524	0.00000000000000
C	7.52543919418451	1.49283147836236	0.00000000000000
N	8.69088361176604	-1.78419069669428	0.00000000000000
C	-6.99976271844278	-0.17576596616868	0.00000000000000
C	-7.93271636884312	0.89134505839524	0.00000000000000
C	-7.52543919418451	-1.49283147836236	0.00000000000000
C	0.48553042635758	-2.80603428525950	0.00000000000000
C	-0.48553042635758	2.80603428525950	0.00000000000000
C	0.62243446101925	-4.09596605871004	0.00000000000000
C	-0.62243446101925	4.09596605871004	0.00000000000000
F	-0.71539444004620	4.88334908201904	1.10412919812405
F	-0.71539444004620	4.88334908201904	-1.10412919812405
F	0.71539444004620	-4.88334908201904	-1.10412919812405
F	0.71539444004620	-4.88334908201904	1.10412919812405

The closed-shell singlet-optimized C_{2h} geometry of molecule **PT** is given below (BLYP/TZP level of theory). Total bonding energy: -14.034219539151330 $a.u.$

```
52
C34H8N10 optimized with ADF in AMS.
C       -3.13991461678326      -1.25777203865049       0.00000000000000
C       -2.83162380656098       0.15053815928438       0.00000000000000
N       -1.92730828184161      -1.95539594428879       0.00000000000000
C       -4.42772633521261      -1.75271937532578       0.00000000000000
C       -3.91347404190284       1.07502407264818       0.00000000000000
C       -5.20745944817074       0.60215217901541       0.00000000000000
C       -5.50579474922934      -0.81103324232995       0.00000000000000
N        8.85221933311536      -0.38633532489628       0.00000000000000
H       -4.63058651358152      -2.82199035829850       0.00000000000000
H        3.72825006548884      -2.14566448231580       0.00000000000000
C       -1.41252135509042       0.26400652857412       0.00000000000000
H       -3.72825006548884       2.14566448231580       0.00000000000000
H       -6.03608765431594       1.30683252125032       0.00000000000000
C       -0.88275440013224      -1.06676870283138       0.00000000000000
C        0.51665510418595      -1.39829884248880       0.00000000000000
C        1.41252135509042      -0.26400652857412       0.00000000000000
C        0.88275440013224       1.06676870283138       0.00000000000000
C       -0.51665510418595       1.39829884248880       0.00000000000000
H       -1.82504785115932      -2.96689018735206       0.00000000000000
H        1.82504785115932       2.96689018735206       0.00000000000000
C        2.83162380656098      -0.15053815928438       0.00000000000000
C        3.13991461678326       1.25777203865049       0.00000000000000
N        1.92730828184161       1.95539594428879       0.00000000000000
N        7.39216035691590       3.80301457215886       0.00000000000000
C        3.91347404190284      -1.07502407264818       0.00000000000000
C        5.20745944817074      -0.60215217901541       0.00000000000000
C        5.50579474922934       0.81103324232995       0.00000000000000
C        4.42772633521261       1.75271937532578       0.00000000000000
N       -7.39216035691590      -3.80301457215886       0.00000000000000
H        6.03608765431594      -1.30683252125032       0.00000000000000
H        4.63058651358152       2.82199035829850       0.00000000000000
C       -6.85907281316796      -1.26785810261426       0.00000000000000
C       -7.95461958933721      -0.36473447413249       0.00000000000000
C       -7.16918107857666      -2.65405561780528       0.00000000000000
N       -8.85221933311536       0.38633532489628       0.00000000000000
C        6.85907281316796       1.26785810261426       0.00000000000000
C        7.95461958933721       0.36473447413249       0.00000000000000
C        7.16918107857666       2.65405561780528       0.00000000000000
C       -0.92535359815058       2.70908907489918       0.00000000000000
C        0.92535359815058      -2.70908907489918       0.00000000000000
C       -1.22984395352602       3.91380012299450       0.00000000000000
C        1.22984395352602      -3.91380012299450       0.00000000000000
C        1.58721424434189      -5.23514113176230       0.00000000000000
C       -1.58721424434189       5.23514113176230       0.00000000000000
N        4.09946290215672      -5.88892968643466       0.00000000000000
N       -0.26335030970257      -7.05585474145157       0.00000000000000
N        0.26335030970257       7.05585474145157       0.00000000000000
N       -4.09946290215672       5.88892968643466       0.00000000000000
C        0.58250501624613      -6.24922702434560       0.00000000000000
C        2.96460587976231      -5.60961925132167       0.00000000000000
C       -2.96460587976231       5.60961925132167       0.00000000000000
C       -0.58250501624613       6.24922702434560       0.00000000000000
```

The triplet-optimized C_{2h} geometry of molecule **PT** is given below (BLYP/TZP level of theory).
Total bonding energy: -14.035992926738412 $a.u.$

```
52
C34H8N10 optimized with ADF in AMS.
C       3.30087532706971      0.76279016694442      0.00000000000000
C       2.78351540842511     -0.57932240954348      0.00000000000000
N       2.21592908688776      1.63694488719164      0.00000000000000
C       4.65872456758344      1.04937471118233      0.00000000000000
C       3.70368177715286     -1.65831345452638      0.00000000000000
C       5.05947726575576     -1.39095467829958      0.00000000000000
C       5.56785807695685     -0.04420605341645      0.00000000000000
N      -8.69129464626383      1.75692684414900      0.00000000000000
H       5.02483326076968      2.07431546522084      0.00000000000000
H      -3.35551183089494      2.68752499118158      0.00000000000000
C       1.35313744372383     -0.47085875133142      0.00000000000000
H       3.35551183089494     -2.68752499118158      0.00000000000000
H       5.76788894850086     -2.21642584043437      0.00000000000000
C       1.04111121937359      0.91643963598778      0.00000000000000
C      -0.29437126510464      1.45774533079638      0.00000000000000
C      -1.35313744372383      0.47085875133142      0.00000000000000
C      -1.04111121937359     -0.91643963598778      0.00000000000000
C       0.29437126510464     -1.45774533079638      0.00000000000000
H       2.27172789288512      2.65157285997206      0.00000000000000
H      -2.27172789288512     -2.65157285997206      0.00000000000000
C      -2.78351540842511      0.57932240954348      0.00000000000000
C      -3.30087532706971     -0.76279016694442      0.00000000000000
N      -2.21592908688776     -1.63694488719164      0.00000000000000
N      -7.91700477490582     -2.61234438326519      0.00000000000000
C      -3.70368177715286      1.65831345452638      0.00000000000000
C      -5.05947726575576      1.39095467829958      0.00000000000000
C      -5.56785807695685      0.04420605341645      0.00000000000000
C      -4.65872456758344     -1.04937471118233      0.00000000000000
N       7.91700477490582      2.61234438326519      0.00000000000000
H      -5.76788894850086      2.21642584043437      0.00000000000000
H      -5.02483326076968     -2.07431546522084      0.00000000000000
C       6.99052971623162      0.19464502811222      0.00000000000000
C       7.92684472237357     -0.86988807631130      0.00000000000000
C       7.51463501353603      1.51278863305324      0.00000000000000
N       8.69129464626383     -1.75692684414900      0.00000000000000
C      -6.99052971623162     -0.19464502811222      0.00000000000000
C      -7.92684472237357      0.86988807631130      0.00000000000000
C      -7.51463501353603     -1.51278863305324      0.00000000000000
C       0.49956889865691     -2.81323301642341      0.00000000000000
C      -0.49956889865691      2.81323301642341      0.00000000000000
C       0.61773029198604     -4.05092260460155      0.00000000000000
C      -0.61773029198604      4.05092260460155      0.00000000000000
C      -0.77209035863651      5.40958260593715      0.00000000000000
C       0.77209035863651     -5.40958260593715      0.00000000000000
N      -3.15675394434030      6.43662710340000      0.00000000000000
N       1.32797478313394      6.93664050742106      0.00000000000000
N      -1.32797478313394     -6.93664050742106      0.00000000000000
N       3.15675394434030     -6.43662710340000      0.00000000000000
C       0.37312729491465      6.26284335764995      0.00000000000000
C      -2.07777027886743      5.98785378826183      0.00000000000000
C       2.07777027886743     -5.98785378826183      0.00000000000000
C      -0.37312729491465     -6.26284335764995      0.00000000000000
```

The quintet-optimized C_{2h} geometry of molecule **PT** is given below (BLYP/TZP level of theory).
Total bonding energy: -14.009654268262725 *a.u.*

```
52
C34H8N10 optimized with ADF in AMS.
C        3.29638750761054       0.75457758368672       0.00000000000000
C        2.78421624903653      -0.58484563872934       0.00000000000000
N        2.21254538349437       1.63567854051554       0.00000000000000
C        4.65478506118496       1.04139032002234       0.00000000000000
C        3.70230601067640      -1.66056054291700       0.00000000000000
C        5.06260661549522      -1.39413749303238       0.00000000000000
C        5.56498719008057      -0.05106945206344       0.00000000000000
N       -8.69691078175601       1.75210217361503       0.00000000000000
H        5.02001950470274       2.06664482366073       0.00000000000000
H       -3.35418038421212       2.68986290091331       0.00000000000000
C        1.34469675847859      -0.48036153948344       0.00000000000000
H        3.35418038421212      -2.68986290091331       0.00000000000000
H        5.77020694829125      -2.22012169261401       0.00000000000000
C        1.03265050575645       0.92161972046328       0.00000000000000
C       -0.29352695942475       1.44516769962503       0.00000000000000
C       -1.34469675847859       0.48036153948344       0.00000000000000
C       -1.03265050575645      -0.92161972046328       0.00000000000000
C        0.29352695942475      -1.44516769962503       0.00000000000000
H        2.27444020949852       2.64938705529995       0.00000000000000
H       -2.27444020949852      -2.64938705529995       0.00000000000000
C       -2.78421624903653       0.58484563872934       0.00000000000000
C       -3.29638750761054      -0.75457758368672       0.00000000000000
N       -2.21254538349437      -1.63567854051554       0.00000000000000
N       -7.90759761952240      -2.61663979114684       0.00000000000000
C       -3.70230601067640       1.66056054291700       0.00000000000000
C       -5.06260661549522       1.39413749303238       0.00000000000000
C       -5.56498719008057       0.05106945206344       0.00000000000000
C       -4.65478506118496      -1.04139032002234       0.00000000000000
N        7.90759761952240       2.61663979114684       0.00000000000000
H       -5.77020694829125       2.22012169261401       0.00000000000000
H       -5.02001950470274      -2.06664482366073       0.00000000000000
C        6.99290246761193       0.19546503006473       0.00000000000000
C        7.93135825009862      -0.86547199039197       0.00000000000000
C        7.51055363160442       1.51463759317197       0.00000000000000
N        8.69691078175601      -1.75210217361503       0.00000000000000
C       -6.99290246761193      -0.19546503006473       0.00000000000000
C       -7.93135825009862       0.86547199039197       0.00000000000000
C       -7.51055363160442      -1.51463759317197       0.00000000000000
C        0.50553120546131      -2.83223697844193       0.00000000000000
C       -0.50553120546131       2.83223697844193       0.00000000000000
C        0.62311275623067      -4.05778583286873       0.00000000000000
C       -0.62311275623067       4.05778583286873       0.00000000000000
C       -0.77874757433331       5.44067030330904       0.00000000000000
C        0.77874757433331      -5.44067030330904       0.00000000000000
N       -3.16036904167304       6.46154648267553       0.00000000000000
N        1.32524636168294       6.95338927283506       0.00000000000000
N       -1.32524636168294      -6.95338927283506       0.00000000000000
N        3.16036904167304      -6.46154648267553       0.00000000000000
C        0.36397839284641       6.28493299382805       0.00000000000000
C       -2.07816939524671       6.01482120530701       0.00000000000000
C        2.07816939524671      -6.01482120530701       0.00000000000000
C       -0.36397839284641      -6.28493299382805       0.00000000000000
```

The closed-shell singlet-optimized C_{2h} geometry of molecule **PTP** is given below (BLYP/TZP level of theory). Total bonding energy: -18.804272827229461 $a.u.$

```
72
C46H16N10 optimized with ADF in AMS.
C        0.01460495474586       3.37650019411525       0.00000000000000
C       -1.17987717089866       2.56621267110093       0.00000000000000
N        1.11115070045243       2.51223488802087       0.00000000000000
C       -0.00917756295390       4.75491430587929       0.00000000000000
C       -2.43987072655990       3.22836287211793       0.00000000000000
C       -2.48587224507534       4.60431932752821       0.00000000000000
C       -1.28444152461256       5.40698046291511       0.00000000000000
N        3.65036471685207      -8.05221101510433       0.00000000000000
H        0.90628906221006       5.34334140443041       0.00000000000000
H        3.35910045678118      -2.64968833247517       0.00000000000000
C       -0.76666607430249       1.20599154404989       0.00000000000000
H       -3.35910045678118       2.64968833247517       0.00000000000000
H       -3.44689732165818       5.11362996497418       0.00000000000000
C        0.67483474333201       1.20550506196598       0.00000000000000
C        1.49101156567975       0.04101481364978       0.00000000000000
C        0.76666607430249      -1.20599154404989       0.00000000000000
C       -0.67483474333201      -1.20550506196598       0.00000000000000
C       -1.49101156567975      -0.04101481364978       0.00000000000000
H        2.08795385733848       2.78755580026639       0.00000000000000
H       -2.08795385733848      -2.78755580026639       0.00000000000000
C        1.17987717089866      -2.56621267110093       0.00000000000000
C       -0.01460495474586      -3.37650019411525       0.00000000000000
N       -1.11115070045243      -2.51223488802087       0.00000000000000
N       -0.79155507744979      -8.26687358297685       0.00000000000000
C        2.43987072655990      -3.22836287211793       0.00000000000000
C        2.48587224507534      -4.60431932752821       0.00000000000000
C        1.28444152461256      -5.40698046291511       0.00000000000000
C        0.00917756295390      -4.75491430587929       0.00000000000000
N        0.79155507744979       8.26687358297685       0.00000000000000
H        3.44689732165818      -5.11362996497418       0.00000000000000
H       -0.90628906221006      -5.34334140443041       0.00000000000000
C       -1.36535705547405       6.83140880310352       0.00000000000000
C       -2.61368334531685       7.50906492365393       0.00000000000000
C       -0.19392948722354       7.63551992756421       0.00000000000000
N       -3.65036471685207       8.05221101510433       0.00000000000000
C        1.36535705547405      -6.83140880310352       0.00000000000000
C        2.61368334531685      -7.50906492365393       0.00000000000000
C        0.19392948722354      -7.63551992756421       0.00000000000000
C       -2.87976290858596      -0.14728328299930       0.00000000000000
C        2.87976290858596       0.14728328299930       0.00000000000000
C       -4.10295399408993      -0.31367785465421       0.00000000000000
C        4.10295399408993       0.31367785465421       0.00000000000000
C        9.74211010107246       0.97839330231812       0.00000000000000
H        8.38051985150985      -1.37171878471863       0.00000000000000
H        5.94133449494942      -1.65820637764802       0.00000000000000
H        7.87167727870631       2.94663010139036       0.00000000000000
H       -5.94133449494942       1.65820637764802       0.00000000000000
C        5.48884456077347       0.47626330488259       0.00000000000000
C        6.08021234670758       1.78284246938419       0.00000000000000
C        7.44618086706917       1.94491568841486       0.00000000000000
C        8.32712435726921       0.81109638314825       0.00000000000000
C        7.73373367976393      -0.49639640679737       0.00000000000000
C        6.36748655094317      -0.65726320377516       0.00000000000000
H        5.43275922424298       2.65754891094557       0.00000000000000
C       -5.48884456077347      -0.47626330488259       0.00000000000000
```

```
C       -6.08021234670758      -1.78284246938419       0.00000000000000
C       -7.44618086706917      -1.94491568841486       0.00000000000000
C       -8.32712435726921      -0.81109638314825       0.00000000000000
C       -7.73373367976393       0.49639640679737       0.00000000000000
C       -6.36748655094317       0.65726320377516       0.00000000000000
H       -5.43275922424298      -2.65754891094557       0.00000000000000
H       -7.87167727870631      -2.94663010139036       0.00000000000000
C       -9.74211010107246      -0.97839330231812       0.00000000000000
H       -8.38051985150985       1.37171878471863       0.00000000000000
C       10.33601913831483       2.26940526308283       0.00000000000000
N       10.80307267565967       3.34244316156538       0.00000000000000
C       10.62189354279937      -0.13746546331573       0.00000000000000
N       11.32912344373901      -1.06991746981778       0.00000000000000
C      -10.62189354279937       0.13746546331573       0.00000000000000
N      -11.32912344373901       1.06991746981778       0.00000000000000
C      -10.33601913831483      -2.26940526308283       0.00000000000000
N      -10.80307267565967      -3.34244316156538       0.00000000000000
```

The triplet-optimized C_{2h} geometry of molecule **PTP** is given below (BLYP/TZP level of theory).
Total bonding energy: -18.805164420570478 *a.u.*

```
72
C46H16N10 optimized with ADF in AMS.
C        0.01368222559578       3.37784051044906       0.00000000000000
C       -1.17983689425813       2.56151704925816       0.00000000000000
N        1.11119880458779       2.52079612298567       0.00000000000000
C       -0.01951818195328       4.75691985134764       0.00000000000000
C       -2.44388261415288       3.21807379301955       0.00000000000000
C       -2.49636460278289       4.59258297164931       0.00000000000000
C       -1.29702412185564       5.40152408847169       0.00000000000000
N        3.68352447725684      -8.03042443472226       0.00000000000000
H        0.89245633858134       5.35066452387438       0.00000000000000
H        3.35990204029146      -2.63439763685460       0.00000000000000
C       -0.76429796839585       1.20403400202230       0.00000000000000
H       -3.35990204029146       2.63439763685460       0.00000000000000
H       -3.45947218446939       5.09798252254808       0.00000000000000
C        0.67912531027332       1.20679484319527       0.00000000000000
C        1.48661137963458       0.04708908813785       0.00000000000000
C        0.76429796839585      -1.20403400202230       0.00000000000000
C       -0.67912531027332      -1.20679484319527       0.00000000000000
C       -1.48661137963458      -0.04708908813785       0.00000000000000
H        2.08603458651333       2.80114983784716       0.00000000000000
H       -2.08603458651333      -2.80114983784716       0.00000000000000
C        1.17983689425813      -2.56151704925816       0.00000000000000
C       -0.01368222559578      -3.37784051044906       0.00000000000000
N       -1.11119880458779      -2.52079612298567       0.00000000000000
N       -0.75450646570854      -8.27883770217058       0.00000000000000
C        2.44388261415288      -3.21807379301955       0.00000000000000
C        2.49636460278289      -4.59258297164931       0.00000000000000
C        1.29702412185564      -5.40152408847169       0.00000000000000
C        0.01951818195328      -4.75691985134764       0.00000000000000
N        0.75450646570854       8.27883770217058       0.00000000000000
H        3.45947218446939      -5.09798252254808       0.00000000000000
H       -0.89245633858134      -5.35066452387438       0.00000000000000
C       -1.39015757963390       6.82458923293072       0.00000000000000
C       -2.64369503458914       7.49343710248941       0.00000000000000
C       -0.22510504487972       7.63857849556584       0.00000000000000
N       -3.68352447725684       8.03042443472226       0.00000000000000
```

```
C        1.39015757963390      -6.82458923293072       0.00000000000000
C        2.64369503458914      -7.49343710248941       0.00000000000000
C        0.22510504487972      -7.63857849556584       0.00000000000000
C       -2.88370227629380      -0.14933922721705       0.00000000000000
C        2.88370227629380       0.14933922721705       0.00000000000000
C       -4.10365499693526      -0.31073956778355       0.00000000000000
C        4.10365499693526       0.31073956778355       0.00000000000000
C        9.75732272820050       0.96242825084569       0.00000000000000
H        8.38430740083187      -1.38277625839932       0.00000000000000
H        5.94220066560625      -1.66310393729165       0.00000000000000
H        7.88671918550063       2.93251357279469       0.00000000000000
H       -5.94220066560625       1.66310393729165       0.00000000000000
C        5.49856716524699       0.47040313759363       0.00000000000000
C        6.08956223802005       1.77234521191836       0.00000000000000
C        7.45968429245210       1.93146408829234       0.00000000000000
C        8.33305656255603       0.79761082146589       0.00000000000000
C        7.74055094132164      -0.50523345160319       0.00000000000000
C        6.37012557810938      -0.66303531961420       0.00000000000000
H        5.44476507926061       2.64883579093383       0.00000000000000
C       -5.49856716524699      -0.47040313759363       0.00000000000000
C       -6.08956223802005      -1.77234521191836       0.00000000000000
C       -7.45968429245210      -1.93146408829234       0.00000000000000
C       -8.33305656255603      -0.79761082146589       0.00000000000000
C       -7.74055094132164       0.50523345160319       0.00000000000000
C       -6.37012557810938       0.66303531961420       0.00000000000000
H       -5.44476507926061      -2.64883579093383       0.00000000000000
H       -7.88671918550063      -2.93251357279469       0.00000000000000
C       -9.75732272820050      -0.96242825084569       0.00000000000000
H       -8.38430740083187       1.38277625839932       0.00000000000000
C       10.35320855283960       2.25101971143153       0.00000000000000
N       10.82206355851435       3.32357347321866       0.00000000000000
C       10.63304208345513      -0.15485080183563       0.00000000000000
N       11.33764867521844      -1.08974966518893       0.00000000000000
C      -10.63304208345513       0.15485080183563       0.00000000000000
N      -11.33764867521844       1.08974966518893       0.00000000000000
C      -10.35320855283960      -2.25101971143153       0.00000000000000
N      -10.82206355851435      -3.32357347321866       0.00000000000000
```

The quintet-optimized C_{2h} geometry of molecule **PTP** is given below (BLYP/TZP level of theory).
Total bonding energy: -18.797140365861516 *a.u.*

```
72
C46H16N10 optimized with ADF in AMS.
C        0.00643515340622       3.37826795057296       0.00000000000000
C       -1.18560529424071       2.57882443460124       0.00000000000000
N        1.10403774321341       2.52000057391310       0.00000000000000
C       -0.02119778300087       4.76643873224609       0.00000000000000
C       -2.43874319043890       3.23440236096129       0.00000000000000
C       -2.48643021633779       4.61864097041178       0.00000000000000
C       -1.28935600546168       5.40996808715648       0.00000000000000
N        3.65333809292614      -8.07236475824837       0.00000000000000
H        0.89511505605194       5.35373241224626       0.00000000000000
H        3.35865746736626      -2.65652471693477       0.00000000000000
C       -0.76633233490131       1.19854257669916       0.00000000000000
H       -3.35865746736626       2.65652471693477       0.00000000000000
H       -3.44847050269608       5.12596325407791       0.00000000000000
C        0.66913935530420       1.20607253815175       0.00000000000000
C        1.47934342554760       0.03870546118421       0.00000000000000
```

C	0.76633233490131	-1.19854257669916	0.00000000000000
C	-0.66913935530420	-1.20607253815175	0.00000000000000
C	-1.47934342554760	-0.03870546118421	0.00000000000000
H	2.07919665413033	2.79861953056489	0.00000000000000
H	-2.07919665413033	-2.79861953056489	0.00000000000000
C	1.18560529424071	-2.57882443460124	0.00000000000000
C	-0.00643515340622	-3.37826795057296	0.00000000000000
N	-1.10403774321341	-2.52000057391310	0.00000000000000
N	-0.78811861366206	-8.28522067104072	0.00000000000000
C	2.43874319043890	-3.23440236096129	0.00000000000000
C	2.48643021633779	-4.61864097041178	0.00000000000000
C	1.28935600546168	-5.40996808715648	0.00000000000000
C	0.02119778300087	-4.76643873224609	0.00000000000000
N	0.78811861366206	8.28522067104072	0.00000000000000
H	3.44847050269608	-5.12596325407791	0.00000000000000
H	-0.89511505605194	-5.35373241224626	0.00000000000000
C	-1.36934765675879	6.85479158838621	0.00000000000000
C	-2.61478004940291	7.53062967607841	0.00000000000000
C	-0.19926812840179	7.65512139091326	0.00000000000000
N	-3.65333809292614	8.07236475824837	0.00000000000000
C	1.36934765675879	-6.85479158838621	0.00000000000000
C	2.61478004940291	-7.53062967607841	0.00000000000000
C	0.19926812840179	-7.65512139091326	0.00000000000000
C	-2.88307426422434	-0.14747224696756	0.00000000000000
C	2.88307426422434	0.14747224696756	0.00000000000000
C	-4.09927292419589	-0.31415481077973	0.00000000000000
C	4.09927292419589	0.31415481077973	0.00000000000000
C	9.76298495594650	0.97924846531083	0.00000000000000
H	8.38971672916676	-1.36748417535800	0.00000000000000
H	5.94582568588618	-1.65253827352582	0.00000000000000
H	7.88381635680226	2.94335051091979	0.00000000000000
H	-5.94582568588618	1.65253827352582	0.00000000000000
C	5.50111735797724	0.47820741268003	0.00000000000000
C	6.08656390708858	1.77865265831601	0.00000000000000
C	7.45945354728187	1.94112308528297	0.00000000000000
C	8.33168927505058	0.81076947984367	0.00000000000000
C	7.74473466008886	-0.49079571972791	0.00000000000000
C	6.37152550317442	-0.65153728196386	0.00000000000000
H	5.44043286186097	2.65413544621793	0.00000000000000
C	-5.50111735797724	-0.47820741268003	0.00000000000000
C	-6.08656390708858	-1.77865265831601	0.00000000000000
C	-7.45945354728187	-1.94112308528297	0.00000000000000
C	-8.33168927505058	-0.81076947984367	0.00000000000000
C	-7.74473466008886	0.49079571972791	0.00000000000000
C	-6.37152550317442	0.65153728196386	0.00000000000000
H	-5.44043286186097	-2.65413544621793	0.00000000000000
H	-7.88381635680226	-2.94335051091979	0.00000000000000
C	-9.76298495594650	-0.97924846531083	0.00000000000000
H	-8.38971672916676	1.36748417535800	0.00000000000000
C	10.35502509780306	2.26799167517209	0.00000000000000
N	10.82062845254428	3.34252708499749	0.00000000000000
C	10.63912718236024	-0.13588842768511	0.00000000000000
N	11.34413648533630	-1.07096548660261	0.00000000000000
C	-10.63912718236024	0.13588842768511	0.00000000000000
N	-11.34413648533630	1.07096548660261	0.00000000000000
C	-10.35502509780306	-2.26799167517209	0.00000000000000
N	-10.82062845254428	-3.34252708499749	0.00000000000000

References

(1) Badía-Domínguez, I.; Canola, S.; Hernández Jolín, V.; López Navarrete, J. T.; Sancho-García, J. C.; Negri, F.; Ruiz Delgado, M. C. Tuning the Diradical Character of Indolocarbazoles: Impact of Structural Isomerism and Substitution Position. *J. Phys. Chem. Lett.* **2022**, *13*, 6003–6010.

(2) Zhang, Y.; Ma, Y.; Kong, L.; Tian, Y.; Yang, J. A novel indolo[3,2-b]carbazole derivative with D-π-A structure exhibiting aggregation-enhanced emission and mechanofluorochromic properties. *Dyes Pigm.* **2018**, *159*, 314–321.

(3) Clar, E. *The aromatic sextet*; New York, Wiley, 1972.

(4) Betkhoshvili, S.; de P. R. Moreira, I.; Poater, J.; Maria Bofill, J. Pathway to Polyradicals: A Planar and Fully π-Conjugated Organic Tetraradical(oid). *J. Phys. Chem. Lett.* **2024**, *15*, 5243–5249.

(5) te Velde, G.; Bickelhaupt, F. M.; Baerends, E. J.; Fonseca Guerra, C.; van Gisbergen, S. J. A.; Snijders, J. G.; Ziegler, T. Chemistry with ADF. *J. Comput. Chem.* **2001**, *22*, 931–967.

(6) Becke, A. D. A multicenter numerical integration scheme for polyatomic molecules. *J. Chem. Phys.* **1988**, *88*, 2547–2553.

(7) Franchini, M.; Philipsen, P. H. T.; Visscher, L. The Becke Fuzzy Cells Integration Scheme in the Amsterdam Density Functional Program Suite. *J. Comput. Chem.* **2013**, *34*, 1819–1827.

(8) Seth, M.; Ziegler, T. Range-Separated Exchange Functionals with Slater-Type Functions. *J. Chem. Theory Comput.* **2012**, *8*, 901–907.

(9) Förster, A.; Franchini, M.; van Lenthe, E.; Visscher, L. A Quadratic Pair Atomic Resolution of the Identity Based SOS-AO-MP2 Algorithm Using Slater Type Orbitals. *J. Chem. Theory Comput.* **2020**, *16*, 875–891.

(10) Förster, A.; Visscher, L. Double hybrid DFT calculations with Slater type orbitals. *J. Comput. Chem.* **2020**, *41*, 1660–1684.

(11) Van Lenthe, E.; Baerends, E. J. Optimized Slater-type basis sets for the elements 1–118. *J. Comput. Chem.* **2003**, *24*, 1142–1156.

(12) Jacobsen, H.; Bérces, A.; Swerhone, D. P.; Ziegler, T. Analytic second derivatives of molecular energies: a density functional implementation. *Comput. Phys. Commun.* **1997**, *100*, 263–276.

(13) Fan, L.; Ziegler, T. Application of density functional theory to infrared absorption intensity calculations on main group molecules. *J. Chem. Phys.* **1992**, *96*, 9005–9012.

(14) Fan, L.; Ziegler, T. Application of density functional theory to infrared absorption intensity calculations on transition-metal carbonyls. *J. Phys. Chem.* **1992**, *96*, 6937–6941.

(15) Becke, A. D. Density-functional exchange-energy approximation with correct asymptotic behavior. *Phys. Rev. A* **1988**, *38*, 3098–3100.

(16) Lee, C.; Yang, W.; Parr, R. G. Development of the Colle-Salvetti correlation-energy formula into a functional of the electron density. *Phys. Rev. B* **1988**, *37*, 785–789.

(17) Frisch, M. J. *et al.* Gaussian~16 Revision B.01. 2016; Gaussian Inc. Wallingford CT.

(18) Woon, D. E.; Dunning, J., Thom H. Gaussian basis sets for use in correlated molecular calculations. V. Core-valence basis sets for boron through neon. *J. Chem. Phys.* **1995**, *103*, 4572–4585.

(19) Cheung, L. M.; Sundberg, K. R.; Ruedenberg, K. Dimerization of carbene to ethylene. *J. Am. Chem. Soc.* **1978**, *100*, 8024–8025.

(20) Cheung, L. M.; Sundberg, K. R.; Ruedenberg, K. Electronic rearrangements during chemical reactions. II. Planar dissociation of ethylene. *Int. J. Quantum Chem.* **1979**, *16*, 1103–1139.

(21) Roos, B. O.; Taylor, P. R.; Sigbahn, P. E. A complete active space SCF method (CASSCF) using a density matrix formulated super-CI approach. *J. Chem. Phys.* **1980**, *48*, 157–173.

(22) Siegbahn, P.; Heiberg, A.; Roos, B.; Levy, B. A Comparison of the Super-CI and the Newton-Raphson Scheme in the Complete Active Space SCF Method. *Phys. Scr.* **1980**, *21*, 323.

(23) Johnson, R. P.; Schmidt, M. W. The sudden polarization effect: MC-SCF calculations on planar and 90.degree. twisted methylenecyclopropene. *J. Am. Chem. Soc.* **1981**, *103*, 3244–3249.

(24) Siegbahn, P. E. M.; Almlöf, J.; Heiberg, A.; Roos, B. O. The complete active space SCF (CASSCF) method in a Newton–Raphson formulation with application to the HNO molecule. *J. Chem. Phys.* **1981**, *74*, 2384–2396.

(25) Feller, D. F.; Schmidt, M. W.; Ruedenberg, K. Concerted dihydrogen exchange between ethane and ethylene. SCF and FORS calculations of the barrier. *J. Am. Chem. Soc.* **1982**, *104*, 960–967.

(26) Barca, G. M. J. *et al.* Recent developments in the general atomic and molecular electronic structure system. *J. Chem. Phys.* **2020**, *152*, 154102.

(27) Pulay, P.; Hamilton, T. P. UHF natural orbitals for defining and starting MC-SCF calculations. *J. Chem. Phys.* **1988**, *88*, 4926–4933.

(28) Bofill, J. M.; Pulay, P. The unrestricted natural orbital–complete active space (UNO–CAS) method: An inexpensive alternative to the complete active space–self-consistent-field (CAS–SCF) method. *J. Chem. Phys.* **1989**, *90*, 3637–3646.

(29) Tóth, Z.; Pulay, P. Comparison of Methods for Active Orbital Selection in Multiconfigurational Calculations. *J. Chem. Theory Comput.* **2020**, *16*, 7328–7341.

(30) Lowdin, P.-O. Present Situation of Quantum Chemistry. *J. Phys. Chem.* **1957**, *61*, 55–68.

(31) Heisenberg, W. Zur Theorie des Ferromagnetismus. *Z. Phys.* **1928**, *49*, 619–636.

(32) Dirac, P. A. M.; Polkinghorne, J. C. The Principles of Quantum Mechanics. *Phys. Today* **1958**, *11*, 32–33.

(33) Van Vleck, J. *The Theory of Electric and Magnetic Susceptibilities*; Internat. Ser. Monogr. Phys.; Clarendon Press, 1932.

(34) de P. R. Moreira, I.; Illas, F. Ab initio theoretical comparative study of magnetic coupling in $KNiF_3$sand K_2 NiF_4s. *Phys. Rev. B* **1997**, *55*, 4129–4137.

(35) Bloch, C. Sur la théorie des perturbations des états liés. *Nucl. Phys.* **1958**, *6*, 329–347.

(36) des Cloizeaux, J. Extension d'une formule de Lagrange à des problèmes de valeurs propres. *Nucl. Phys.* **1960**, *20*, 321–346.

(37) Geuenich, D.; Hess, K.; Köhler, F.; Herges, R. Anisotropy of the Induced Current Density (ACID), a General Method To Quantify and Visualize Electronic Delocalization. *Chem. Rev.* **2005**, *105*, 3758–3772.

(38) Poater, J.; Duran, M.; Solà, M. Aromaticity Determines the Relative Stability of Kinked vs. Straight Topologies in Polycyclic Aromatic Hydrocarbons. *Front. Chem.* **2018**, *6*.

(39) Becke, A. D. Density-functional thermochemistry. III. The role of exact exchange. *J. Chem. Phys.* **1993**, *98*, 5648–5652.

(40) Lee, C.; Yang, W.; Parr, R. G. Development of the Colle-Salvetti correlation-energy formula into a functional of the electron density. *Phys. Rev. B* **1988**, *37*, 785–789.

(41) Vosko, S. H.; Wilk, L.; Nusair, M. Accurate spin-dependent electron liquid correlation energies for local spin density calculations: a critical analysis. *Can. J. Phys.* **1980**, *58*, 1200–1211.

(42) Stephens, P. J.; Devlin, F. J.; Chabalowski, C. F.; Frisch, M. J. Ab Initio Calculation of Vibrational Absorption and Circular Dichroism Spectra Using Density Functional Force Fields. *J. Phys. Chem.* **1994**, *98*, 11623–11627.

(43) Bultinck, P.; Ponec, R.; Van Damme, S. Multicenter bond indices as a new measure of aromaticity in polycyclic aromatic hydrocarbons. *J. Phys. Org. Chem.* **2005**, *18*, 706–718.

(44) Feixas, F.; Matito, E.; Poater, J.; Solà, M. Quantifying aromaticity with electron delocalisation measure. *Chem. Soc. Rev.* **2015**, *44*, 6434–6451.

(45) Yamaguchi, K. The electronic structures of biradicals in the unrestricted Hartree-Fock approximation. *Chem. Phys. Lett.* **1975**, *33*, 330–335.

(46) Schmidt, E. Zur Theorie der linearen und nichtlinearen Integralgleichungen. *Math. Ann.* **1907**, *63*, 433–476.
